# Aluminylation: a generalizable route towards low-valent aluminum under moderate conditions with controlled product nuclearity through precursor design

**DOI:** 10.1039/d6sc02187e

**Published:** 2026-04-13

**Authors:** Paris C. Reuel, Yogesh Shandilya, M. Talha Wattoo, Alison B. Altman

**Affiliations:** a Department of Chemistry, Texas A&M University College Station TX 77843 USA aaltman@tamu.edu

## Abstract

Recent advances in isolating and characterizing reactive aluminylenes and aluminyl anions have redefined the landscape of low-valent aluminum chemistry. Study of these molecules that span a wide range of structures and ligand environments have helped established new paradigms in main-group metal bonding and reactivity; however, their controlled access remains limited by aggregation, disproportionation, and oxidation of reactive Al(i) intermediates. Herein, we establish aluminylation as a generalizable redox-neutral synthetic route to low-valent aluminum complexes. By circumventing the use of external reductants through metathesis reactions between Al(i) precursors and alkali metal ligand salts, we can access novel heteroleptic clusters as well as known aluminyl complexes with improved yields or under more mild conditions. We demonstrate that the equilibrium of the Al(i) precursor between monomeric or oligomeric cyclopentadienyl aluminum species directly governs reaction outcomes and enables selective formation of products across multiple ligand classes.

## Abstract 2

Aluminylation enables redox-neutral access to low-valent aluminum complexes with nuclearity and heterolepticity tunable by substrate identity and Al(i) speciation.

## Graphical abstract

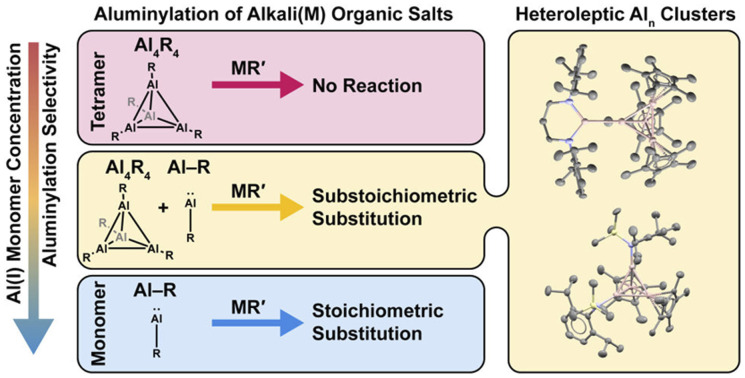

## Introduction

**Fig. 1:**
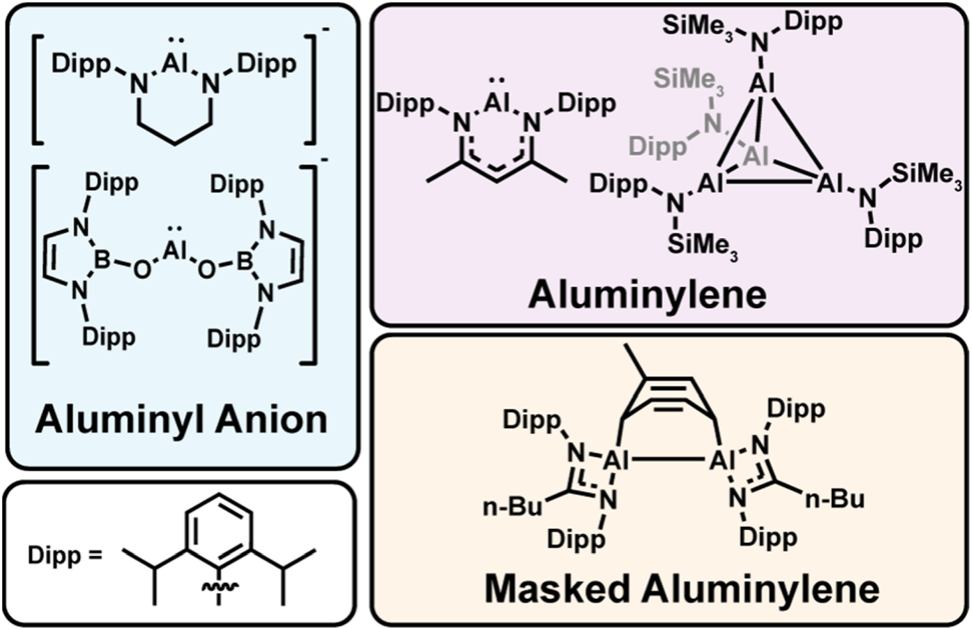
Selection of low-valent aluminum complexes studied in this work, and their classification. Top left – anionic Al(i) complexes bearing two anionic ligands or a single dianionic ligand are defined as aluminyl anions. Top right – neutral Al(i) complexes bearing one anionic ligand are defined as aluminylenes. Bottom right – aluminylenes reacted through the cycloaddition of an aromatic solvent are defined as ‘masked’ aluminylenes.

Low-valent aluminum complexes have emerged as a versatile class of main-group compounds capable of mediating transformations traditionally reserved for expensive, toxic transition metals or strong alkali metal reductants.^1–7^ As the field expands, chemical diversification is clarifying structure–function relationships that push the limits of aluminum-based redox chemistry. In particular, Al(i)-containing complexes, termed “*aluminyls*,” can span a range of electronic environments defined by the supporting ligand, from neutral *aluminylenes*^8–18^ to anionic *aluminyl anions*.^12,19–30^ (Fig. 1). This distinction is critical, as charge and coordination number strongly influence aggregation, redox behavior, and nucleophilicity of Al(i) centers.^12,25,31^ These aluminyl complexes exhibit rich redox behavior, tunable electronic properties, and the ability to engage in small-molecule activation and electron-transfer chemistry.^1,3,4,7,32–37^ Strong σ-donation and the polarizable aluminum center confer a unique balance of nucleophilicity and reducing ability, positioning low-valent aluminum complexes as promising next-generation reductants for selective transformations.

Despite this potential, access to low-valent aluminum species remains synthetically challenging as aluminum most commonly exists in the +3 oxidation state.^38^ Thus, synthetic routes to aluminyl complexes mostly rely on reductive salt elimination from Al(iii) halide precursors, typically with harsh alkali metal reductants. However, the disparity between the extreme reduction potentials of these reagents and the intrinsic redox properties of the aluminum complexes often leads to poor yields.^11,16,17,27,39–45^ Low-valent aluminum is prone to forming metal clusters that bridge the size regimes between distinct molecules and nanoparticle precipitates as potential off-target products.^46–57^ These competing factors define a complex synthetic landscape where side reactions, ligand degradation, or formation of metallic aluminum compete with the desired chemistry, highlighting the need for milder, more controlled synthetic routes.

Aluminylation represents a promising alternative synthetic pathway, as first reported by Aldridge and coworkers.^28–30,58,59^ In this redox neutral reaction, an Al(i) center is transferred through a metathesis-type reaction, avoiding uncontrolled electron transfer, metal deposition, and heterogeneity inherent to reductive chemistry. This approach is enabled by scalable conversion of Cp*_2_AlH (Cp* = pentamethylcyclopentadienyl) to the tetramer [AlCp*]_4_,^13^ a known competent aluminylation reagent.^30,58^ This reaction occurs *via* reductive elimination of Cp*H, avoiding the use of external reductants at any stage. Importantly, the Cp^*^ ligand can be readily exchanged with other Cp (Cp = cyclopentadienyl) derivatives to access new potential aluminylation reagents.^59,60^ In particular, the equilibrium between the tetrameric Al_4_ cluster and the monomeric Al(i) species is known to depend on the Cp ligand electronic and steric profile, directly influencing their solution state chemistry and providing a chemical handle that enables synthetic optimization.

Herein, we describe the divergent chemistry of different aluminylation reagents with a range of nitrogen and oxygen based substrates. The equilibrium between active monomeric and tetrameric aluminyl species in solution is found to provide a synthetic handle for accessing heteroleptic aluminum clusters. In comparison, by increasing the proportion of Al(i) monomers in solution, the yields of known reactions are improved, even under mild conditions. We demonstrate that Al(i) speciation, not only substrate identity, governs reaction outcomes to enable access to aluminyl species with selective nuclearities.

## Results and discussion

### Substrate-directed aluminylation pathways: stoichiometric *vs.* substoichiometric substitution

The equilibrium between tetrameric [AlCp*]_4_ and monomeric AlCp* is central to aluminylation reactivity. With only trace monomer concentrations at room temperature, elevated temperatures are required to shift this equilibrium.^59,61^ Such behavior rationalizes why heating is typically required in Cp*-based aluminylation chemistry, supporting monomeric AlCp* as the active aluminylation species rather than [AlCp*]_4_.^11,29,30^ In looking to expand this chemistry, we noted that bulkier Cp derivatives are known to give Al(i) complexes in which the tetramer–monomer equilibrium is shifted further to the monomer product. More generally, ligand bulk determines aluminyl nuclearity. Exceptionally bulky ligands can stabilize monomeric aluminyl species,^9,26,59,62,63^ whereas reduced steric demand typically promotes aggregation to aluminum dimers,^41,43^ trimers,^64^ or tetramers.^13,56,59,65,66^ These observations suggest that both the substrate ligand framework and the aluminylation reagent itself can be used as a powerful handle for tuning the outcome of aluminylation chemistry across ligand classes.

To broaden aluminylation scope, we compared aluminylation products with substrates known to stabilize aluminyl molecules with different nuclearities across a range of steric profiles. We targeted both “masked” low-valent aluminum(i) amidinate dimers and asymmetric amide tetrameric clusters (where “masked” refers to cycloaddition of an aromatic solvent to an aluminylene fragment). In this context, amide and amidinate ligands offer a more flexible and tunable alternative to the commonly explored Cp frameworks. Such chemically diversifiable complexes provide a versatile platform for systematic studies of aluminylene reactivity,^64^ motivating the development of more direct synthetic routes away from reductive salt elimination.

**Scheme 1:**
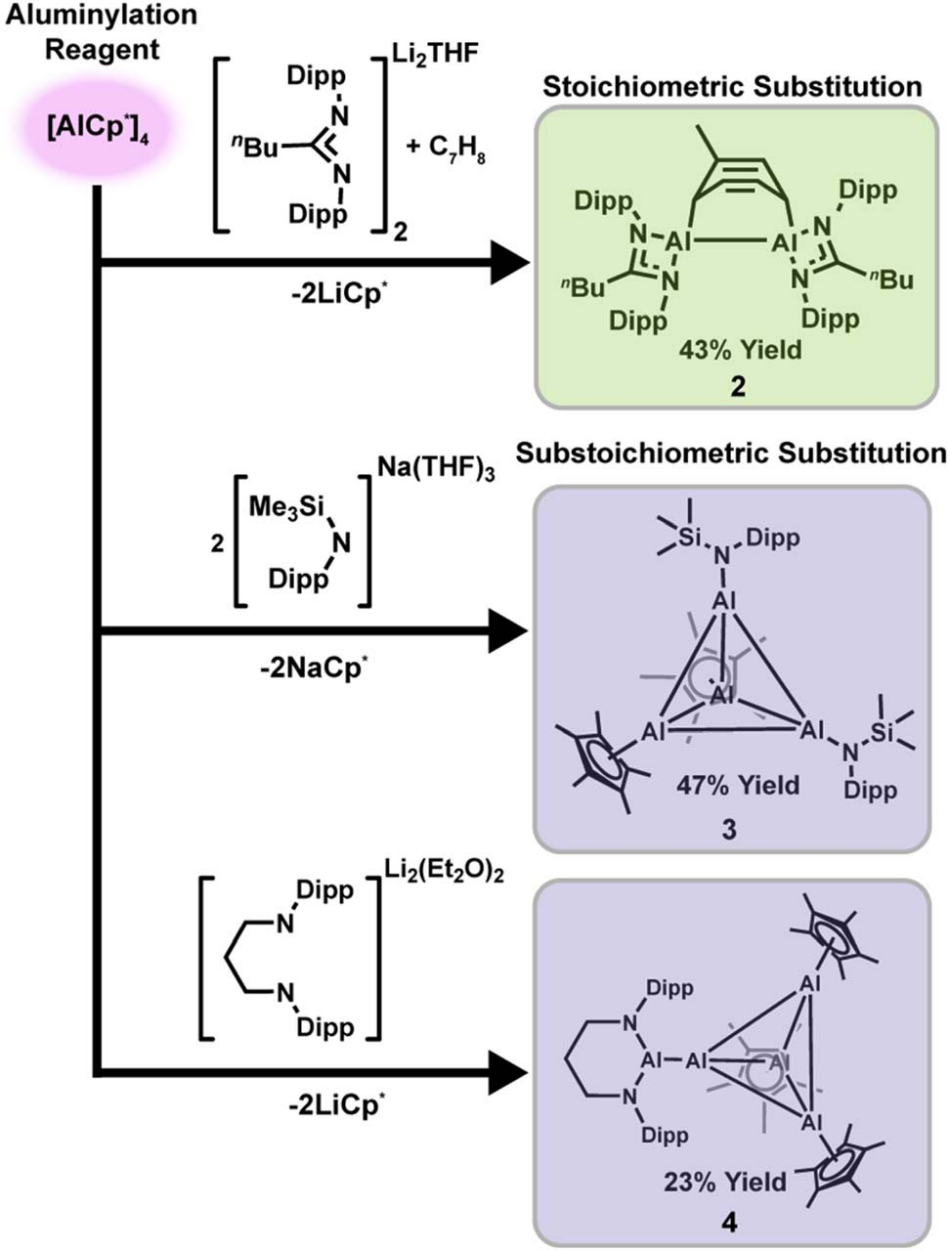
Aluminylation of various ligand scaffolds using [AlCp*]_4_ at elevated temperature to access the active monomer AlCp*. Top – aluminylation of amidinate ligand selects for stoichiometric substitution in the formation of [(Al(NDipp)_2_C_5_H_9_)_2_C_7_H_8_]·C_7_H_8_ (2). Middle – aluminylation of amide leads to substoichiometric substitution in the formation of heteroleptic cluster [Cp*AlAlN(SiMe_3_)(Dipp)]_2_·C_6_H_14_ (3). Bottom – aluminylation of dianionic bisamide leads to aluminyl anion appended to the [Al_4_Cp*_3_]^+^ cluster as [Al(N_2_Dipp_2_C_3_H_6_)(Al_4_Cp*_3_)]·0.5C_6_H_6_ (4).

Reaction of the lithium amidinate salt [(Li(NDipp)_2_C_5_H_9_)_2_·THF] (1; THF = tetrahydrofuran, Dipp = 2,6-diisopropylphenyl) with [AlCp^*^]_4_ in toluene at 60 °C for 36 h afforded the masked aluminylene dimer [(Al(NDipp)_2_C_5_H_9_)_2_C_7_H_8_]·C_7_H_8_ (2) in 43% isolated yield (Scheme 1) ,top, comparable to reported reductive preparations. In contrast, aluminylation of the less hindered amide system gave divergent behavior. Treatment of Na[N(SiMe_3_)(Dipp)]·3 Et_2_O with [AlCp*]_4_ (4 : 1 molar ratio) at 60 °C for 12 h afforded the substoichiometric heteroleptic cluster [Cp*AlAlN(SiMe_3_)(Dipp)]_2_·C_6_H_14_ (3) in 47% isolated yield (Scheme 1), middle. This product is a rare example of a heteroleptic aluminum cluster, and we do not observe evidence of either the fully substituted cluster, or other substoichiometric products, even by ^27^Al NMR spectroscopy. The ^27^Al resonances of 3 also do not change in relative intensity upon heating (see Fig. S6), consistent with the absence of a monomer–tetramer equilibrium. This suggests that AlCp* monomer is responsible for aluminyl transfer, but subsequent Al–Al aggregation of Al(i) monomers yields a heteroleptic cluster 3 that does not participate in a monomer–tetramer equilibrium.

We next targeted aluminyl anions supported by dianionic ligands, which are typically prepared *via* a two-step reductive salt-elimination route that requires demanding purifications and often delivers lower yields. A one-step aluminylation reaction offers an appealing alternative, and so we aimed to access the previously reported aluminyl anion [Al(NDipp)_2_(CH_2_)_3_]^−^ directly.^26^ Accordingly, we treated Li_2_[(NDipp)_2_(CH_2_)_3_]·2 Et_2_O with [AlCp^*^]_4_ (4 : 1 molar ratio) at 60 °C. The expected aluminylation product was not observed; instead, the reaction produced a blue cluster, [Al(N_2_Dipp_2_C_3_H_6_)(Al_4_Cp^*^_3_)]·0.5C_6_H_6_ (4), in <10% yield. Adjusting the ratio (Li_2_[(NDipp)_2_(CH_2_)_3_]·2Et_2_O : [AlCp^*^]_4_ = 4 : 5) increased the isolated yield to 23% (Scheme 1), bottom. Complex 4 can be described as [Al(NDipp)_2_(CH_2_)_3_]^−^ associated with an [Al_4_Cp*_3_]^+^ fragment. We propose that the aluminyl anion Li[Al(NDipp)_2_(CH_2_)_3_] forms *in situ* and undergoes salt metathesis with [AlCp*]_4_ present in solution, although no intermediates were detected. Such a mechanism suggests that both monomeric AlCp* and tetrameric [AlCp*]_4_ can be reactive in the presence of sufficiently nucleophilic anions.

This potential mechanism is mirrored in the cluster connectivity, as this molecule presents, to the best of our knowledge, the first example of an Al cluster that does not conform to standard polyhedral skeletal electron-pair descriptions of these metal clusters. Instead, this molecule exhibits two chemically distinct aluminyl environments connected *via* a direct Al–Al bond. Compared with the heteroleptic tetramer 3, this substrate-dependent aluminylation behavior underscores the potential of clustered aluminylation reagents as a deliberate strategy for accessing heteroleptic, low-valent aluminyl architectures. These structures provide a foundation for targeting site-selective reactivity at differentiated aluminum centers, which will be the focus of future studies.

### Crystallographic analysis of heteroleptic aluminum clusters

From a structural perspective, these new molecules also enable unusual comparisons to typical aluminum clusters with electron density delocalized isotropically over a homoleptic metal framework. In such cases, Al–Al bond distances provide a practical proxy for relative bond strength. For example, the average Al–Al separation in [AlCp*]_4_ is 2.769(7) Å,^67^ whereas [AlN(SiMe_3_)(Dipp)]_4_ exhibits a shorter average distance of 2.62(13) Å.^11^ This contraction is consistent with stronger Al–Al interactions in the amide system and rationalizes why monomer–tetramer equilibria are more pronounced for [AlCp^*^]_4_ than for [AlN(SiMe_3_)(Dipp)]_4_.

**Fig. 2:**
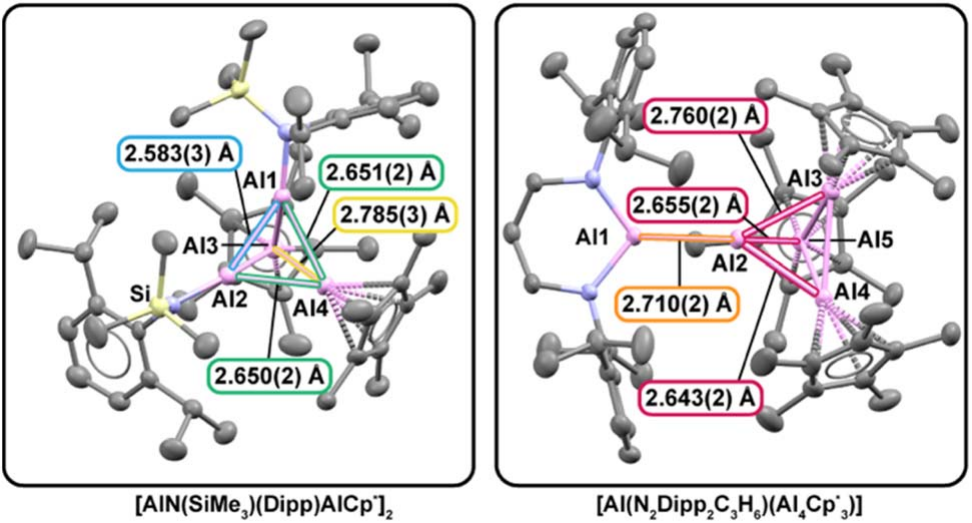
Solid-state molecular structures of the heteroleptic aluminum clusters [CpAlAlN(SiMe_3_)(Dipp)]_2_ (left; 3) and [Al(N_2_Dipp_2_C_3_H_6_)(Al_4_Cp*_3_)] (right; 4). Selected Al–Al bond lengths are shown to highlight ligand-dependent variations in Al–Al interactions and polarization within the clusters. Hydrogen atoms and outer sphere solvents are omitted for clarity.

In contrast, the heteroleptic clusters 3 and 4 are intrinsically less symmetric; therefore, electron density is expected to be polarized in the heteroleptic Al–Al bonds (Fig. 2). We note that in 3, its heteroleptic stoichiometry enforces three distinct classes of Al–Al interactions and distorts the framework away from ideal tetrahedral geometry of an Al_4_ core. The Cp*Al–AlCp* distances (2.785(3) Å) are close to those in [AlCp*]_4_ (2.769(7) Å), while the (Dipp)(SiMe_3_)NAl–AlN(SiMe_3_)(Dipp) distance (2.583(3) Å) tracks the shorter separations characteristic of the homoleptic amide cluster [AlN(SiMe_3_)(Dipp)]_4_ (2.62(13) Å). The mixed Cp*Al–AlN(SiMe_3_)(Dipp) contacts fall between these limits. Collectively, these metrics indicate that N(SiMe_3_)(Dipp) exerts a stronger influence on Al–Al bond contraction than Cp*, mirroring trends in the homoleptic analogues. The resulting asymmetric bonding environment affects both local bonding and global cluster shape, yielding a geometry more consistent with a nido-type arrangement than the idealized closo geometry expected from simple electron-counting arguments.^68,69^

In comparison, complex 4 represents an even more significant departure away from idealized cluster geometry. The Al_5_ framework of Al(i) centers in 4 corresponds formally to an overall oxidation-state sum of +5. An even distribution of charge across the structure would treat the Al–Al_4_ bonding interaction as predominantly ionic. Alternatively, a covalent description of Al–Al bonding invokes an uneven oxidation-state distribution across the Al_5_ unit (*e.g.*, R_2_Al(ii)–Al(0)Al(i)_3_). Either formulation departs from the high symmetries exhibited by aluminum cluster precedents and points to significantly asymmetric and polarized Al–Al interactions.

X-ray diffraction studies on single crystals of 4 revealed the interfragment Al–Al_4_ linkage is 2.710(2) Å, longer than the sum of metallic radii for two Al atoms (2.50 Å) and other typical covalent metrics,^70^ yet shorter than an estimate for a purely ionic contact between [Al(N_2_Dipp_2_C_3_H_6_)]^−^ and [Al_4_Cp*_3_]^+^ (2.82 Å; see SI for how the ionic-limit estimate is derived for these fragments). These comparisons place the interaction between covalent and ionic extremes, consistent with a mixed ionic–covalent contact. Together, these crystallographic results show how ligand identity and aluminylation pathway directly govern Al–Al bond character and cluster geometry, providing access to heteroleptic, low-valent aluminum frameworks not readily available from reductive synthesis pathways where redox chemistry competes with equilibria defined by all Al(i) species present in solution.

### DFT, QTAIM, and ELF analysis of Al–Al bonding in 3 and 4

To further probe the Al–Al bonding in these clusters, we carried out density functional theory (DFT) calculations on truncated structural models (*e.g.*, Cp* → Cp),^71^ with geometries constrained to match solid-state structures. Calculations employed the B3LYP functional with the def2-SVP basis set and the def2/J auxiliary basis.^72–74^ The model of 3 converged on a true minimum while the model of 4 converged on a low-energy saddle point (see SI for further details).

For the model of 3, Kohn–Sham orbital analysis shows that the HOMO through HOMO−2 are dominated by the antisymmetric Al–Al bonding combinations characteristic of Al_4_ tetramers, while deeper orbitals (*e.g.*, HOMO−23 and HOMO−31) correspond to fully symmetric Al_4_ bonding combinations. The low-lying virtual space mirrors this pattern: the LUMO through LUMO+2 predominantly comprise the antisymmetric antibonding combinations of the Al_4_ framework. Qualitatively, these frontier-orbital features are consistent with other Al(i) tetramer clusters; however, real-space analyses reveal key differences in bonding polarization within the heteroleptic core.

Quantum Theory of Atoms in Molecules (QTAIM)^75,76^ analysis (see SI, Section 3) differentiates the Al–Al interactions within 3 by quantifying electron density and its topology at the bond critical points (BCPs). The electron density (*ρ*(*r*)) at the Al–Al BCPs is low relative to typical covalent bonds (often *ρ*(*r*) > 0.1 *e*·Bohr^−3^, *cf. ρ*(*r*) = 0.0356–0.0465 *e*·Bohr^−3^), but the Laplacian is negative (∇^2^*ρ*(*r*) < 0), indicating charge concentration consistent with a covalent interaction (Table 1). At the same time, standard ionic descriptors (*e.g.*, Δ*G*(*r*)/*ρ*(r) > 1; Δ*G*(*r*) = kinetic energy density and |Δ*V*(*r*)|/Δ*G*(*r*) < 1; Δ*V*(*r*) = potential energy density) do not support a purely ionic description. This intermediate regime is consistent with trends reported for other low-valent aluminum clusters.

**Table 1:** Real-space descriptors at bond critical points (BCPs) for 3 and 4

	Bond length^a^, Exp./Calc.	Electron density *ρ*(r)^b^	Laplacian of electron density, ∇^2^*ρ*(r)^b^	Ellipticity, *ε*
**Structure 3**				
CpAl3-Al4Cp	2.785(2)/2.754	0.036	−0.010	1.387
NAl1–Al2N	2.583(3)/2.582	0.045	−0.041	0.572
CpAl1-Al4N	2.650(2)/2.578	0.047	−0.026	0.775
CpAl2-Al4N	2.651(3)/2.669	0.041	−0.027	0.778

**Structure 4**				
Al1–Al_4_	2.710(2)/2.710	0.048	−0.034	0.030
AlAl2–Al4Cp	2.643(2)/2.600	0.046	−0.033	0.630
AlAl2–Al5Cp	2.660(2)/2.632	0.044	−0.037	0.537
AlAl2–Al3Cp	2.655(2)/2.601	0.046	−0.034	0.637
CpAl3-Al4Cp	2.721(2)/2.671	0.039	−0.025	1.268
CpAl4-Al5Cp	2.722(2)/2.717	0.037	−0.016	0.850
CpAl3-Al5Cp	2.694(2)/2.668	0.040	−0.025	0.844

a Unit length Å.

b Units for *ρ*(r) and ∇^2^*ρ*(r) in a.u. (*e*·Bohr^−3^, and *e*·Bohr^−5^ respectively).

Electron Localization Function (ELF)^77,78^ analysis further indicates both that the bonding density in 3 is displaced from the internuclear Al–Al axis and polarized along the bond. The ellipticity (*ε*) for each Al–Al bond is greater than 0.5, suggesting the electron density is biased toward the exterior of the cluster, whereas the BCP remains close to the internuclear line, near the boundary of the ELF-delineated bonding region (see Fig. S67–S72). Relative to homoleptic analogues, 3 also shows a systematic shift of the BCP position toward the amide-substituted vertices, concomitant with smaller *ε* values (0.572 *vs.* 1.387). This trend is consistent with stronger σ-donor ligands increasing σ-character in the Al–Al interactions *via* inductive polarization. Overall, these descriptors indicate that ligand σ-donation measurably strengthens the Al–Al bond, as reflected in lower *ε* values concomitant with larger values of *ρ*(*r*) and |∇^2^*ρ*(*r*)| at the BCP of the Al–Al bonds supported by amide ligands compared with those supported by Cp ligands, matching the observed bond distance metrics. These electronic effects install stability into the Al–Al bonds that define the Al_*n*_^*n*+^ framework, stabilizing higher-nuclearity clusters by preventing disproportionation to entropically favored lower nuclearity motifs. This phenomenon prevents the rearrangements of the cluster fragments present in 3 to other stoichiometries and indicates a path towards designing heteroleptic systems based on electronic structure requirements in addition to steric constraints.

For 4, we anticipated analogous polarization effects within the Al_4_ subunit, together with unique bonding at the interfragment Al–Al_4_ linkage. Accordingly, the HOMO is localized at the Al–Al_4_ and exhibits substantial lone-pair character localized at the formally anionic Al center, whereas lower-energy orbitals (notably HOMO−10 and HOMO−15) contain clear σ-bonding contributions associated with the Al–Al_4_ interaction. The LUMO and LUMO+1 are dominated by π_*x*_/π_*y*_-type bonding combinations between the Al and Al_4_ fragments (see Fig. S53 and S54), identifying the Al–Al_4_ linkage as a key frontier-orbital site for reactivity.

**Fig. 3:**
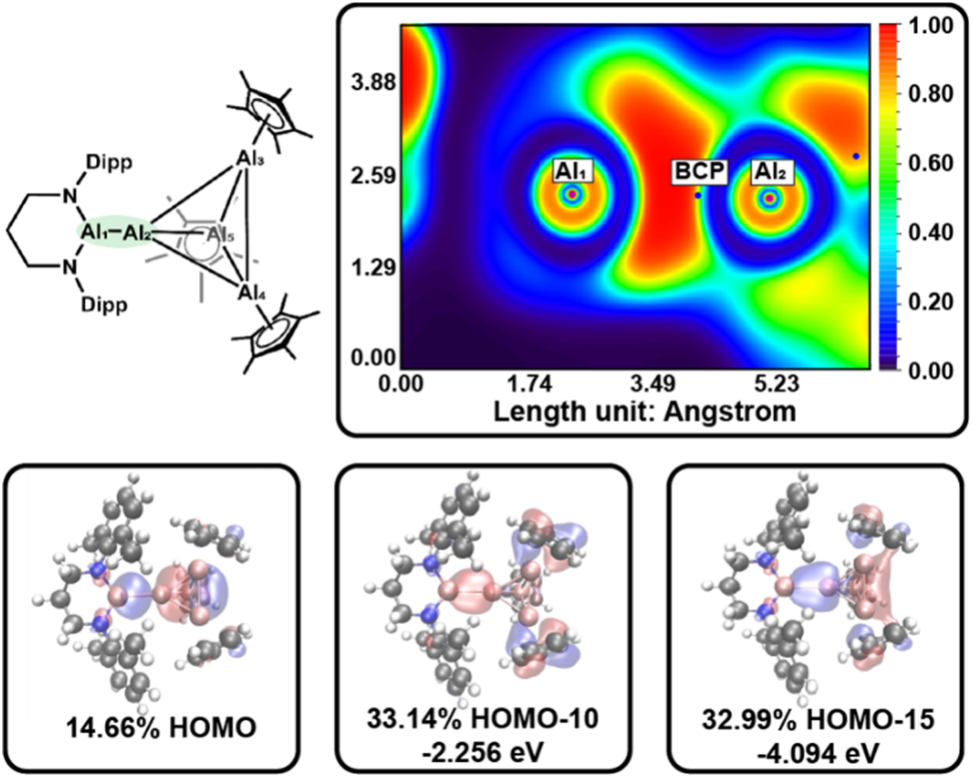
Top – ELF plot in the plane containing Al1, Al2, and the bond critical point (BCP) between them. Bottom – decomposition of the electron density at the BCP into contributions from the three most significant molecular orbitals (MOs).

QTAIM analysis of 4 identifies polarized BCPs along the AlAl–AlCp contacts in the Al_4_ core toward the aluminyl anion containing vertex. This polarization, similar to that observed in 3, indicates that the aluminyl anion exerts a stronger σ-donating (and inductively polarizing) influence on the Al_4_ framework than the Cp ligand. Similarly, *ε* of the Al–Al contacts in Al_4_ core shows significant bias of electron density to the exterior of the Al_4_ subunit (*ε* = 0.537–1.268). In comparison, the BCP located along the interfragment Al–Al_4_ bond path exhibits a relatively low *ρ*(r) and a negative Laplacian, consistent with a weak shared interaction (Table 1). Unlike the Al–Al bonds within the Al_4_ core, where the ELF density is displaced from the internuclear axis, ELF analysis shows that the Al–Al_4_ interaction is nearly collinear, with the BCP positioned close to the internuclear line and a low value of *ε* = 0.030. The BCP lies at the boundary of the ELF-defined bonding region on the Al_4_ side, consistent with a strongly polarized interaction in which electron density is drawn toward the aluminyl fragment (Fig. 3). Analysis of the Al–Al_4_ disynaptic basin containing 2.033 electrons, shows this electron density partitioned unevenly between the aluminyl anion center (1.479 *e*) and the Al_4_ fragment (0.512 *e*). Together, the MO, QTAIM, and ELF results support describing this Al–Al_4_ linkage as a weakly covalent bond with pronounced polarization toward the aluminyl anion fragment. This homonuclear interaction is electronically heterogenous, with electron density being favorably localized on the aluminyl anion with only a small contribution of electron density being shared between the Al centers leading to the weak covalent metrics observed in the QTAIM. Other metal homonuclear M–M interactions do not commonly express this electronic heterogeneity, regardless of ligand differences,^79^ making this polarized interaction tractable for further studies.

### Suppressing cluster formation: leveraging monomeric AlCp^*i*Pr4^ for milder aluminylation

**Scheme 2:**
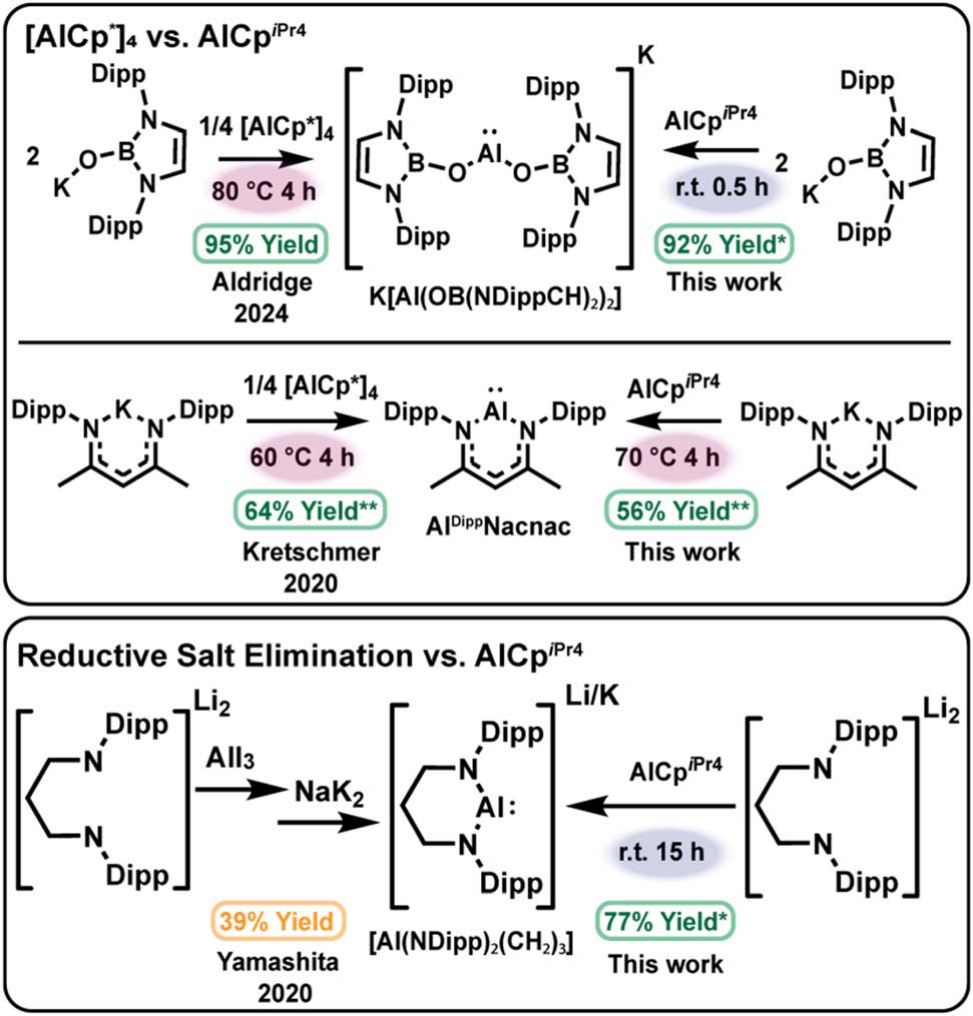
Comparison of aluminylation reagents [AlCp*]_4_ (top left) and AlCp^*i*Pr4^ (top right) in the synthesis of aluminyl anion K[Al(OB(NDippCH)_2_)_2_] and aluminylene Al^Dipp^Nacnac. Conditions highlight the thermal requirements that are necessary for reactivity with [AlCp*]_4_ while not necessarily required for AlCp^*i*Pr4^. Use of mononuclear AlCp^*i*Pr4^ in the aluminylation of dianionic salt Li_2_[(NDipp)_2_(CH_2_)_3_]·(Et_2_O)_2_ yields the aluminyl anion Li[Al(NDipp)_2_(CH_2_)_3_] analogous to the anion obtained through reductive elimination routes in one step higher yielding process (bottom). *In situ yield; **in situ yield/% consumed aluminylation reagent.

This cluster chemistry also underscores an additional requirement for selective aluminylation: as the tetramer–monomer equilibrium is instrumental for the formation of heteroleptic clusters, stoichiometric products may be favored by shifting the equilibrium of the aluminylation reagent toward monomeric species. We identified AlCp^*i*Pr4^, a known monomer in solution at room temperature,^59^ as a potential aluminylation candidate as opposed to the thermally accessed AlCp* monomer. Beyond this, at −40 °C AlCp^*i*Pr4^ is monomeric, as determined by diagnostic shifts in the ^27^Al NMR spectra in our experiments. Thus, we predicted that AlCp^*i*Pr4^ could perform aluminylation chemistry without the entropic penalty associated with tetramer dissociation, supporting mild reaction conditions and enabling direct comparisons of the temperature-dependent reactivity for AlCp^*i*Pr4^*versus* [AlCp*]_4_ (Scheme 2).

A practical advantage of aluminylation over heterogeneous reductions is its compatibility with *in situ* NMR monitoring (see SI). Reactions were performed in sealed NMR tubes with an internal standard. Here, *in situ* yield is defined as the net increase in product concentration divided by the net decrease in the concentration of the limiting reagent. Where thermal decomposition complicates time-dependent yields (as similarly reported by Kretschmer and co-workers),^58^ values are normalized to the fraction of aluminylation reagent consumed at early reaction times to enable meaningful comparisons.

We tested two literature aluminylations with contrasting outcomes as a benchmark of this approach: clean formation of [Al(OB(NDippCH)_2_)_2_]^−30^ and the more side-reaction-prone formation of Al^Dipp^Nacnac.^58^ Relative to AlCp* generated from [AlCp*]_4_ at 60–80 °C, AlCp^*i*Pr4^ delivered K[Al(OB(NDippCH)_2_)_2_] rapidly at room temperature (92% *in situ* yield in 30 min; see SI). In comparison, no reaction with K^Dipp^Nacnac was observed at room temperature, instead heating to 70 °C was required to form Al^Dipp^Nacnac, indicating that temperature dependence can reflect substrate-specific activation barriers as well as precursor speciation (Scheme 2), top. Normalized early-time yields under heating were similar for AlCp* and AlCp^*i*Pr4^ in the synthesis of Al^Dipp^Nacnac (64% *vs.* 56%). Furthermore, reaction of the lithium amidinate salt with AlCp^*i*Pr4^ in C_6_D_6_ at room temperature proceeded slowly (40 days) but ultimately furnished the masked dimer [(Al(NDipp)_2_C_5_H_9_)_2_C_6_D_6_]·C_6_H_14_ in an improved isolated yield of ∼60% compared to that of [AlCp^*^]_4_, demonstrating that aluminylation can be achieved at ambient temperature with a predominantly monomeric Cp reagent.

We also sought to compare the effect of a monomeric aluminylation reagent on the cluster formation described above. At room temperature, reaction of (THF)_3_Na[N(SiMe_3_)(Dipp)] with AlCp^*i*Pr4^ over 36 h furnished the known tetramer [AlN(SiMe_3_)(Dipp)]_4_ in 66% crude yield.^11^ In a similar fashion, treatment of Li_2_[(NDipp)_2_(CH_2_)_3_]·(Et_2_O)_2_ with AlCp^*i*Pr4^ (1 : 1) at room temperature for 15 h produced the aluminyl anion Li[Al(NDipp)_2_(CH_2_)_3_] in 77% *in situ* yield (Scheme 2), bottom, with comparable ^1^H NMR spectrum to the potassium analogue K[Al(NDipp)_2_(CH_2_)_3_] (Table S1).^26^ Together, these results demonstrate that aluminylation provides a tractable route to tune low-valent aluminum nuclearity through deliberate control of Al(i) reagent speciation and ligand environment.

## Conclusions

This work establishes aluminylation as a general, redox-neutral strategy for accessing low-valent aluminum complexes across diverse ligand classes. However, the nuclearity and speciation of the Al(i) transfer reagent can govern product identity as strongly as substrate choice. In particular, [AlCp*]_4_ is not merely an Al(i) source: its monomer–tetramer equilibrium can divert reactivity toward heteroleptic Al–Al cluster motifs that are difficult to access using monomeric reagents or traditional reductive routes. Exploiting this dynamic behavior provides a platform for constructing structurally and electronically diverse low-valent aluminum clusters, expanding opportunities to tune aggregation, bonding polarity, and ultimately reactivity in aluminyl chemistry. In comparison, AlCp^*i*Pr4^ functions as an effective aluminylation reagent under mild conditions, enabled by its persistent monomeric speciation at lower temperatures. Together, these results reveal a path toward enabling controlled Al(i) transfer and access to increasingly reactive low-valent aluminum species.

## Author contributions

P. C. R. was responsible for the majority of the investigation, including data curation, formal analysis and writing the original draft. Y. S. helped with the investigation of aluminyl anions and M. T. W. provided resources in the form of synthesized ligands. A. B. A was responsible for conceptualization, funding acquisition, project administration, supervision of other researchers as well as writing-review and editing responsibilities.

## Conflicts of interest

There are no conflicts to declare.

## Supplementary Material

SC-017-D6SC02187E-s001

SC-017-D6SC02187E-s002

## Data Availability

CCDC 2531049–2531053 contain the supplementary crystallographic data for this paper.^80*a*–*e*^ The data supporting this article has been included as part of the supplementary information (SI). Supplementary information: full experimental procedures for synthesis of all compounds, characterization details and computational modeling results (^1^H, ^13^C, ^27^Al NMR spectra, crystallographic parameters, Fourier Transform Infrared spectra, UV-vis spectra, Molecular Orbital isosurfaces, Electron Localization Function plots, Tables of Quantum Theory of Atoms in Molecules Critical Points and Density Functional Theory calculated coordinates). See DOI: https://doi.org/10.1039/d6sc02187e.
