# Supplementary material for: Aluminylation: a generalizable route towards low-valent aluminum under moderate conditions with controlled product nuclearity through precursor design
Source: Chem Sci. 2026 Apr 13;17(20):10251–9. doi: 10.1039/d6sc02187e (PMC13075507; doi:10.1039/d6sc02187e)
Supplement: SC-017-D6SC02187E-s001 [file SC-017-D6SC02187E-s001.pdf]

Supplementary Information (SI) for Chemical Science.
This journal is © The Royal Society of Chemistry 2026

Supplementary Information (SI)

Aluminylation: a generalizable route towards low-valent aluminum under moderate conditions with controlled product nuclearity through precursor design

Paris C. Reuel,[a] Yogesh Shandilya,[a] M. Talha Wattoo[a] and Alison B. Altman[a]*

a. *Department of Chemistry, Texas A&M University, College Station, TX, 77843, United States.*

*E-mail address: aaltman@tamu.edu

Table of Contents

1. Experimental ... 7

1.1.1 General Considerations ... 7

1.1.2 Materials and Methods ... 8

1.2.1 Synthesis of NaCp^{iPr_4} ... 8

1.2.1.1 Triisopropylcyclopentadiene (HCp^{iPr_3}) ... 8

1.2.1.2 Tetraisopropylcyclopentadiene (HCp^{iPr4}) ... 9

1.2.1.3 Sodium Tetraisopropylcyclopentadienide (NaCp^{iPr4}) ... 9

1.2.2 Synthesis of AlCp^{iPr4} ... 9

1.2.3 Synthesis of [(Li(NDipp)$_2$C$_5$H$_9$)$_2$THF] ... 10

1.2.6 Synthesis of [(Al(NDipp)$_2$C$_5$H$_9$)$_2$C$_7$H$_8$]·C$_7$H$_8$ ... 10

1.2.10 Synthesis of [AlN(SiMe$_3$)DippAlCp*]$_2$·C$_6$H$_{14}$ ... 12

1.2.8 Synthesis of [Al(N$_2$Dipp$_2$C$_3$H$_6$)(Al$_4$Cp*$_3$)]·0.5C$_6$H$_6$ ... 13

1.2.4 Synthesis of K[Al(OB(NDippCH)$_2$)$_2$] ... 14

1.2.4.1 Method A – [AlCp*]$_4$ ... 14

1.2.4.2 Method B – AlCp^{iPr4} ... 14

1.2.5 Synthesis of Al^{Dipp}Nacnac ... 14

1.2.5.1 Method A – [AlCp*]$_4$ ... 14

1.2.5.2 Method B – AlCp^{iPr4} ... 15

1.2.7 Synthesis of [(Al(NDipp)$_2$C$_5$H$_9$)$_2$C$_6$D$_6$]·C$_6$H$_{14}$ ... 16

1.2.11 Synthesis of [AlN(SiMe$_3$)(Dipp)]$_4$ ... 17

1.2.9 Synthesis of [LiAl(NDipp)$_2$(CH$_2$)$_3$] ... 17

2 Calculation of the effective radius of fragments in [Al(N$_2$Dipp$_2$C$_3$H$_6$)(Al$_4$Cp*$_3$)] ... 19

2.1 Empirical estimate to the effective ionic radius of [Al(N$_2$Dipp$_2$C$_3$H$_6$)]$^-$ ... 19

2.2 Empirical estimate to the effective ionic radius of [Al$_4$Cp*$_3$]$^+$ ... 19

3 DFT, QTAIM, ELF ... 20

3.1 Calculation details ... 20

3.1.1 Density functional theory (DFT) ... 20

3.1.2 Quantum theory of atoms in molecules (QTAIM) and electron localization function (ELF) ... 20

4 NMR Data: ... 22

Figure S1. ^{27}Al NMR of **[AlCp*]$_4$** at room temperature. ... 22

Figure S2. ^{27}Al NMR of **[AlCp*]$_4$** at 65 °C ... 22

Figure S3. ^{27}Al NMR of **AlCp^{iPr4}** at room temperature ... 23

Figure S4. ^{27}Al NMR of **AlCp^{iPr4}** at –40 °C 23
Figure S5. ^{27}Al NMR of **[AlNTMSDippAlCp*]$_2$·C$_6$H$_{14}$** at room temperature 24
Figure S6. ^{27}Al NMR of **[AlNTMSDippAlCp*]$_2$·C$_6$H$_{14}$** at 65 °C 24
Figure S7. ^{27}Al NMR of **[Al(N$_2$Dipp$_2$C$_3$H$_6$)(Al$_4$Cp*$_3$)]·0.5C$_6$H$_6$** at room temperature 25
Figure S8. ^{1}H NMR spectra of **[(Li(NDipp)$_2$C$_5$H$_9$)$_2$THF]**. 26
Figure S9. ^{1}H NMR spectra of **[(Al(NDipp)$_2$C$_5$H$_9$)$_2$C$_7$H$_8$]·C$_7$H$_8$**.. 27
Figure S10. ^{1}H NMR spectra of **[AlNTMSDippAlCp*]$_2$·C$_6$H$_{14}$**. 28
Figure S11. ^{1}H NMR spectra of **[Al(N$_2$Dipp$_2$C$_3$H$_6$)(Al$_4$Cp*$_3$)]·0.5C$_6$H$_6$**. 29
Figure S12. ^{1}H NMR spectra of **K[Al(OB(NDippCH)$_2$)$_2$]**. 30
Figure S13. ^{1}H NMR spectra of **Al^{Dipp}Nacnac**. 31
Figure S14. ^{1}H NMR spectra of **[(Al(NDipp)$_2$C$_5$H$_9$)$_2$C$_6$D$_6$]·C$_6$H$_{14}$**. 32
Figure S15. ^{1}H NMR spectra of **[AlN(SiMe$_3$)(Dipp)]$_4$**. 33
Figure S16. HSQC 2-D correlation spectra of **AlCp^{iPr4}**. 34
Figure S17. HMBC 2-D correlation spectra of **AlCp^{iPr4}**. 35
Figure S18. HSQC 2-D correlation spectra of **[(Li(NDipp)$_2$C$_5$H$_9$)$_2$THF]** 36
Figure S19. HMBC 2-D correlation spectra of **[(Li(NDipp)$_2$C$_5$H$_9$)$_2$THF]**. 37
Figure S20. HSQC 2-D correlation spectra of **[(Al(NDipp)$_2$C$_5$H$_9$)$_2$C$_7$H$_8$]·C$_7$H$_8$**. 38
Figure S21. HSQC 2-D correlation spectra of **[(Al(NDipp)$_2$C$_5$H$_9$)$_2$C$_6$D$_6$]·C$_6$H$_{14}$**. 39
Figure S22. HMBC 2-D correlation spectra of **[(Al(NDipp)$_2$C$_5$H$_9$)$_2$C$_6$D$_6$]·C$_6$H$_{14}$**. 40
Figure S23. HSQC 2-D correlation spectra of **[AlNTMSDippAlCp*]$_2$·C$_6$H$_{14}$**. 41
Figure S24. HSQC 2-D correlation spectra of **[Al(N$_2$Dipp$_2$C$_3$H$_6$)(Al$_4$Cp*$_3$)]·0.5C$_6$H$_6$**. 42
Figure S25. HMBC 2-D correlation spectra of **[Al(N$_2$Dipp$_2$C$_3$H$_6$)(Al$_4$Cp*$_3$)]·0.5C$_6$H$_6$**. 43
Figure S26. HSQC 2-D correlation spectra of **[AlN(SiMe$_3$)(Dipp)]$_4$**. 44
Figure S27. *In-situ* ^{1}H NMR spectrum from the synthesis of **K[Al(OB(NDippCH)$_2$)$_2$]**. 45
Figure S28. Deconvoluted ^{1}H NMR signals for the reagents and products of **AlCp*** and **K^{Dipp}Nacnac** to produce **Al^{Dipp}Nacnac**. 46
Figure S29. Deconvoluted ^{1}H NMR signals for the reagents and products of **AlCp^{iPr4}** and **K^{Dipp}Nacnac** to produce **Al^{Dipp}Nacnac**. 47
Figure S30. Deconvoluted ^{1}H NMR signals for the reagents and products of **AlCp^{iPr4}** and **LiAl(NDipp)$_2$(CH$_2$)$_3$** to produce **LiAl(NDipp)$_2$(CH$_2$)$_3$**. 48
5. Single Crystal Structures 49
Figure S31. Solid state structure of **[(Li(NDipp)$_2$C$_5$H$_9$)$_2$THF]** 49
Figure S32. Solid state structure of **[(Al(NDipp)$_2$C$_5$H$_9$)$_2$C$_7$H$_8$]·C$_7$H$_8$** 50
Figure S33. Solid state structure of **[(Al(NDipp)$_2$C$_5$H$_9$)$_2$C$_6$D$_6$]·C$_6$H$_{14}$** 51

Figure S34. Solid state structure of **[AlNTMSDippAlCp*]$_2$·C$_6$H$_{14}$** 52
Figure S35. Solid state structure of **[Al(N$_2$Dipp$_2$C$_3$H$_6$)(Al$_4$Cp$^*_3$)]·0.5C6H6** 53
6. FTIR data 54
Figure S36. FTIR-ATR of **NaCp^{iPr4}** 54
Figure S37. FTIR-ATR of **AlCp^{iPr4}** 54
Figure S38. FTIR-ATR of **[(Li(NDipp)$_2$C$_5$H$_9$)$_2$THF]** 55
Figure S39. FTIR-ATR of **[(Al(NDipp)$_2$C$_5$H$_9$)$_2$C$_7$H$_8$]·C$_7$H$_8$** 55
Figure S40. FTIR-ATR of **[AlNTMSDippAlCp*]$_2$·C$_6$H$_{14}$** 56
Figure S41. FTIR-ATR of **[Al(N$_2$Dipp$_2$C$_3$H$_6$)(Al$_4$Cp$^*_3$)]·0.5C$_6$H$_6$** 56
Figure S42. FTIR-ATR of **[(Al(NDipp)$_2$C$_5$H$_9$)$_2$C$_6$D$_6$]·C$_6$H$_{14}$** 57
7. UV-Vis 58
Figure S43. UV-Vis Spectra of **[AlNTMSDippAlCp*]$_2$·C$_6$H$_{14}$** (**3**) and **[Al(N$_2$Dipp$_2$C$_3$H$_6$)(Al$_4$Cp$^*_3$)]·0.5C$_6$H$_6$** (**4**). 58
8. Molecular orbital (MO) isosurface 59
Figure S44. Model **3** isosurface of MO−93, HOMO-49 (isovalue = -0.03, 0.03). 59
Figure S45. Model **3** isosurface of MO−111 HOMO-31 (isovalue = -0.03, 0.03).. 59
Figure S46. Model **3** isosurface of MO−119 HOMO-23 (isovalue = -0.03, 0.03).. 60
Figure S47. Model **3** isosurface of MO−130 HOMO-12 (isovalue = -0.03, 0.03). 60
Figure S48. Model **3** isosurface of MO−140 HOMO-2 (isovalue = -0.03, 0.03). 61
Figure S49. Model **3** isosurface of MO−141 HOMO-1 (isovalue = -0.03, 0.03). 61
Figure S50. Model **3** isosurface of MO−142 HOMO (isovalue = -0.03, 0.03). 62
Figure S51. Model **3** isosurface of MO−143 LUMO (isovalue = -0.03, 0.03). 62
Figure S52. Model **3** isosurface of MO−144 LUMO+1 (isovalue = -0.03, 0.03). 63
Figure S53. Model **3** isosurface of MO−145 LUMO+2 (isovalue = -0.03, 0.03). 63
Figure S54. Model **3** isosurface of MO−146 LUMO+3 (isovalue = -0.03, 0.03). 64
Figure S55. Model **4** isosurface of MO−104, HOMO-57 (isovalue = -0.03, 0.03). 65
Figure S56. Model **4** isosurface of MO−134, HOMO-27 (isovalue = -0.03, 0.03). 65
Figure S57. Model **4** isosurface of MO−146, HOMO-15 (isovalue = -0.03, 0.03). 66
Figure S58. Model **4** isosurface of MO−147, HOMO-14 (isovalue = -0.03, 0.03). 66
Figure S59. Model **4** isosurface of MO−148, HOMO-13 (isovalue = -0.03, 0.03). 67
Figure S60. Model **4** isosurface of MO−151, HOMO-10 (isovalue = -0.03, 0.03). 67
Figure S61. Model **4** isosurface of MO−159, HOMO-2 (isovalue = -0.03, 0.03). 68
Figure S62. Model **4** isosurface of MO−160, HOMO-1 (isovalue = -0.03, 0.03). 68
Figure S63. Model **4** isosurface of MO−161, HOMO (isovalue = -0.03, 0.03). 69

Figure S64. Model **4** isosurface of MO−162, LUMO (isovalue = -0.03, 0.03). 69

Figure S65. Model **4** isosurface of MO−163, LUMO+1 (isovalue = -0.03, 0.03). 70

Figure S66. Model **4** isosurface of MO−164, LUMO+2 (isovalue = -0.03, 0.03). 70

9. ELF plots 71

Figure S67. Electron localization function (ELF) map of model **3** plotted in the plane defined by Al1 and Al2 and passing through the Al1–Al2 bond critical point (BCP). 71

Figure S68. Electron localization function (ELF) map of model **3** plotted in the plane defined by Al2 and Al4 and passing through the Al2–Al4 bond critical point (BCP). 71

Figure S69. Electron localization function (ELF) map of model **3** plotted in the plane defined by Al2 and Al3 and passing through the Al2–Al3 bond critical point (BCP). 72

Figure S70. Electron localization function (ELF) map of model **3** plotted in the plane defined by Al1 and Al3 and passing through the Al1–Al3 bond critical point (BCP). 72

Figure S71. Electron localization function (ELF) map of model **3** plotted in the plane defined by Al1 and Al4 and passing through the Al1–Al4 bond critical point (BCP). 73

Figure S72. Electron localization function (ELF) map of model **3** plotted in the plane defined by Al3 and Al4 and passing through the Al3–Al4 bond critical point (BCP). 73

Figure S73. Electron localization function (ELF) map of model **4** plotted in the plane defined by Al1 and Al2 and passing through the Al1–Al2 bond critical point (BCP). 74

Figure S74. Electron localization function (ELF) map of model **4** plotted in the plane defined by Al2 and Al4 and passing through the Al2–Al4 bond critical point (BCP). 74

Figure S75. Electron localization function (ELF) map of model **4** plotted in the plane defined by Al2 and Al5 and passing through the Al2–Al5 bond critical point (BCP). 75

Figure S76. Electron localization function (ELF) map of model **4** plotted in the plane defined by Al2 and Al3 and passing through the Al2–Al3 bond critical point (BCP). 75

Figure S77. Electron localization function (ELF) map of model **4** plotted in the plane defined by Al3 and Al4 and passing through the Al3–Al4 bond critical point (BCP). 76

Figure S78. Electron localization function (ELF) map of model **4** plotted in the plane defined by Al4 and Al5 and passing through the Al4–Al5 bond critical point (BCP). 76

Figure S79. Electron localization function (ELF) map of model **4** plotted in the plane defined by Al3 and Al5 and passing through the Al3–Al5 bond critical point (BCP). 77

10. Tabulation of NMR data: 78

Table S1. Comparison of ^{1}H NMR shifts in aluminyl anion K[Al(NDipp)$_2$(CH$_2$)$_3$][13] and Li[Al(NDipp)$_2$(CH$_2$)$_3$] (values reported in ppm) 78

Table S2. *In-situ* relative concentrations of [AlCp^{iPr4}], [K(OB(NDippCH)$_2$)], and [K[Al(OB(NDippCH)$_2$)$_2$]] at different times extracted from ^{1}H NMR spectra. 78

Table S3. *In-situ* relative concentrations of [AlCp*], [K^{Dipp}Nacnac], and [Al^{Dipp}Nacnac] at different times extracted from ^{1}H NMR spectra. 78

Table S4. *In-situ* relative concentrations of [AlCp^{iPr4}], [K^{Dipp}Nacnac], and [Al^{Dipp}Nacnac] at different times extracted from ^{1}H NMR spectra......78

Table S5. *In-situ* relative concentrations of [AlCp^{iPr4}], [$Li_2(NDipp)_2(CH_2)_3(Et_2O)_2$], and [$Li[Al(NDipp)_2(CH_2)_3]$] at different times......78

11. Tabulation of X-ray data......79

Table S6. X-ray Crystallography Data Collection and Refinement Details. 79

12. Tabulation of QTAIM critical points......81

Table S7. Real space functions at critical points. 81

13. DFT coordinates and details......83

13.1 Model structure 3......83

13.2 Model structure 4......91

References: 101

1. Experimental

1.1.1 General Considerations

All manipulations were carried out under an inert N_2 atmosphere using standard glovebox and Schlenk-line techniques.

^{1}H, ^{27}Al, ^{29}Si NMR spectra and 2D correlation spectra (Heteronuclear Single Quantum Coherence (HSQC), Heteronuclear Multiple Bond Correlation (HMBC)) were obtained on a Bruker Avance Neo 400 MHz spectrometer equipped with an Ascend magnet and an automated-tuning 5 mm broadband iProbe. Longer ^{13}C and variable-temperature (VT) NMR experiments were recorded on a Bruker Avance Neo 500 MHz spectrometer using the same probe configuration.

In-situ ^{1}H NMR studies of reactions were conducted using an internal standard, either separated by a flame sealed capillary (hexamethyldisiloxane (HMDSO)) or by addition to the reaction directly as a solvent (C_6H_6). We used the MestReNova software suite[1] to analyze and deconvolute all proton signals, and the areas of the fitted peaks were then used to calculate the yields of each species in solution. Deconvolution was performed by first selecting all peaks observed in the NMR spectrum and then applying a fitting algorithm to fit a Lorentzian–Gaussian curve to each peak. Relative concentrations are calculated and tabulated as [analyte]/[standard].

Single-crystal X-ray diffraction data were collected on a Bruker D8 Advance diffractometer equipped with a microfocus Cu Kα (λ = 1.54178 Å) source (IμS) and a Photon III CPAD detector at 110-130 K. Data collection and integration were carried out using APEX4.[2] Frames were integrated with the Bruker SAINT software package using a narrow-frame algorithm.[3] Data was corrected for absorption effects using the Multi-Scan method (SADABS).[4] The structure was solved through direct methods using the Bruker SHELXTL Software Package.[5] Subsequent model refinement was carried out in Olex2[6] via least-square refinement against F^2 by SHELXL[7]. For all structures, all non-hydrogen atoms were refined anisotropically. All hydrogen atoms were placed in geometrically calculated positions using the riding model and refined isotropically.

Infrared spectra were collected on a Cary 630 FTIR Spectrometer inside an Ar filled glovebox.

Tandem Gas chromatography and mass spectroscopy (GC-MS) were collected on a Thermo Scientific DSQ II spectrometer with a mass range up to 1000 *m/z* and unit mass resolution.

Ultraviolet-Visible absorption (UV-Vis) spectroscopy was collected on a Shimadzu UV-2501PC with a resolution of 0.1 nm wavelength accuracy of 0.3 nm. Spectra were collected from 800 nm to 300 nm at “medium” speed (210 nm/minute). Samples were prepared in a glovebox under an inert N_2 atmosphere and placed into a sealed quartz cuvette. 1 mM samples of **3** and **4** were prepared as solutions in *n*-hexane.

1.1.2 Materials and Methods

The following reagents were purchased commercially and used without further purification: Adogen 464 (MilliporeSigma), potassium hydroxide (>85% flakes, Fisher Scientific), dicyclopentadiene (stabilized, MilliporeSigma), magnesium sulfate (anhydrous, Fisher Scientific), sodium amide (95%, Thermo Scientific Chemicals), bis(2,6-diisopropylphenyl)carbodiimide (VWR), *n*-butyllithium 1.6M in *n*-hexane (Fisher Scientific), Celite 545 (Ficher Scientific) and hexanes (isomeric mixture, BDH/VWR Chemicals).

The solvents tetrahydrofuran (THF, Fisher Scientific), diethyl ether (Et_2O >98%, stabilized, BDH/VWR Chemicals), *n*-hexane (>97%, HiPerSolv CHROMANORM for HPLC, BDH/VWR Chemicals), and toluene (BDH/VWR Chemicals) were dried using a Pure Process Technology solvent purification system. 2-bromopropane (Oakwood Chemical) and 1,2-difluorobenzene were dried over CaH_2 and distilled prior to use. Dimethoxyethane (MilliporeSigma), hexamethyldisiloxane (TCI), deuterated benzene (CIL), and deuterated toluene (CIL) were degassed and dried by benzophenone ketyl formation using sodium metal and subsequently distilled.

The following compounds were synthesized according to published procedures: $[AlCp^*]_4$,[8] K^{Dipp}Nacnac [Nacnac = 1,3-diketiminate, Dipp = 2,6-diisopropylphenyl],[9] $NaNSiMe_3$Dipp,[10] $Li_2(NDipp)_2(CH_2)_3$,[11] and $KOB(NDippCH)_2$.[12] $NaCp^{iPr4}$,[13] and $Li(NDipp)_2C_5H_9$[14] were synthesized following modified literature methods as described below.

1.2.1 Synthesis of $NaCp^{iPr_4}$

1.2.1.1 Triisopropylcyclopentadiene (HCp^{iPr_3})

In a modified literature method,[13] we charged a 2 L three-necked round-bottom flask equipped with a mechanical stirrer, addition funnel, and reflux condenser with Adogen 464 (17.4 g, 0.04 mol) and a 50% w/w aqueous potassium hydroxide solution (prepared from 242 g KOH and 242 mL H_2O). We cooled the mixture in an ice bath and added freshly cracked cyclopentadiene (28.5 g, 0.43 mol) with vigorous stirring. After 0.5 h the mixture turned pink-red. While maintaining cooling, we added 2-bromopropane (212 g, 1.72 mol) dropwise, then gradually raised the temperature to 50 °C over the course of 2 h. The solution darkened from pink to brown as we stirred it for an additional 8 h.

GC–MS analysis of the crude organic layer indicated nearly equal amounts of di- and triisopropylcyclopentadiene isomers. We cooled the mixture, separated the layers, and replaced the aqueous layer with fresh 50% KOH solution (242 g KOH, 242 mL H_2O). We added Adogen 464 (5.0 g, ~0.01 mol) and stirred the mixture at 50 °C for another 8 h. After separating the layers, we extracted the aqueous phase several times with hexanes. We dried the combined organic extracts over $MgSO_4$, filtered, and concentrated them. We distilled the residue under reduced pressure (4–

6 Torr, 90–100 °C) to obtain HCp^{iPr3} isomers (73 g, 0.38 mol, 88%) as a light-yellow liquid. GC–MS retention times: 7.2–8.2 min.

1.2.1.2 Tetraisopropylcyclopentadiene (HCp^{iPr4})

In a modified literature method,[13] we suspended sodium amide (17.8 g, 0.45 mol) in THF (320 mL) in a 2 L three neck round bottom flask equipped with a reflux condenser. We added the triisopropylcyclopentadiene isomers (73 g, 0.38 mol) and refluxed the mixture for 12 h. Ammonia evolution stopped after 10 h, giving a dark-brown mixture. After cooling, we added 2-bromopropane (46.7 g, 0.38 mol) dropwise and stirred the reaction for 1 h as the mixture lightened in color. We then refluxed the mixture gently for 4 h. After cooling, we added water (10 mL) in 1 mL portions. We removed solvent by distillation at 80 °C, extracted the residue with hexanes (3 × 150 mL), dried the solution over $MgSO_4$ then filtered it, before distilling the product under reduced pressure (100 mTorr, 90–100 °C) to yield HCp^{iPr4} as a golden-yellow liquid (65 g, 0.28 mol, 73% yield).

1.2.1.3 Sodium Tetraisopropylcyclopentadienide ($NaCp^{iPr4}$)

In a 500 mL Schlenk flask, we prepared a suspension of sodium amide (13.0 g, 0.33 mol) in THF (290 mL) and added HCp^{iPr4} (65 g, 0.28 mol). We refluxed the mixture for 72 h, during which the color changed from golden yellow to dark brown. We filtered the reaction mixture through Celite 545 and concentrated the filtrate *in vacuo*. We washed the resulting slurry with *n*-hexane (3 × 100 mL), isolated the solid by filtration and heated it at 100 °C *in vacuo* for 2 h to remove volatile byproducts. We washed the pale ivory solid again with small portions of *n*-hexane and dried it *in vacuo* to obtain $NaCp^{iPr4}$ (30. g, 0.12 mol, 42% yield).

FTIR (ATR, cm^{-1}): 2955.3 (s), 2865.5 (s), 1458.8 (m), 1358.7 (m), 1270.9 (w), 1178.5 (m), 1102.0 (m), 986.5 (m), 744.1 (m), 690.3 (m).

1.2.2 Synthesis of $AlCp^{iPr4}$

In a 20 mL vial, we dissolved $NaCp^{iPr4}$ (2.32 g, 9.06 mmol) in 15 mL of 1,2-difluorobenzene and 1 mL of dimethoxyethane. We added $[AlCp^*]_4$ (0.294 g, 0.600 mmol) as a solid and stirred the mixture at 70 °C for 12 h. After cooling, we removed volatiles *in vacuo* and triturated the residue with 5 mL *n*-hexane. We then extracted the residue with *n*-hexane (3 × 5 mL), filtered and concentrated the combined extracts to obtain an amber oil of $AlCp^{iPr4}$ (0.120 g, 0.461 mmol, 25.4%).

^1H NMR (400 MHz, C_6D_6): δ 5.58 (s, 1H, C*H*), 2.94 (sept, J = 7.3 Hz, 2H, C*H*–*i*Pr), 2.79 (sept, J = 7.0 Hz, 2H, C*H*–*i*Pr), 1.31 (dd, J = 7.3 Hz, 12H, $(CH_3)_2$–*i*Pr), 1.22 (d, J = 7.0 Hz, 6H, $(CH_3)_2$–*i*Pr), 1.10 (d, J = 6.8 Hz, 6H, $(CH_3)_2$–*i*Pr).

^{13}C NMR (125.7 MHz, C_6D_6): δ 127, 126 (C_5-Cp*), 25, 24, 23, ($CH(CH_3)_2$).

^{27}Al NMR (130.3 MHz, C_6D_6): δ -158 (Consistent with literature values, -159 ppm).[15]

FTIR (ATR, cm^{-1}): 2962.2 (s), 2926.4 (m), 2870.4 (m), 2166.3 (w), 1631.2 (w), 1570.0 (w), 1460.4 (m), 1365.0 (m), 1315.8 (w), 1259.1 (s), 1179.9 (w), 1100.2 (m), 1019.0 (s), 981.9 (w), 858.8 (w), 799.0 (s), 750.4 (w), 666.2 (w).

1.2.3 Synthesis of [(Li(NDipp)$_2$C$_5$H$_9$)$_2$THF]

In a 100 mL flask, we dissolved bis(2,6-diisopropylphenyl)carbodiimide) (5.0 g, 14 mmol) in 20 mL of THF. We added a 1.6 M *n*-butyllithium solution in *n*-hexane (11 mL, 18 mmol) dropwise and stirred the reaction for 2 h. After stirring, we removed the solvent *in vacuo*, redissolved the residue in 15 mL of boiling *n*-hexane, and placed the solution in a −15 °C freezer to grow off-white crystals of **[(Li(NDipp)$_2$C$_5$H$_9$)$_2$THF]** (6.0 g, 6.5 mmol, 94% yield).

^{1}H NMR (400 MHz, C_6D_6): δ 7.25 (d, *J* = 7.5 Hz, 4H, *meta*-Aryl-C*H*), 7.15-6.94 (m, 7H, Aryl-C*H*), 6.77 (t, *J* = 7.7 Hz, 1H, *para*-Aryl-C*H*), 3.84 (sept, *J* = 6.9 Hz, 2H, C*H*-*i*Pr), 3.65 (sept, *J* = 6.9 Hz, 1H, C*H*-*i*Pr), 3.21 (sept, *J* = 6.8 Hz, 1H, C*H*-*i*Pr), 2.71 (m, 2H, THF-O(C*H*$_2$)$_2$(CH$_2$)$_2$), 2.41 (t, *J* = 8.0 Hz, 2H, CC*H*$_2$(CH$_2$)$_2$CH$_3$), 2.28 (m, 2H, CC*H*$_2$(CH$_2$)$_2$CH$_3$), 1.69, 1.38 (m, 4H, CCH$_2$C*H*$_2$CH$_2$CH$_3$), 1.51 (d, *J* = 6.9 Hz, 12H, (C*H*$_3$)$_2$–*i*Pr), 1.32 (d, *J* = 6.8 Hz, 6H, (C*H*$_3$)$_2$–*i*Pr), 1.29, 1.03 (m, 4H, C(CH$_2$)$_2$C*H*$_2$CH$_3$), 1.25 (d, *J* = 6.8 Hz, 12H, (C*H*$_3$)$_2$–*i*Pr), 1.24 (d, *J* = 6.9 Hz, 6H, (C*H*$_3$)$_2$–*i*Pr), 1.18 (d, *J* = 6.9 Hz, 6H, (C*H*$_3$)$_2$–*i*Pr), 0.86 (d, *J* = 6.7 Hz, 6H, (C*H*$_3$)$_2$–*i*Pr), 0.83 (m, 2H, THF-O(CH$_2$)$_2$(C*H*$_2$)$_2$), 0.78, 0.55 (t, *J* = 7.3 Hz, 6H, C(CH$_2$)$_3$C*H*$_3$).

^{13}C NMR (125.7 MHz, C_6D_6): δ 171 (N-*C*-N), 148-143 (Ar-*C*-*i*Pr), 125-122 (Ar-*C*H), 33-27 (*C*H$_2$), 28-27 (*C*H-*i*Pr), 25-24 (*C*H$_3$-*i*Pr), 14-13 (*C*H$_3$-*n*Bu).

FTIR (ATR, cm^{-1}): 2960.1 (s), 2925.1 (m), 2866.5 (m), 2150.9 (w), 1484.6 (s), 1429.2 (m), 1357.3 (w), 1314.2 (s), 1240.6 (s), 1206.3 (m), 1103.4 (m), 1033.6 (m), 981.6 (w), 928.3 (w), 885.4 (w), 838.1 (w), 787.8 (m), 751.2 (m), 689.7 (w).

SC-XRD of **[(Li(NDipp)$_2$C$_5$H$_9$)$_2$THF]** – Crystals of **[(Li(NDipp)$_2$C$_5$H$_9$)$_2$THF]** were isolated in the $P\bar{1}$ space group. Disorder was modeled for in one of the isopropyl groups (C161, C162, C163) due to thermal rotation of the group. A CH_3 branch is disordered in two sites (C163, C164) with partial occupancies of 0.29(3) and 0.71(3) respectively.

1.2.6 Synthesis of [(Al(NDipp)$_2$C$_5$H$_9$)$_2$C$_7$H$_8$]·C$_7$H$_8$

In a 20 mL vial, we dissolved lithium *n*-butyl amidinate [(Li(NDipp)$_2$C$_5$H$_9$)$_2$THF] (0.200 g, 0.40 mmol) in 15 mL of toluene. We then added [AlCp*]$_4$ (0.065 g, 0.10 mmol) as a solid to the solution of lithium *n*-butyl amidinate and heated the mixture to 60 °C for 36 h while stirring until a white precipitate formed. We filtered the reaction mixture over Celite 545 and concentrated it to one-

quarter volume. We obtained yellow single crystal quality crystals of **[(Al(NDipp)$_2$C$_5$H$_9$)$_2$C$_7$H$_8$]·C$_7$H$_8$** by cooling the solution to −15 °C (0.090 g, 0.401 mmol, 43% yield).

^{1}H NMR (400 MHz, C_6D_6): δ 7.27-6.99 (m, 12H, Aryl-C*H*), 6.10 (t, *J J* = 7.5 Hz, 1H, *para*-tol-C*H*), 5.24 (t, *J* = 6.4 Hz, 1H, *meta*-tol-C*H*), 4.96 (d, *J* = 6.1 Hz, 1H, *ortho*-tol-C*H*), 3.77 (sept, *J* = 6.4 Hz, 1H, C*H*-*i*Pr), 3.69 (sept, *J* = 6.1 Hz, 1H, C*H*-*i*Pr), 3.57-3.49 (m, 4H, C*H*-*i*Pr), 3.25 (sept, *J* = 6.9 Hz, 1H, C*H*-*i*Pr), 3.17 (sept, *J* = 6.5 Hz, 1H, C*H*-*i*Pr), 2.42 (t, *J* = 6.2Hz, 1H, *meta*-tol-C*H*Al), 2.36 (d, *J* = 6.2 Hz, 1H, *ortho*-tol-C*H*Al), 2.19-1.89 (m, 4H, CCH_2(CH$_2$)$_2$CH$_3$), 1.82 (s, 3H, CH_3-tol), 1.62-1.57, 1.36-1.29, 0.82-0.76 (m, 48H, (CH_3)$_2$–*i*Pr), 1.25-1.42 (m, 4H, CCH$_2$CH_2CH$_2$CH$_3$), 0.77-0.69 ,0.65-0.55 (m, 4H, C(CH$_2$)$_2$CH_2CH$_3$), 0.42 (t, *J* = 7.3 Hz, 6H, C(CH$_2$)$_3$CH_3).

^{13}C NMR (125.7 MHz, C_6D_6): δ 155-134 (Aryl-*C*-N, *C*-*i*Pr), 129 (*para*-tol-*C*H), 129-123 (Aryl-C*H*), 119 (*meta*-tol-*C*H), 114 (*ortho*-tol-*C*H), 42 (*ortho*-tol-*C*HAl), 37 (*meta*-tol-*C*HAl), 32 (*C*(CH$_2$)$_3$CH$_3$), 28 (C*C*H$_2$(CH$_2$)$_2$CH$_3$), 28-27 (*C*H-*i*Pr), 26-22 ((*C*H$_3$)$_2$–*i*Pr, CCH$_2$(*C*H$_2$)$_2$CH$_3$), 24 (*C*H$_3$-tol), 13 (C(CH$_2$)$_3$*C*H$_3$).

^{27}Al NMR (130.3 MHz, C_6D_6): No signal observed.

FTIR (ATR, cm^{-1}): 2962.6 (m), 2921.4 (m), 2866.4 (m), 1637.1 (w), 1506.2 (w), 1459.4 (s), 1427.8 (s), 1381.4 (m), 1317.5 (m), 1243.6 (m), 1179.8 (m), 1103.9 (m), 1052.2 (w), 987.0 (w), 935.6 (w), 904.7 (m), 856.5 (s), 790.5 (s), 749.3 (m), 722.0 (w), 680.1 (w).

SC-XRD of **[(Al(NDipp)$_2$C$_5$H$_9$)$_2$C$_7$H$_8$]·C$_7$H$_8$** – Crystals of **[(Al(NDipp)$_2$C$_5$H$_9$)$_2$C$_7$H$_8$]·C$_7$H$_8$** were successfully modeled in both the *P*2/*n* and *Pn* space groups. Owing to the asymmetry of the toluene molecules and the connectivity of the *n*-butyl groups between neighboring molecules, the structure is best described in the *Pn* space group. The cycloaddition of toluene appears aperiodic, giving rise to four possible occupancy patterns. Positional disorder in the methyl group of the toluene was modeled for using a linear combination of free-variable occupancies (SUMP). Consequently, atoms C2A, C3A, C5A, and C6A refined to occupancies of 0.23(2), 0.16(2), 0.35(2), and 0.25(2), respectively.

The terminal CH_2CH_3 group of the amidinate ligand exhibited two disordered positions: one oriented toward the neighboring moiety in the crystal packing and the other oriented toward the intermolecular void space. Because the first CH_2CH_3 group points toward another disordered CH_2CH_3 group in the lattice, significant overlap required placement of the two disordered ethyl groups (C116, C117 and C216, C217) in out-of-phase PART instructions. The refined occupancies for the CH_2CH_3 groups (C114, C115), (C116, C117), (C214, 215) and (C216, C217) were 0.61(2), 0.39(2), 0.362(18), and 0.638(18), respectively.

One isopropyl substituent (C361, C362, C363) displayed rotational disorder. This was modeled using two positional sites for the methyl groups (C362, C363) and (C364, C365) with refined occupancies of 0.325(18) and 0.675(18), respectively.

Lastly, modeling of outer sphere toluene molecules was attempted. However, due to thermal motion and high disorder, the solvent density was masked using SQUEEZE. The solvent mask found 86 electrons in a volume of 334 $Å^3$ per unit cell. This is consistent with the presence of one toluene per asymmetric unit which accounts for 100 electrons per unit cell.

1.2.10 Synthesis of $[AlN(SiMe_3)DippAlCp^*]_2{\cdot}C_6H_{14}$

We dissolved $[NaN(SiMe_3)(Dipp)](THF)_3$ (0.100 g, 0.205 mmol) and $[AlCp^*]_4$ (0.033 g, 0.051 mmol) in 10 mL of toluene. The reagents were stirred and heated to 60°C over the course of 12 h. We filtered the resulting orange solution and obtained orange precipitate by removing the solvent *in vacuo*. Orange crystals of **$[AlN(SiMe_3)(Dipp)]_4{\cdot}C_6H_{14}$** were isolated by dissolving the orange precipitate in 10 mL of boiling *n*-hexane followed by subsequent hot filtration and cooling to –15 °C in a freezer overnight (0.015 g, 0.053 mmol, 68% yield).

^{1}H NMR (400 MHz, C_6D_6): δ 7.17 (d, *J* = 8.1 Hz, 4H, *meta*-Aryl-C*H*), 7.11-7.07 (m, 2H, *ortho*-Aryl-C*H*), 3.84 (sept, *J* = 6.9 Hz, 4H, C*H*-*i*Pr), 1.71 (s, 30H, C*H*$_3$-Cp*), 1.51 (d, *J* = 6.9 Hz, 12H, C*H*$_3$-*i*Pr), 1.33 (d, *J* = 6.9 Hz, 12H, C*H*$_3$-*i*Pr), 1.23 (m, 8H, *n*-hexane-C*H*$_2$), 0.89 (t, *J* = 6.6, 6H, *n*-hexane-C*H*$_3$), 0.39 (s, 18H, Si(C*H*$_3$)$_3$).

^{13}C NMR (125.7 MHz, C_6D_6): δ 127, 123 (Aryl-*C*H), 32, 24 (*n*-hexane-*C*H$_2$), 28 (*C*H-*i*Pr), 27, 24 (*C*H$_3$-*i*Pr), 14 (*n*-hexane-*C*H$_3$), 10 (*C*H$_3$-Cp*), 3 (Si(*C*H$_3$)$_3$).

^{27}Al NMR (130.3 MHz, C_6D_6): δ –45, –62 (tetramer – *Al*NTMSDipp, *Al*Cp*)

FTIR (ATR, cm^{-1}): 3053.0 (w), 2960.1 (m), 2918.5 (m), 2862.7 (m), 1982.1 (w), 1584.5 (w), 1492.2 (w), 1459.7 (m), 1426.1 (s), 1378.6 (m), 1313.1 (m), 1239.2 (s), 1178.5 (s), 1102.8 (m), 1042.2 (w), 900.9 (s), 831.1 (s), 787.0 (s), 742.6 (m), 678.4 (m).

SC-XRD of **$[AlN(SiMe_3)DippAlCp^*]_2{\cdot}C_6H_{14}$** – Crystals of **$[AlN(SiMe_3)DippAlCp^*]_2{\cdot}C_6H_{14}$** were isolated in the *C*2/*c* space group. All crystalline material were thin plates and as such attempts at isolating a single crystal were unsuccessful. Data used came from a twinned crystal containing two domains and a crystal fraction of 0.412(2).

An outer-sphere *n*-hexane solvent molecule has a twofold rotational disorder and lies on rotational symmetry element. As such the molecule is placed into a negative PART and the generation of special position constraints is suppressed and bonds to symmetrically generated atoms with the same or a different non-zero PART number are excluded. A such, partial occupancies of the solvent

sites then must be assumed to 0.5 due to the symmetrical generation of the other disordered moiety. Refinement of the occupancy of the *n*-hexane molecule results in an occupancy of 0.503(7).

UV-Vis of **$[AlN(SiMe_3)DippAlCp^*]_2 \cdot C_6H_{14}$ - π → π*** (λ_{max} = 328 nm, ε = 460 $M^{-1}cm^{-1}$).

1.2.8 Synthesis of $[Al(N_2Dipp_2C_3H_6)(Al_4Cp^*_3)] \cdot 0.5C_6H_6$

In a 20 mL vial, we added $Li_2[(NDipp)_2(CH_2)_3](Et_2O)_2$ (0.080 g, 0.144 mmol) and $[AlCp^*]_4$ (0.118 g, 0.182 mmol) in toluene. We then heated and stirred the reaction mixture at 60 °C for three days which resulted in a dark green solution and a grey precipitate. We then removed the solvent *in vacuo*, and the remaining solid precipitate was extracted three times with 2 mL of *n*-hexane. The *n*-hexane solution was concentrated to 2 mL and stored overnight at –15 °C to crystallize excess $[AlCp^*]_4$, and the remaining green solution was decanted. The solvent was removed *in vacuo* from the filtrate, and we then triturated the green crude solid twice with 2 mL of HMDSO to yield a blue precipitate. High quality single crystals of **$[Al(N_2Dipp_2C_3H_6)(Al_4Cp^*_3)] \cdot 0.5C_6H_6$** were grown by vapor diffusion of HMDSO into a concentrated solution in *n*-hexane (0.032 g, 0.033 mmol, 23% yield).

^1H NMR (400 MHz, C_6D_6): δ 7.26 – 6.99 (m, 6H, Aryl-C*H*), 3.83 (sept, *J* = 7.0 Hz, 4H, C*H*-*i*Pr), 2.78 , 2.56 (t, *J* = 7.1 Hz, 2H, $(CH_2)_2CH_2$), 2.99 (t, *J* = 5.3 Hz, 4H, $(CH_2)_2CH_2$), 1.78 (s, 45H, Cp^*-CH_3), 1.69 (d, *J* = 6.9 Hz, 12 H, $(CH_3)_2$–*i*Pr), 1.43 (d, *J* = 6.9 Hz, 12H, $(CH_3)_2$–*i*Pr).

^{13}C NMR (125.7 MHz, C_6D_6): δ 147 (*C*-$CH(CH_3)_2$), 124 (Aryl-*C*H), 114 (*C*-CH_3-Cp^*), 57 $(CH_2)_2CH_2$, 28 (*C*H-*i*Pr), 25 $(CH_3)_2$-*i*Pr), 11 (*C*H_3-Cp^*).

^{27}Al NMR (130.3 MHz, C_6D_6): δ -37 (br), -79 (br).

FTIR (ATR, cm^{-1}): 2959.0 (s), 2912.3 (s), 2827.0 (s), 2167.9 (w), 1980.5 (w), 1433.9 (s), 1367.8 (s), 1313.9 (m), 1259.4 (w), 1238.3 (w), 1192.6 (w), 1119.7 (s), 1096.1 (m), 1046.6 (w), 966.9 (m), 902.8 (m), 856.1 (s), 796.0 (s), 750.7 (m), 714.5 (w).

SC-XRD of **$[Al(N_2Dipp_2C_3H_6)(Al_4Cp^*_3)] \cdot 0.5C_6H_6$** – Crystals of **$[Al(N_2Dipp_2C_3H_6)(Al_4Cp^*_3)] \cdot 0.5C_6H_6$** were isolated in the $P\bar{1}$ space group.

UV-Vis of **$[Al(N_2Dipp_2C_3H_6)(Al_4Cp^*_3)] \cdot 0.5C_6H_6$ - π → π***, λ_{max} = 330 nm (ε = 455 $M^{-1}cm^{-1}$); λ_{max} = 394 nm (ε = 110 $M^{-1}cm^{-1}$); λ_{max} = 580 nm (ε = 36 $M^{-1}cm^{-1}$).

1.2.4 Synthesis of K[Al(OB(NDippCH)$_2$)$_2$]

1.2.4.1 Method A – [AlCp*]$_4$

Following the procedure from Sarkar *et al.*, we dissolved K[OB(NDippCH)$_2$] (4.87 g, 11.0 mmol) in 25 mL of toluene.[16] We added [AlCp*]$_4$ (0.893 g, 1.38 mmol) and heated the mixture to 80 °C for 4 h with stirring. We filtered the resulting solution through Celite 545, concentrated it to half-volume, and cooled it to −15 °C to obtain light-yellow crystals of K**[Al(OB(NDippCH)$_2$)$_2$]** (4.56 g, 5.22 mmol, 94.9% yield).

1.2.4.2 Method B – AlCp$^{i\mathrm{Pr}4}$

In an NMR tube, we dissolved AlCp$^{i\mathrm{Pr}4}$ (0.011 g, 0.042 mmol) and the potassium salt K[OB(NDippCH)$_2$] (0.031 g, 0.070 mmol) in 0.60 mL of C_6D_6. After 0.5 h, ^{1}H NMR indicated complete conversion to K**[Al(OB(NDippCH)$_2$)$_2$]** (92% yield). Because the reaction was rapid, we could not quantify yield with an internal standard; instead, we determined the yield relative to the sample concentration and the protic content of the solvent (see below).

Observed ^{1}H, ^{13}C, signals are consistent with those reported in literature. No signal was observed in the ^{27}Al NMR spectrum.[16]

Synthesis of K[Al(OB(NDippCH)$_2$)$_2$] by reaction of AlCp$^{i\mathrm{Pr}4}$ and K[OB(NDippCH)$_2$]: *in-situ* studies – The reaction between AlCp$^{i\mathrm{Pr}4}$ and K[OB(NDippCH)$_2$] progresses too rapidly to determine the initial concentration of the reagents, as such the yield is calculated referenced to the 0.32(1)% H NMR solvent (36(1) mM C_6H_6). The initial ratio of [reagent]/[C_6H_6] was determined based on mass of added material is 0.042(1) mmol AlCp$^{i\mathrm{Pr}4}$ and 0.070(1) mmol K[OB(NDippCH)$_2$] in 0.6 mL of benzene resulting in a concentration of 84(2) mM AlCp$^{i\mathrm{Pr}4}$ and 140(2) mM K(OB(NDippCH)$_2$). Relative concentrations are reported in Table S2. The calculated *in-situ* yield is 92(6)% of K[Al(OB(NDippCH)$_2$)$_2$].

1.2.5 Synthesis of Al^{Dipp}Nacnac

1.2.5.1 Method A – [AlCp*]$_4$

Following an approach adapted from Kysliak *et al.*,[17] we dissolved K^{Dipp}Nacnac (0.013 g, 0.028 mmol) and [AlCp*]$_4$ (0.0046 g, 0.007 mmol) in 0.60 mL C_6D_6 in an NMR tube. After acquiring an initial ^{1}H NMR spectrum, we heated the mixture to 70 °C for 48 h. A second spectrum showed complete consumption of K^{Dipp}Nacnac. We filtered the reaction over Celite 545 and obtained amber crystals of Al^{Dipp}Nacnac by slow evaporation, along with small yellow crystals of [AlCp*]$_4$. Due to the mixture of isolated products, an *in-situ* NMR study was conducted to determine the yield of the product based of consumed reagents. Another NMR scale reaction was conducted by dissolving K^{Dipp}Nacnac (0.015 g, 0.032 mmol) and [AlCp*]$_4$ (0.0053 g, 0.008 mmol) in 0.60 mL C_6D_6 in an NMR tube. After heating at 60 °C for 4 h, 6.0% of the limiting reagent (K^{Dipp}Nacnac)

had converted to Al^{Dipp}Nacnac with a yield of 64% based on consumed AlCp* and 20% based on consumed K^{Dipp}Nacnac.

Synthesis of Al^{Dipp}Nacnac by reaction of $[AlCp^{*}]_4$ and K^{Dipp}Nacnac: *in-situ* studies – The reaction between K^{Dipp}Nacnac and $[AlCp^{*}]_4$ was heated at 60 °C for 4 h. After this time only a small fraction of the reagent had reacted. An internal standard of HMDSO was used to reference the NMR signals of the starting material to determine the % yield of the product. Due to the overlap of signals in the ^{1}H NMR spectra, only a select few signals are sufficiently isolated from other chemical shifts to be deconvoluted. Specifically, the diagnostic ^{1}H chemical shifts used to determine the concentration of K^{Dipp}Nacnac are the signal assigned to the CH_3 (d) from the Dipp groups of the ligand at 1.25 and 1.06 ppm, the signal assigned to the CH_3 (s) at 1.88 ppm, and the signal assigned to the C*H* (s) protons at 4.81 ppm. The diagnostic ^{1}H chemical shifts used to determine the concentration of AlCp* (calculated as monomer) is the CH_3 (s) peak at 1.89 ppm. The diagnostic ^{1}H NMR shift used for the Al^{Dipp}Nacnac is the signal assigned to the CH_3 (d) from the Dipp groups of the ligand at 1.37 and 1.13 ppm, the CH_3 (s) at 1.65 ppm, and the C*H* (s) protons at 5.17 ppm. Lastly, the diagnostic ^{1}H chemical shift used to reference HMDSO is at 0.18 ppm. Relative concentrations of reagents and products are reported in Table S3. The calculated *in-situ* yield / % consumed AlCp* is 64% and *in-situ* yield / % consumed K^{Dipp}Nacnac is 20% at a conversion of 6.0% of the K^{Dipp}Nacnac in 4 h.

1.2.5.2 Method B – AlCp^{iPr4}

In an NMR tube, AlCp^{iPr4} (0.011 g, 0.044 mmol) and K^{Dipp}Nacnac (0.018 g, 0.040 mmol) were dissolved and mixed in a solution of 0.60 mL C_6D_6 with an internal standard of benzene. After 16 h at room temperature, a ^{1}H NMR spectrum was collected, and no change was observed. As such the same reaction mixture was heated to 60 °C for 2 h; however, no reaction was observed. The reaction was then heated to 70 °C for another 4 h at which point 6.2% of the limiting reagent (K^{Dipp}Nacnac) had converted to Al^{Dipp}Nacnac with a yield of 56% based on consumed AlCp^{iPr4} and 15% based on consumed K^{Dipp}Nacnac.[17]

^{1}H, and ^{13}C signals match with those reported in literature.[18]

Synthesis of Al^{Dipp}Nacnac by reaction of AlCp^{iPr4} and K^{Dipp}Nacnac: *in-situ* studies – The reaction between K^{Dipp}Nacnac and AlCp^{iPr4} was initially attempted at room temperature; however, due to negligible reaction, the reaction was performed again at elevated temperature, first at 60 °C for 2 h, then at 70 °C for another 4 h. After this time only a small fraction of the starting reagents had reacted. An internal standard of protic solvent (C_6H_6) was used to reference the concentration of contents to determine the % yield of the product. Due to the overlap of signals in the ^{1}H NMR spectra, only a select few signals are sufficiently isolated from other chemical shifts to be deconvoluted. Specifically, the diagnostic ^{1}H chemical shifts used to determine the concentration

of K^{Dipp}Nacnac are the signal assigned to C*H*$_3$ (d) from the Dipp groups of the ligand at 1.26 ppm and 1.07 ppm, the signal assigned to C*H*$_3$ (s) at 1.88 ppm, and the signal assigned to C*H* (s) at 4.81 ppm. The diagnostic ^{1}H chemical shifts used to determine the concentration of AlCp^{iPr4} are the signal assigned to C*H*$_3$ (d) from the *i*Pr at 1.32 ppm, 1.30 ppm, 1.22 ppm, 1.10 ppm, and the signal assigned to C*H* (s) at 5.58 ppm. The diagnostic ^{1}H NMR shift used to determine the concentration of Al^{Dipp}Nacnac are the signal assigned to the C*H*$_3$ (d) from the Dipp groups of the ligand at 1.37 and 1.13 ppm, the signal assigned to C*H*$_3$ (s) at 1.65 ppm, and the signal assigned to C*H* (s) protons at 5.17 ppm. Lastly, the diagnostic ^{1}H chemical shift used to reference C_6H_6 is at 7.16 ppm. The relative concentrations of reagents and products are reported in Table S3. The calculated *in-situ* yield / % consumed AlCp^{iPr4} is 56% and *in-situ* yield / % consumed K^{Dipp}Nacnac is 15% at a conversion of 6.2% of K^{Dipp}Nacnac in the total 6 h of heating.

1.2.7 Synthesis of [(Al(NDipp)$_2$C$_5$H$_9$)$_2$C$_6$D$_6$]·C$_6$H$_{14}$

In an NMR tube, we dissolved [(Li(NDipp)$_2$C$_5$H$_9$)$_2$THF] (0.0062 g, 0.024 mmol) and AlCp^{iPr_4} (0.0078 g, 0.018 mmol) in 0.60 mL C_6D_6. The reagents reacted slowly at room temperature for over 40 days. We filtered the resulting yellow-green solution and obtained bright yellow crystals by removing solvent *in vacuo*. X-ray quality single crystals were obtained by redissolving the isolated product **[(Al(NDipp)$_2$C$_5$H$_9$)$_2$C$_6$D$_6$]·C$_6$H$_{14}$** in minimal *n*-hexane (0.2 mL) and placing in a –15 °C freezer overnight (0.005g, 0.005mmol, 60% yield).

^{1}H NMR (400 MHz, C_6D_6): δ 7.22 – 7.02 (m, 12H, Aryl-C*H*), 3.65, 3.18 (br, 4H, C*H*-*i*Pr), 3.52 (sept, *J* = 4H, 6.7Hz, C*H*-*i*Pr), 2.10, 1.92 (br, 4H, C*H*$_2$(CH$_2$)$_2$CH$_3$), 1.59 (br, 6H, C*H*$_3$-*i*Pr), 1.33 (br, 4H, (CH$_2$C*H*$_2$CH$_2$CH$_3$), 1.23 (m, 36H, C*H*$_3$-*i*Pr), 0.75 (br, 6H, C*H*$_3$-*i*Pr), 0.60 (br, 4H, (CH$_2$)$_2$C*H*$_2$CH$_3$), 0.4 (t, *J* = 7.0 Hz, 6H, (CH$_2$)$_3$C*H*$_3$).

^{13}C NMR (125.7 MHz, C_6D_6): δ 145 (*i*Pr-*C*-Aryl) 128 (Aryl-*C*H), 29 (*C*H-*i*Pr), 24 (*C*H$_3$-*i*Pr), 27, 21 (CH$_2$) 13 (CH$_2$)$_3$*C*H$_3$).

^{27}Al NMR (130.3 MHz, C_6D_6): No observed signal.

FTIR (ATR, cm^{-1}): 3056.9 (w), 2962.8 (s), 2925.6 (m), 2870.0 (m), 1607.6 (w), 1455.7 (s), 1422.2 (m), 1370.6 (w), 1340.5 (w), 1313.8 (m), 1253.7 (m), 1169.9 (w), 1102.0 (m), 1051.9 (w), 926.9 (w), 857.1 (w), 793.3 (s), 753.2 (m), 728.4 (m) 690.4 (w).

SC-XRD of **[(Al(NDipp)$_2$C$_5$H$_9$)$_2$C$_6$D$_6$]·C$_6$H$_{14}$** – Crystals **[(Al(NDipp)$_2$C$_5$H$_9$)$_2$C$_6$D$_6$]·C$_6$H$_{14}$** were isolated in the *Pn* space group. A solvent mask was calculated, and 98 electrons were found in a volume of 478 Å^3 per unit cell. This is consistent with the presence of one *n*-hexane per asymmetric unit which account for 100 electrons per unit cell.

1.2.11 Synthesis of [AlN(SiMe$_3$)(Dipp)]$_4$

We dissolved [NaN(SiMe$_3$)(Dipp)(THF)$_3$] (0.054 g, 0.011 mmol) and AlCp^{iPr4} (0.018 g, 0.0069 mmol) in 0.60 mL C_6D_6 in an NMR tube. The reagents reacted slowly at room temperature over the course of 36 h. We filtered the resulting yellow-green solution and obtained yellow precipitate by removing the solvent *in vacuo*. Attempts to grow single crystals of **[AlN(SiMe$_3$)(Dipp)]$_4$** from solution yielded yellow amorphous material containing **[AlN(SiMe$_3$)(Dipp)]$_4$** (0.010 g, 0.053 mmol, 68% yield).

^{1}H, ^{13}C, signals are in close agreement with that reported in literature (literature solvent used ^{i}Pr$_2$O compared with C_6D_6).[19]

1.2.9 Synthesis of [LiAl(NDipp)$_2$(CH$_2$)$_3$]

We dissolved AlCp^{iPr4} (0.016 g, 0.061 mmol) and Li$_2$[(NDipp)$_2$(CH$_2$)$_3$](Et$_2$O)$_2$] (0.021 g, 0.041 mmol) in 0.60 mL C_6D_6 in an NMR tube. After 30 min, ^{1}H NMR showed 50% conversion of Li$_2$(NDipp)$_2$(CH$_2$)$_3$ to the aluminyl salt **[LiAl(NDipp)$_2$(CH$_2$)$_3$]**; after 15 h, conversion reached 98% with an *in-situ* yield of 77% based on the consumption of AlCp^{iPr4}. Attempts to isolate crystalline material of the aluminyl anion were unsuccessful. As such, *in-situ* ^{1}H NMR studies were used to determine the yield of **[LiAl(NDipp)$_2$(CH$_2$)$_3$]** from 0.5 h to 15 h.

^{1}H NMR (400 MHz, C_6D_6): δ 7.33 (d, J = 7.5 Hz, 4H, *meta*-Aryl-C*H*), 7.25 (m, 2H, *ortho*-Aryl-C*H*), 4.33 (sept, J = 6.9 Hz, 4H, C*H*-*i*Pr), 3.52 (t, J = 5.0 Hz, 4H, (C*H*$_2$)$_2$CH$_2$), 2.48 (m, 2H, (CH$_2$)$_2$C*H*$_2$), 1.60 (d, J = 6.8 Hz, 12H, (C*H*$_3$)$_2$–*i*Pr), 1.53 (d, J = 6.9 Hz, 12H, (C*H*$_3$)$_2$–*i*Pr).

^{13}C NMR (125.7 MHz, C_6D_6): δ 123 (Aryl-*C*H), 56 (*C*H$_2$)$_2$CH$_2$, 36 ((CH$_2$)$_2$*C*H$_2$) 28 (*C*H-*i*Pr), 26 (*C*H$_3$)$_2$-*i*Pr).

^{27}Al NMR (130.3 MHz, C_6D_6): No signal observed.

Synthesis of Li[Al(NDipp)$_2$(CH$_2$)$_3$] by reaction of AlCp^{iPr4} and Li$_2$[(NDipp)$_2$(CH$_2$)$_3$](Et$_2$O)$_2$]: *in-situ* studies –The reaction between Li$_2$[(NDipp)$_2$(CH$_2$)$_3$](Et$_2$O)$_2$] and AlCp^{iPr4} was run at room temperature over the course of 15 h. The initial relative concentration of reagents AlCp^{iPr4} and Li$_2$[(NDipp)$_2$(CH$_2$)$_3$](Et$_2$O)$_2$] are determined using an internal standard of HMDSO. Due to the overlap of signals in the ^{1}H NMR spectra, only a select few signals are sufficiently isolated from other chemical shifts to be deconvoluted. Specifically, the diagnostic ^{1}H chemical shifts used to determine the concentration of Li$_2$(NDipp)$_2$(CH$_2$)$_3$(Et$_2$O)$_2$ are the signals corresponding to C*H*$_2$ (pent) at 1.80 ppm, C*H*$_2$ (t) at 3.20 ppm, and C*H* (sept) at 3.79. The diagnostic ^{1}H chemical shifts used to determine the concentration of AlCp^{iPr4} are the signals corresponding to C*H*$_3$ (d) at 1.32 ppm, 1.30 ppm, 1.22 ppm, 1.10 ppm, and C*H* (s) at 5.58 ppm. The diagnostic ^{1}H NMR signal used to determine the concentration of Li[Al(NDipp)$_2$(CH$_2$)$_3$] are the signals corresponding to C*H*$_3$ (d) at 1.60 and 1.53 ppm, the C*H*$_2$ (m) at 2.48 ppm, C*H*$_2$ (t) at 3.52 ppm, and C*H* (sept) at 4.33 ppm.

Lastly, the diagnostic ^{1}H chemical shift used to reference HMDSO is at 0.18 ppm. The relative concentrations of reagents and products are reported in Table S5. The calculated *in-situ* yield is 77%.

2 Calculation of the effective radius of fragments in [Al(N_2Dipp$_2$$C_3$$H_6$)($Al_4Cp^*_3$)]

2.1 Empirical estimate to the effective ionic radius of [Al(N_2Dipp$_2$$C_3$$H_6$)]$^-$

To estimate the effective ionic radius of the aluminyl anion [Al(N_2Dipp$_2$$C_3$$H_6$)]$^-$, we compare the bond lengths of the aluminyl anion interacting with electropositive metals. The aluminyl anion [Al(N_2Dipp$_2$$C_3$$H_6$)]$^-$ interactions as seen in crystallographic structures [(N_2Dipp$_2$$C_3$$H_6$)AlSc(THF)(N(SiMe$_3$)$_2$)$_2$], [($N_2$Dipp$_2$$C_3$$H_6$)AlSc(THF)N(^{i}Pr$_2$)$_2$][20] and [(($N_2$Dipp$_2$$C_3$$H_6$)Al)$_2$Sm(THF)$_4$][21] were used to calculate the effective ionic radius assuming the ionic radius of the Sc^{3+} and Sm^{2+} centers to be 0.745 Å and 1.22 Å respectively.[22] All unique Al-Sc bond lengths in [(N_2Dipp$_2$$C_3$$H_6$)AlSc(THF)(N(SiMe$_3$)$_2$)$_2$] and [($N_2$Dipp$_2$$C_3$$H_6$)AlSc(THF)N(^{i}Pr$_2$)$_2$] are 3.018(1), 2.976(1), 3.0395(5). Subtracting the ionic radius of the Sc^{3+} center from these bond lengths results in an observed [Al(N_2Dipp$_2$$C_3$$H_6$)]$^-$ ionic radii of 2.295, 2.231, 2.273 Å. The observed Al-Sm bond lengths in [((N_2Dipp$_2$$C_3$$H_6$)Al)$_2$Sm(THF)$_4$] is 3.543(1) and 3.518(1). Subtracting the ionic radius of the Sm^{2+} center from these bond lengths results in an observed [Al(N_2Dipp$_2$$C_3$$H_6$)]$^-$ ionic radii of 2.298 and 2.323 Å. All together this results in an average [Al(N_2Dipp$_2$$C_3$$H_6$)]$^-$ ionic radius of 2.288(11) Å.

2.2 Empirical estimate to the effective ionic radius of [Al_4Cp$^*_3$]$^+$

To estimate the effective ionic radius of the [Al_4Cp$^*_3$]$^+$ cluster we will subtract the ionic radius of [(SiMe$_3$)$_2$N]$^-$ from the heteroleptic structure [(SiMe$_3$)$_2$NAl_4Cp$^*_3$].[15] To estimate the ionic radius of [(SiMe$_3$)$_2$N]$^-$ we use the ionic homoleptic structures [Al(N(SiMe$_3$)$_2$)],[23] [Sc(N(SiMe$_3$)$_2$)],[24, 25] [Y(N(SiMe$_3$)$_2$)]·C_6H_6,[26] and [La(N(SiMe$_3$)$_2$)][27] and their associated effective ionic radius for the Al^{3+}, Sc^{3+}, Y^{3+}, and La^{3+} cationic centers (0.535, 0.745, 0.90 and 1.032 Å respectively).[22] The following list is the observed bond distance from their crystallographic structures in Å: [Al(N(SiMe$_3$)$_2$)] – 1.832, 1.834, 1.834; [Sc(N(SiMe$_3$)$_2$)] – 2.056, 2.055, 2.056; [Y(N(SiMe$_3$)$_2$)]·C_6H_6 – 2.224, 2.224, 2.222; [La(N(SiMe$_3$)$_2$)] – 2.361, 2.361, 2.361. Subtracting all of these distance from their corresponding metal center's effective ionic radius result in an average [(SiMe$_3$)$_2$N]$^-$ ionic radius of 1.315(11) Å. The Al-N bond length in [(SiMe$_3$)$_2$NAl_4Cp$^*_3$] is 1.846(2) Å. Subtraction of the effective radius of [(SiMe$_3$)$_2$N]$^-$ from this Al-N bond length results in an effective atomic radius for [Al_4Cp$^*_3$]$^+$ of 0.53(1) Å.

3 DFT, QTAIM, ELF

3.1 Calculation details

3.1.1 Density functional theory (DFT)

To reduce computational cost, truncated models of **3** and **4** were constructed using the following substitutions: Cp^* → Cp, $SiMe_3$ → SiH_3, and Dipp → 2,6-dimethylphenyl. Initial geometries were derived from the corresponding solid-state crystal structures. For the model of **4**, the Al1–Al2 distance was constrained to the crystallographic value because ligand truncation reduced steric congestion and led to substantial shortening of this contact upon unconstrained optimization. The model of **3** converged to a true minimum (no imaginary frequencies). The model of **4** converged to a low-energy first-order saddle point featuring a single imaginary frequency; the associated vibrational mode corresponds to Cp ring rotation and does not significantly perturb the Al–Al interactions of interest. Accordingly, this stationary point was retained for subsequent bonding analyses.

All DFT calculations were performed in ORCA[28] (libint2 for two-electron integrals; libXC support enabled). Dispersion was treated using Grimme's D3 correction with Becke–Johnson damping (D3BJ).[29] Geometry optimizations used the B3LYP functional with the def2-SVP orbital basis set and the def2/J auxiliary basis set, employing the chain-of-spheres approximation (COSX) for efficient evaluation of exchange.[30-40]

"TightSCF" instructions were used with SCF convergence thresholds set to $\Delta E = 1.0 \times 10^{-8}$ Eh, RMS density = 5.0×10^{-9} a.u., max density = 1.0×10^{-7} a.u., DIIS error = 5.0×10^{-7} a.u., and orbital gradient/rotation angle = 1.0×10^{-5} a.u. "TightOPT" instructions were used with geometry optimizations thresholds of $\Delta E = 1.0 \times 10^{-6}$ Eh, max $|\nabla E| = 1.0 \times 10^{-4}$ Eh·bohr^{-1}, RMS $|\nabla E| = 3.0 \times 10^{-5}$ Eh·bohr^{-1}, max displacement = 1.0×10^{-3} bohr, and RMS displacement = 6.0×10^{-4} bohr.

3.1.2 Quantum theory of atoms in molecules (QTAIM) and electron localization function (ELF)

All topological and basin analyses were completed using the software Multiwfn.[41] QTAIM analyses were performed on the converged DFT wavefunctions to locate bond critical points (BCPs) and to extract the electron density $\rho(r)$, Laplacian $\nabla^2\rho(r)$, and total energy density $H(r)$ at each BCP, along with atomic properties including QTAIM charges, localization indices, and pairwise delocalization indices for the relevant Al–Al contacts.[42] Electron localization function (ELF) analyses were then carried out to identify the disynaptic basin associated with the Al–Al interactions and to quantify the electron population and fragment contributions via basin

partitioning.[31, 43] Visualization of the resulting QTAIM/ELF topologies (critical points, bond paths, and ELF isosurfaces/maps) was prepared in Multiwfn/VMD[44] and rendered with POV-Ray.[45]

4 NMR Data:

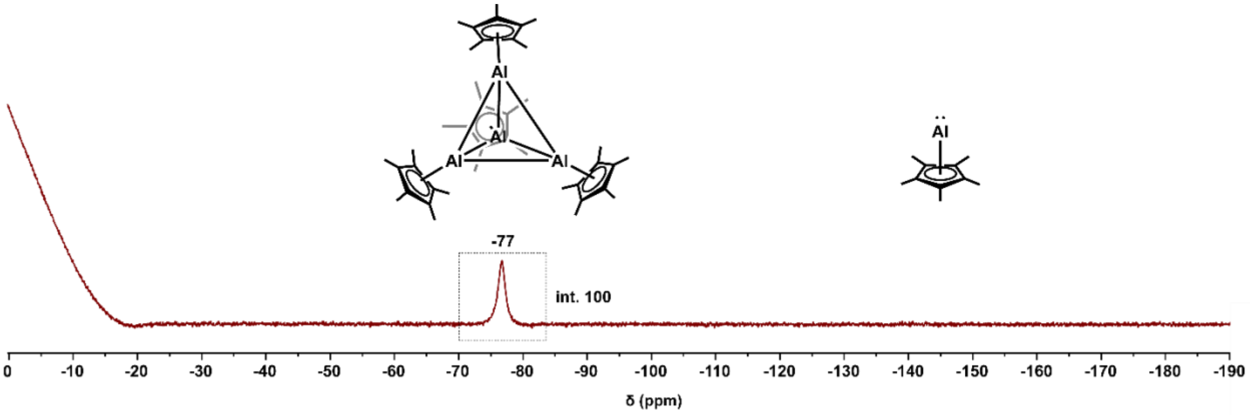

Figure S1. ^{27}Al NMR of **[AlCp*]₄** at room temperature where the relative integral of monomer to tetramer is 0:100.

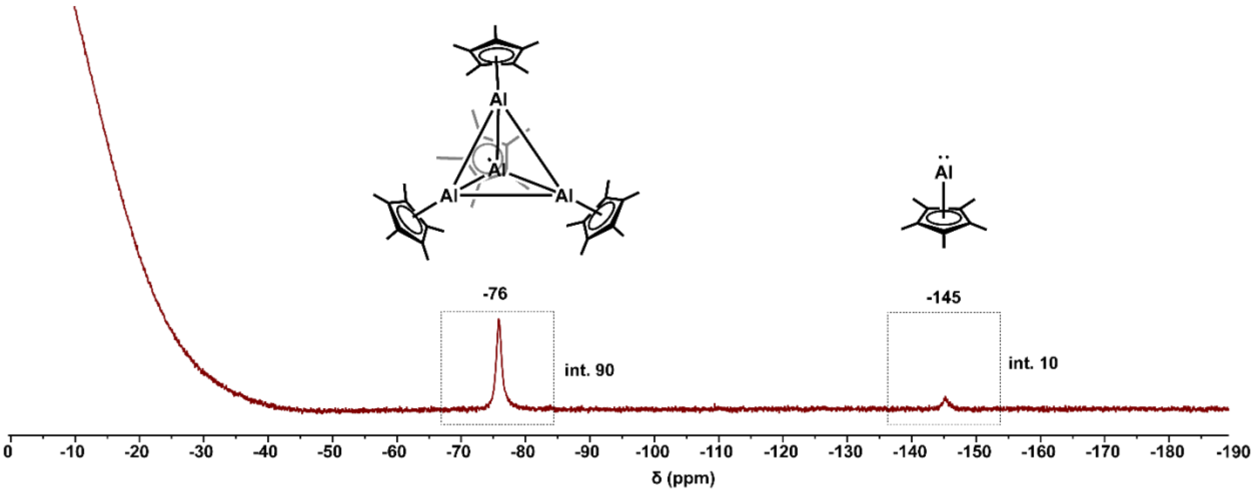

Figure S2. ^{27}Al NMR of **[AlCp*]₄** at 65 °C where the relative integral of monomer to tetramer is 10:90.

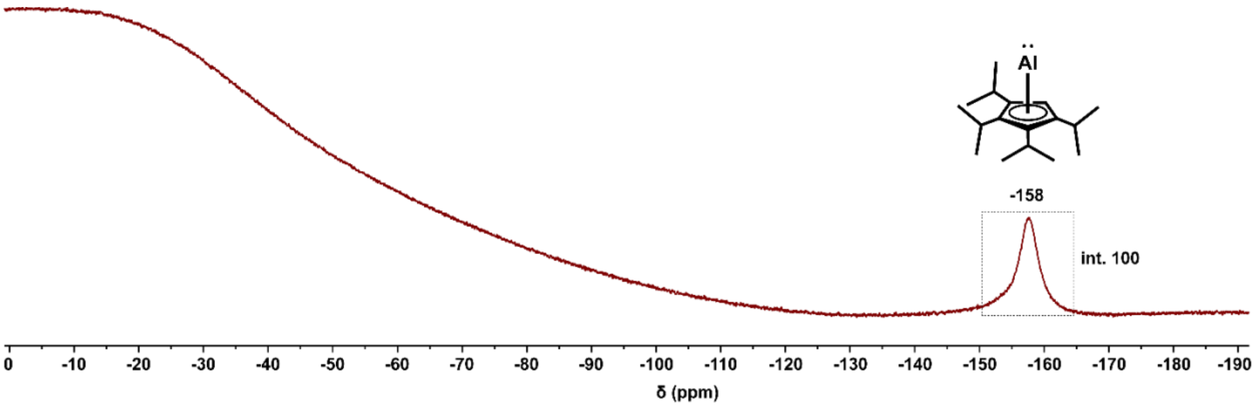

Figure S3. ^{27}Al NMR of **AlCp^{iPr4}** at room temperature where the relative integral of monomer to tetramer is 100:0.

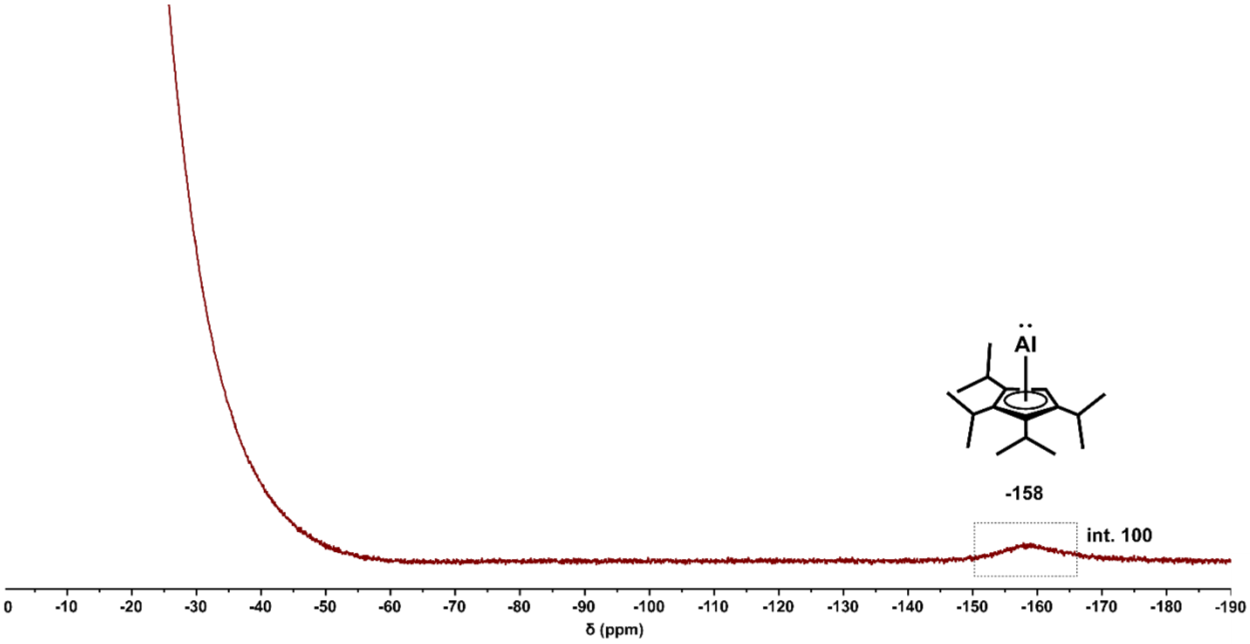

Figure S4. ^{27}Al NMR of **AlCp^{iPr4}** at –40 °C where the relative integral of monomer to tetramer is 100:0.

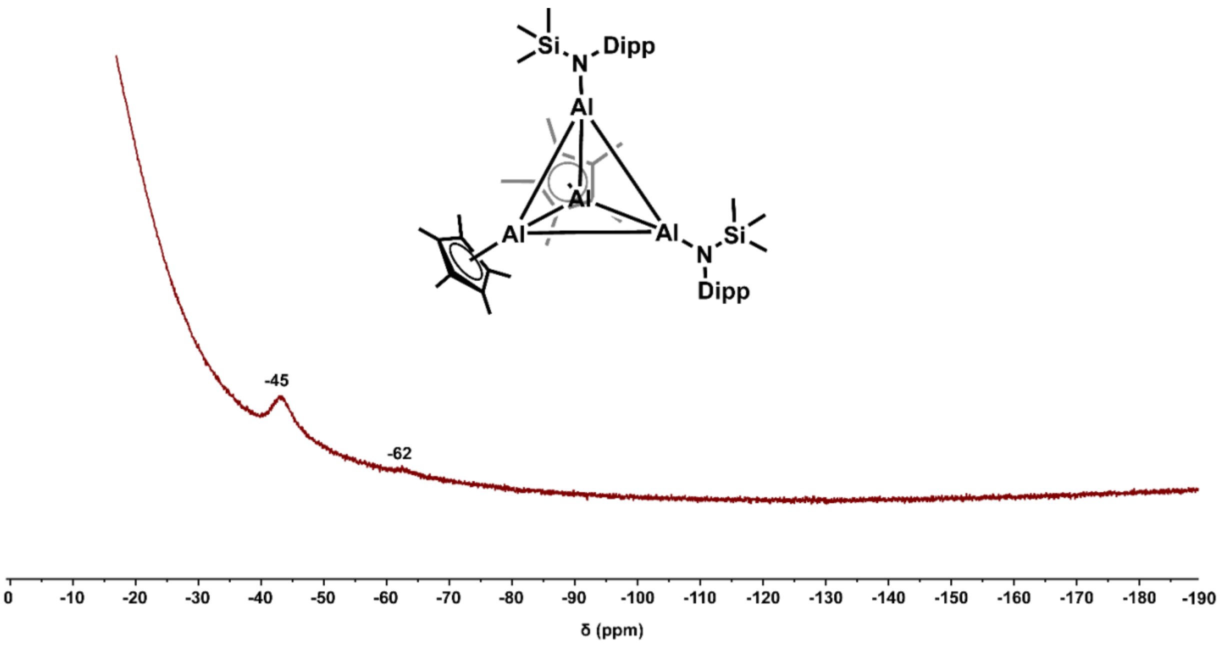

Figure S5. ^{27}Al NMR of **[AlNTMSDippAlCp*]$_2$·C$_6$H$_{14}$** at room temperature and the observed chemical shifts.

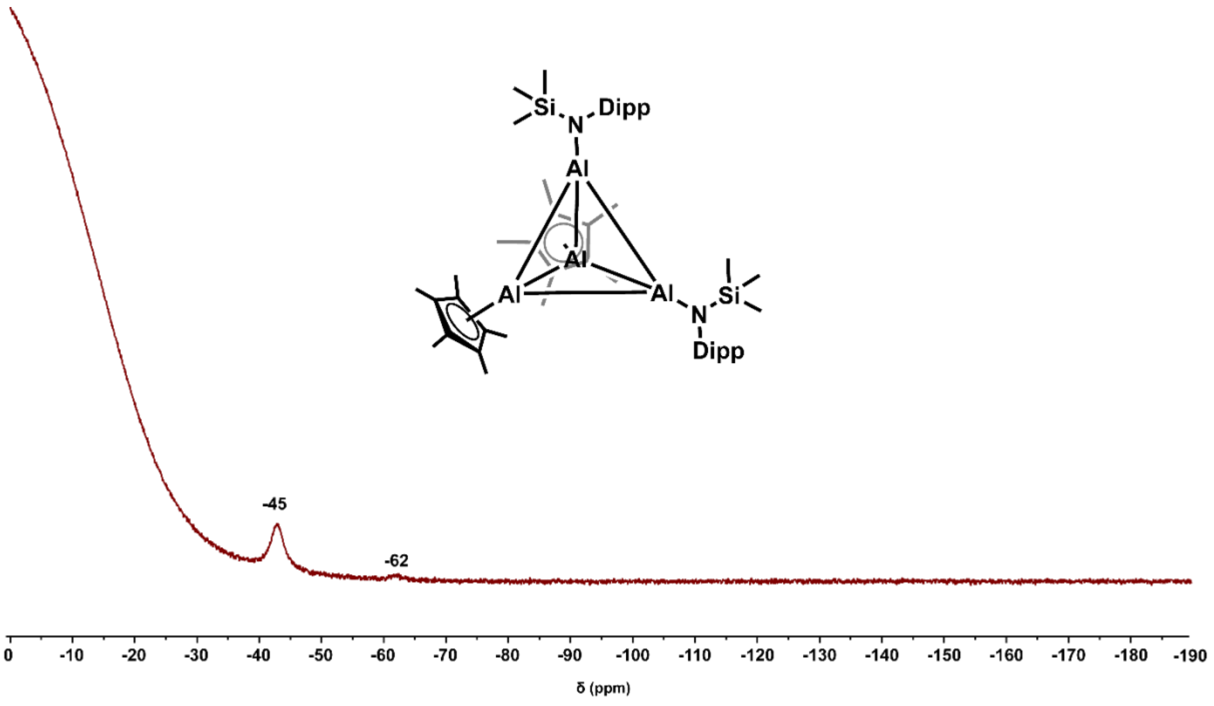

Figure S6. ^{27}Al NMR of **[AlNTMSDippAlCp*]$_2$·C$_6$H$_{14}$** at 65 °C and the observed chemical shifts.

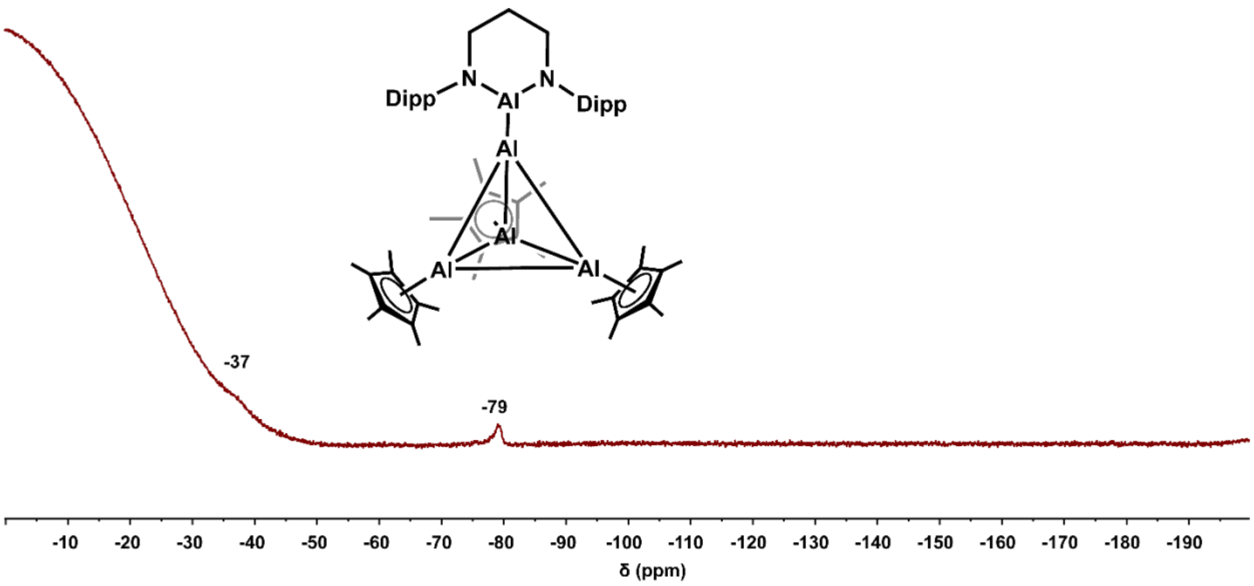

Figure S7. ^{27}Al NMR of **[Al(N$_2$Dipp$_2$C$_3$H$_6$)(Al$_4$Cp$^*_3$)]·0.5C$_6$H$_6$** at room temperature and the observed chemical shifts.

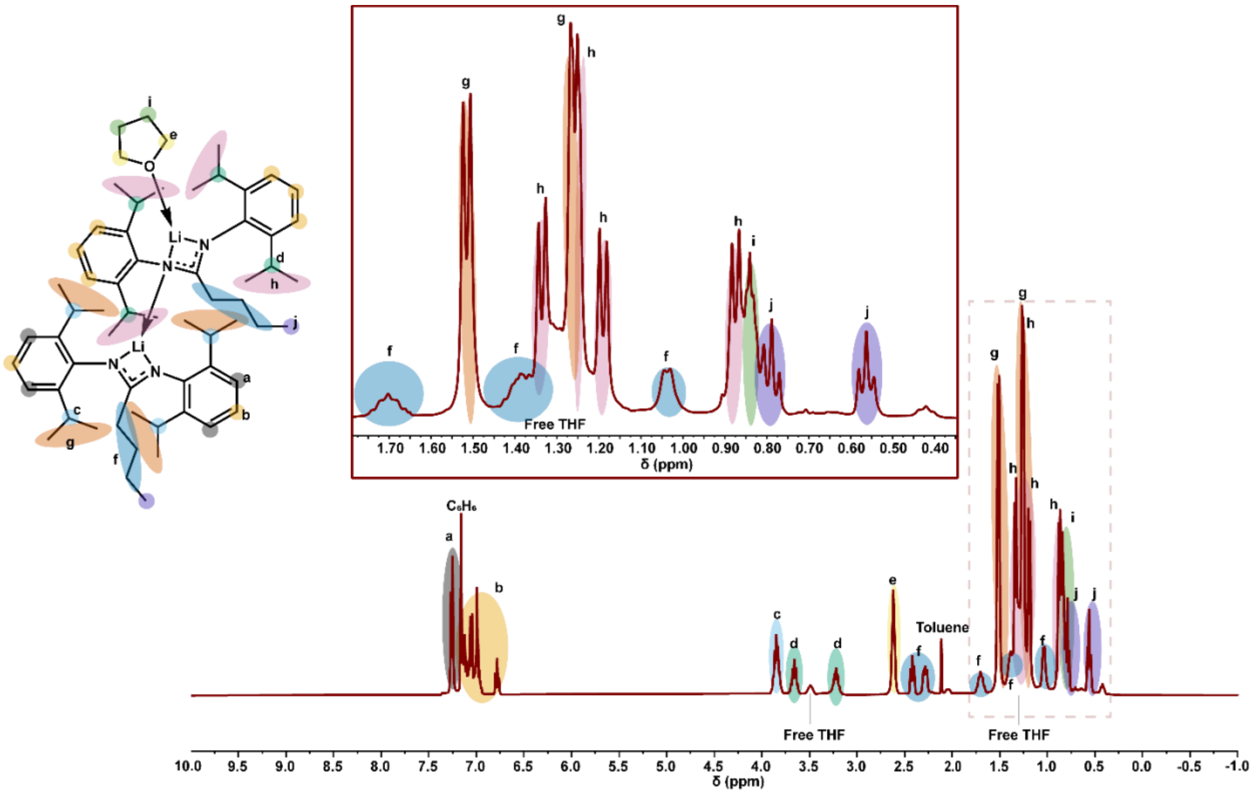

Figure S8. ^{1}H NMR spectra of **[(Li(NDipp)$_2$C$_5$H$_9$)$_2$THF]**. ^{1}H NMR assignments of chemical environments are indicated by color/letter matching. The following integrals are referenced to the first doublet g (1.52 ppm) worth 12 protons: a,b – 13.4 (12H), c,d – 8.2 (8H), e – 4.6 (4H), f (isolated) – 7.98 (8H) g,h – 45.6 (48H), i – 4.2 (4H), j – 5.5 (6H).

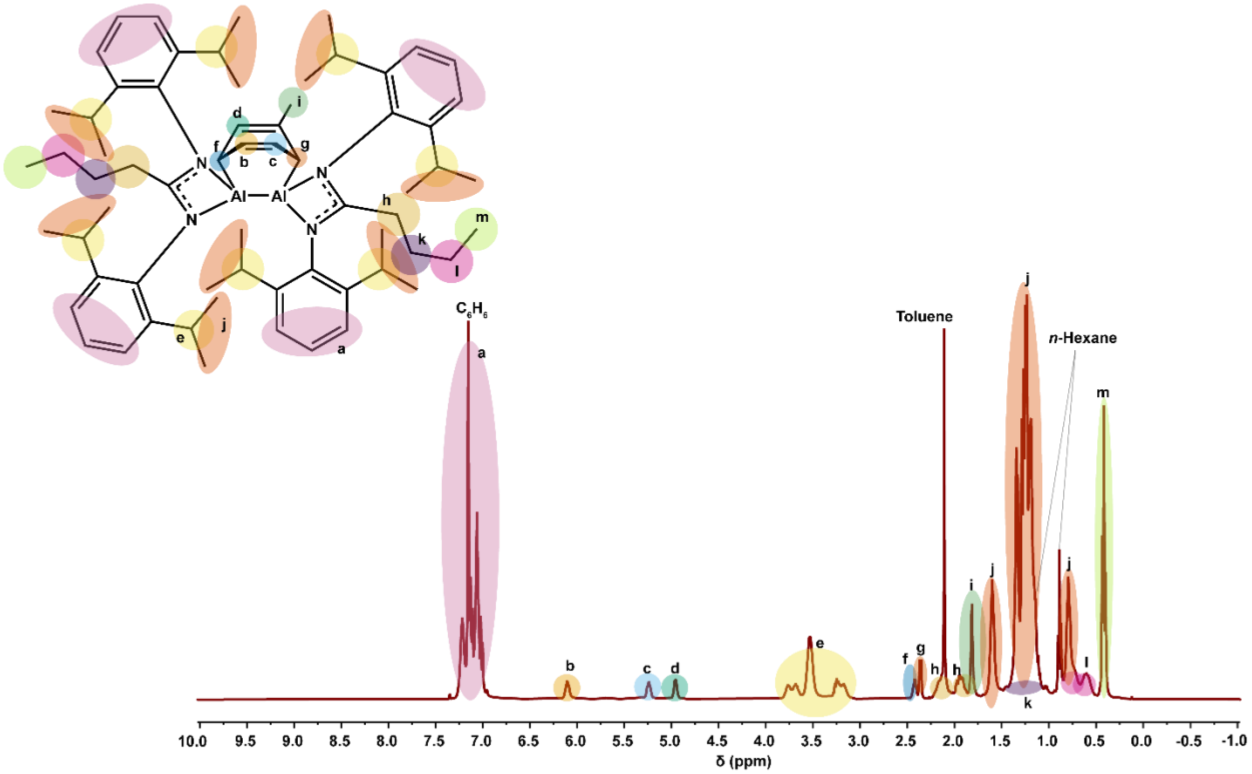

Figure S9. ^{1}H NMR spectra of **[(Al(NDipp)$_2$C$_5$H$_9$)$_2$C$_7$H$_8$]·C$_7$H$_8$**. ^{1}H NMR assignments of chemical environments are indicated by color/letter matching. The following integrals are referenced to the sets of septets (e) worth 8 protons: a – 9.9 (12H), b – 0.9 (1H), c – 0.8 (1H), d – 0.8 (1H), e – 8.0 (8H), f,g – 1.8 (2H), h – 3.3 (4H), i – 2.5 (3), j,k,l – 63.8 (56H), m – 7.1 (6H).

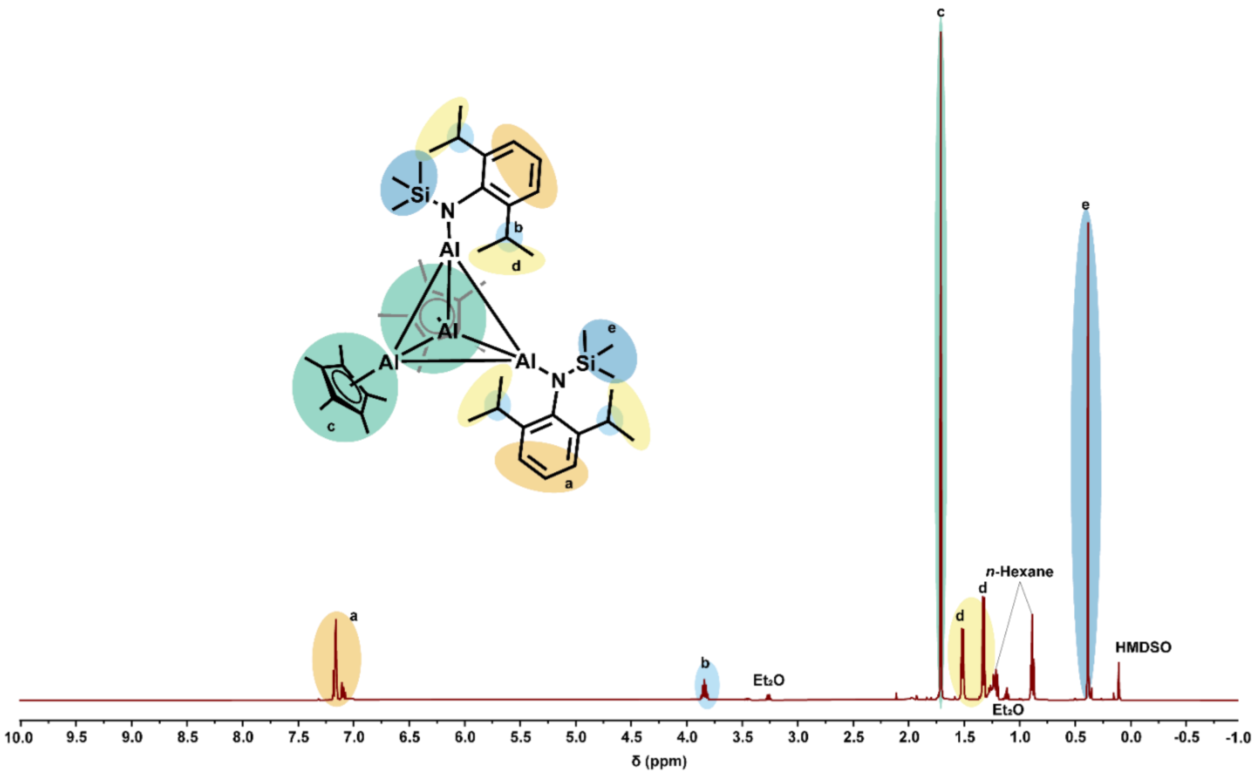

Figure S10. ^{1}H NMR spectra of **[AlNTMSDippAlCp*]$_2$·C_6H_{14}**. ^{1}H NMR assignments of chemical environments are indicated by color/letter matching. The following integrals are referenced to the region c worth 30 protons: a – 6.0 (6H), b – 4.5 (4H), c – 30.0 (30H), d – 26.3 (24H), e – 19.0 (18H).

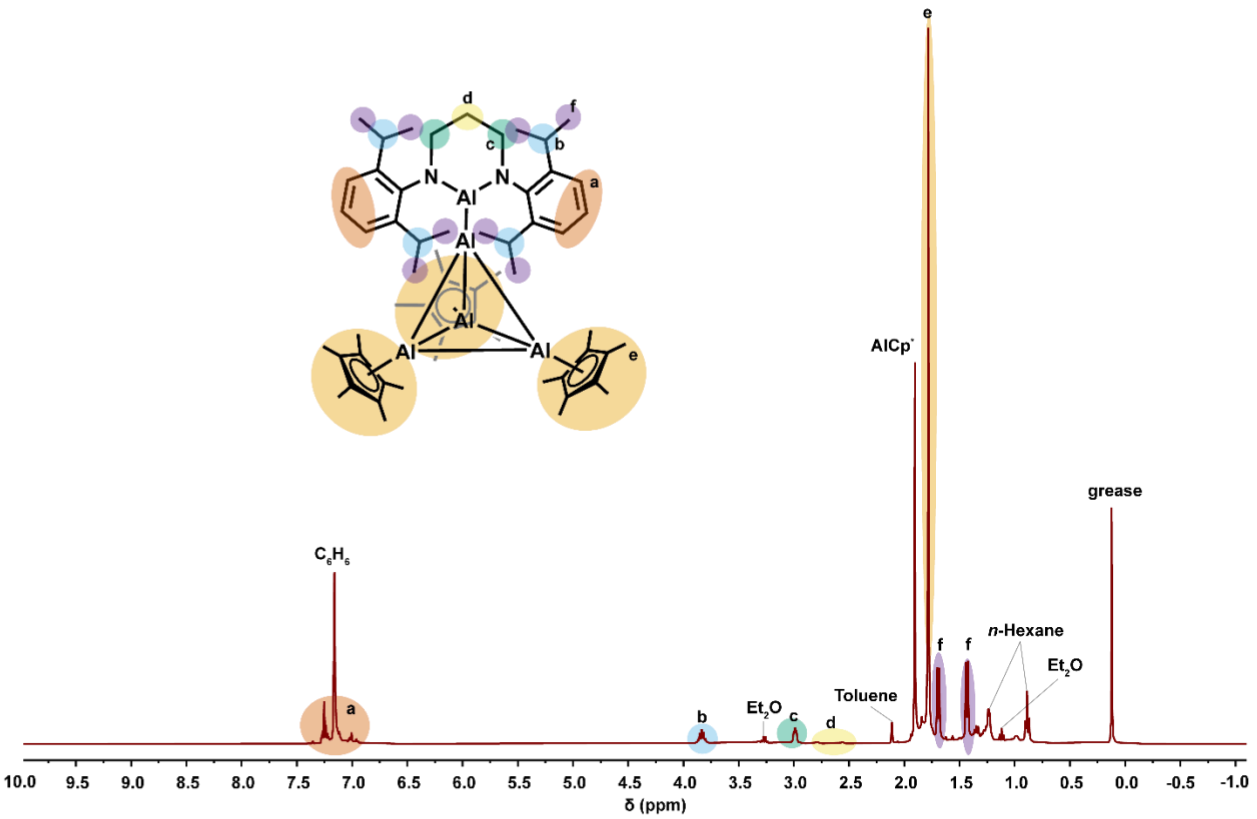

Figure S11. ^{1}H NMR spectra of **[Al($N_2Dipp_2C_3H_6$)($Al_4Cp^*_3$)]·0.5C_6H_6**. ^{1}H NMR assignments of chemical environments are indicated by color/letter matching. The following integrals are referenced to the region e worth 45 protons: a – 6.3 (6H), b – 3.6 (4H), c – 4.0 (4H), d – 0.9 (2H), e – 45 (45H), f – 25.4 (24H).

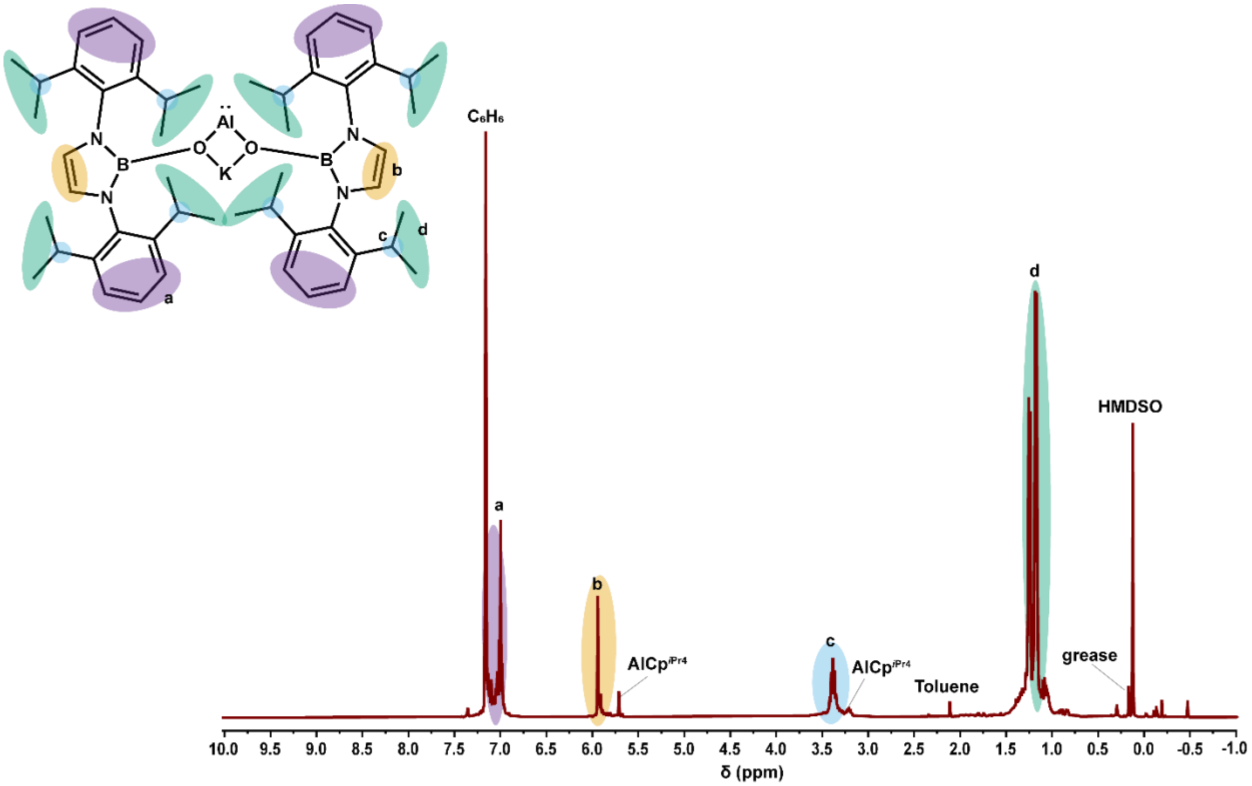

Figure S12. ^{1}H NMR spectra of **K[Al(OB(NDippCH)$_2$)$_2$]**. ^{1}H NMR assignments of chemical environments are indicated by color/letter matching. The following integrals are referenced to the sets of doublets (d) worth 48 protons: a – 11.3 (12H), b – 3.4 (4H), c – 6.4 (8H), d – 48.0 (48H).

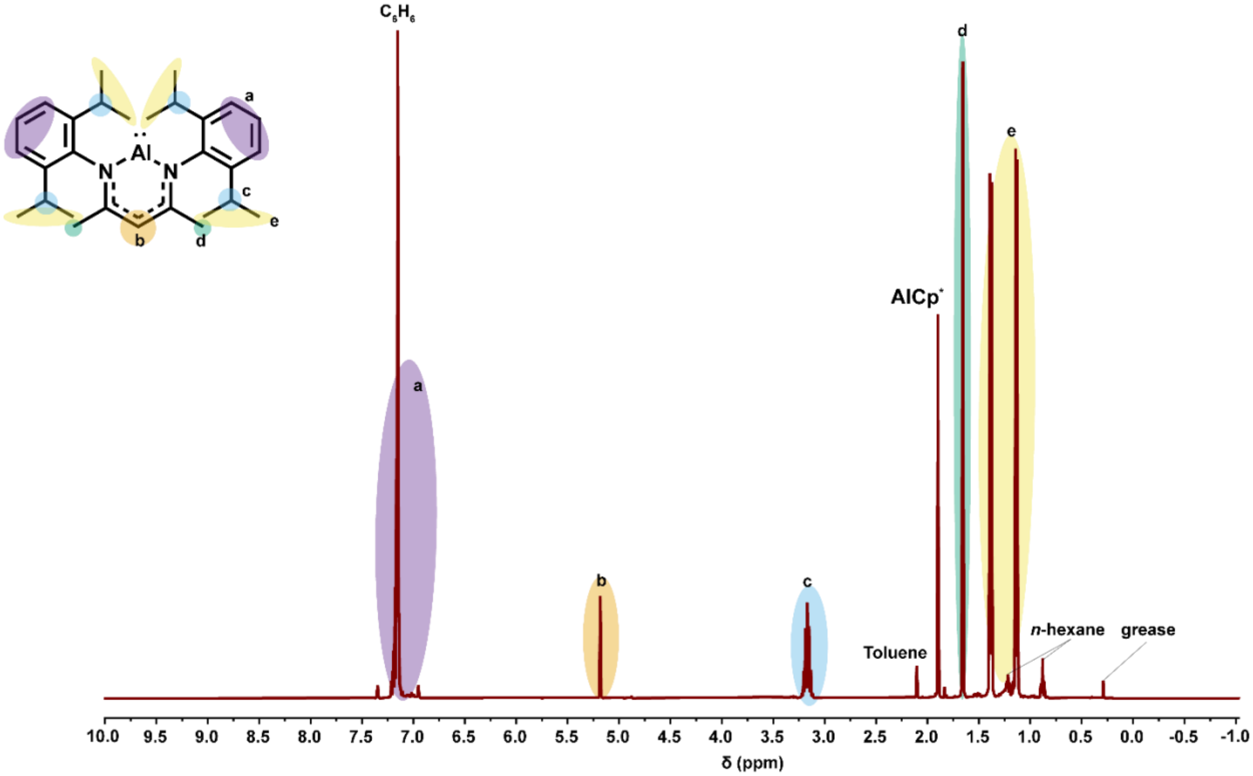

Figure S13. ^{1}H NMR spectra of **Al$^{\mathbf{Dipp}}$Nacnac**. ^{1}H NMR assignments of chemical environments are indicated by color/letter matching. The following integrals are referenced to the sets of doublets (e) worth 24 protons: a – 6.1 (6H), b – 0.8 (1H), c – 3.3 (4H), d – 5.5 (6H), e – 24.0 (24H).

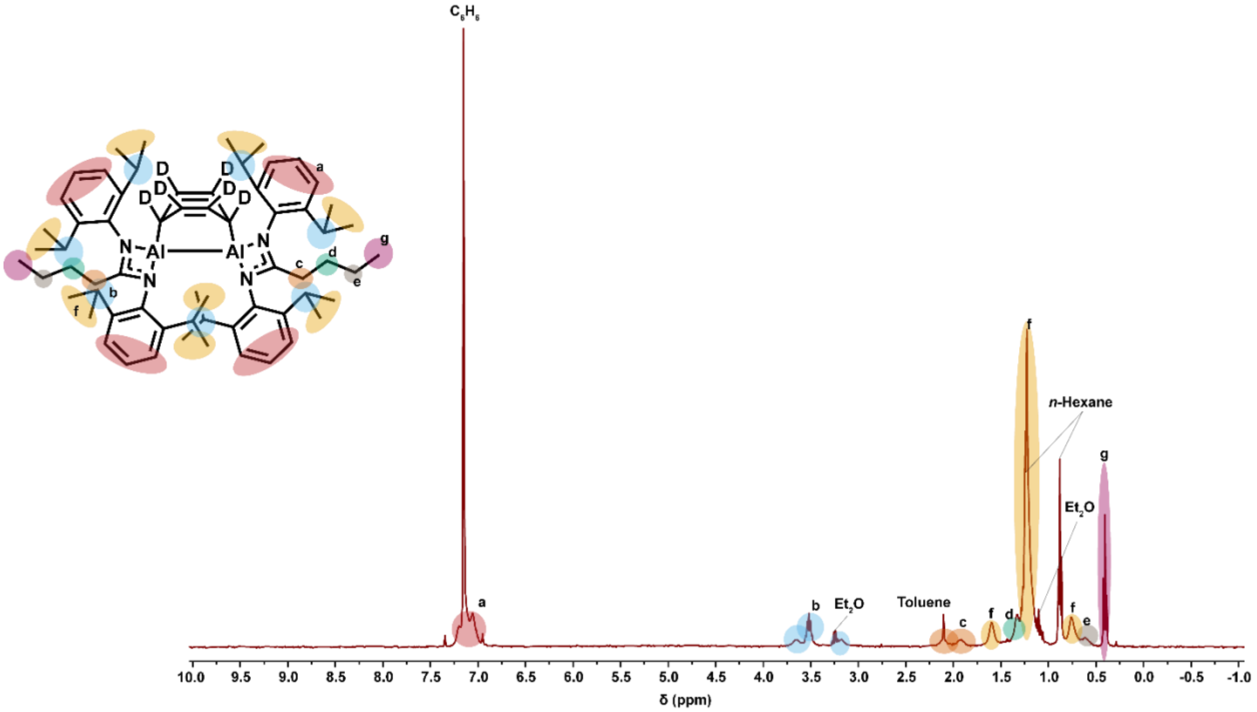

Figure S14. ^{1}H NMR spectra of **[(Al(NDipp)$_2$C$_5$H$_9$)$_2$C$_6$D$_6$]·C$_6$H$_{14}$**. ^{1}H NMR assignments of chemical environments are indicated by color/letter matching. The following integrals are referenced to the region containing d,f (1.41 ppm – 1.13 ppm) worth 40 protons: a – 14.3 (12H), b – 12.2 (8H), c – 2.9 (4H), d,f – 53.5 (52H), e – 3.8 (4H), g – 6.7 (6H).

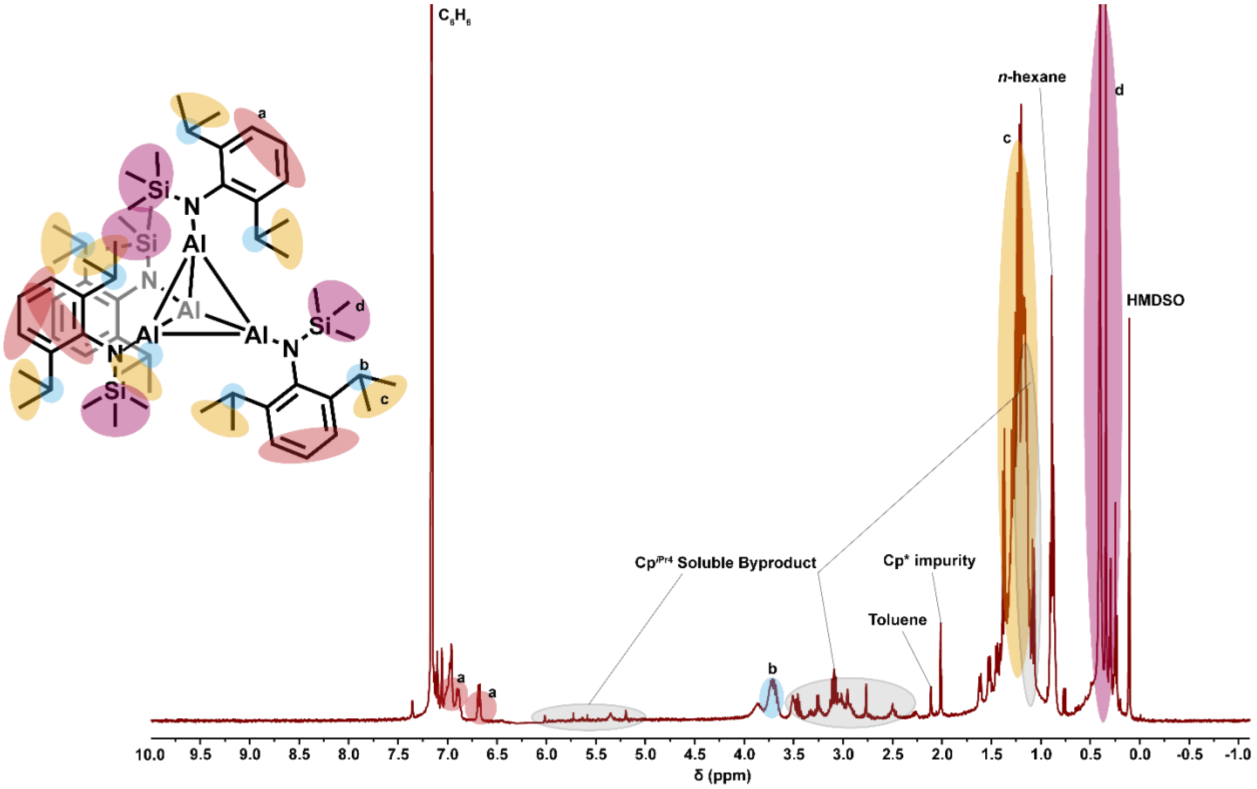

Figure S15. ^{1}H NMR spectra of **$[AlN(SiMe_3)(Dipp)]_4$**. ^{1}H NMR assignments of chemical environments are indicated by color/letter matching. Literature values (ppm) for same structure in different solvent: a – 7.15 (observed 7.0-6.6), b – 3.68 (observed 3.69), c – 1.24, 1.10 (observed 1.39-1.08), d – 0.21 (observed 0.40-0.33).

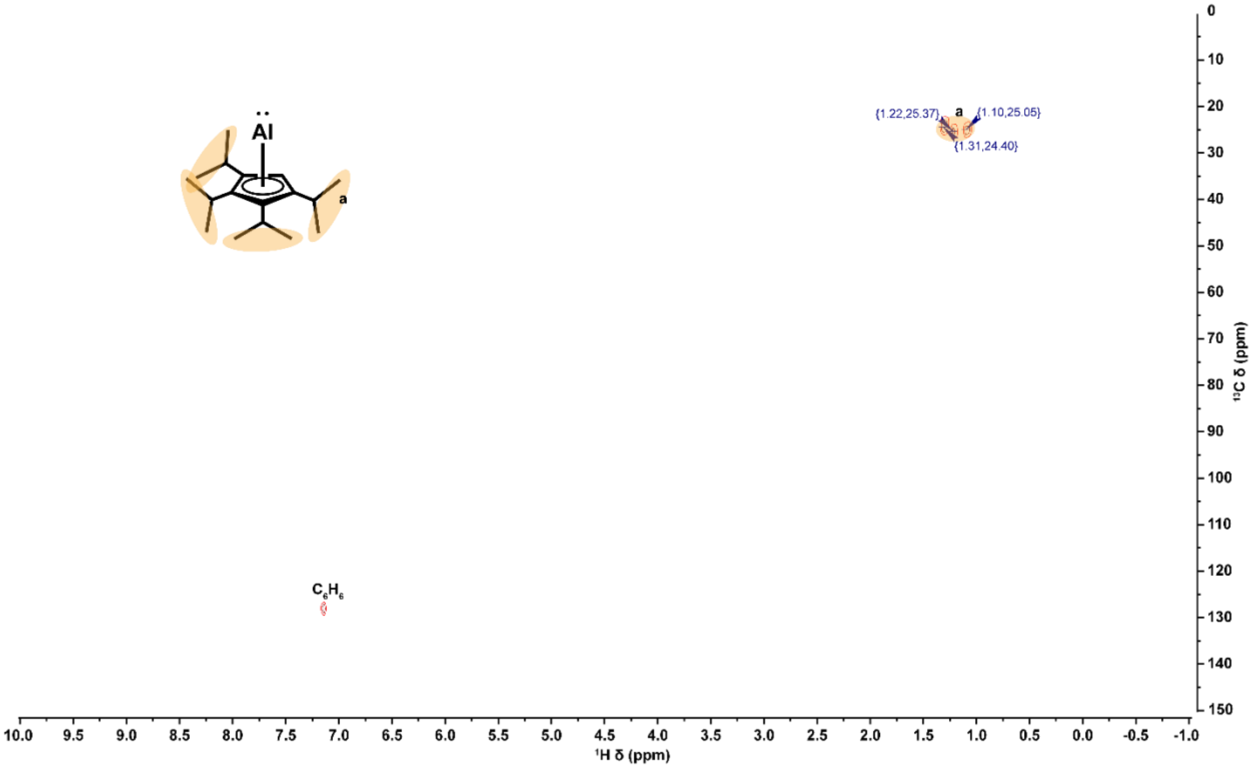

Figure S16. HSQC 2-D correlation spectra of **AlCp^{iPr4}**. Assignments of observed ^{1}H and ^{13}C NMR chemical shifts are indicated by color/letter.

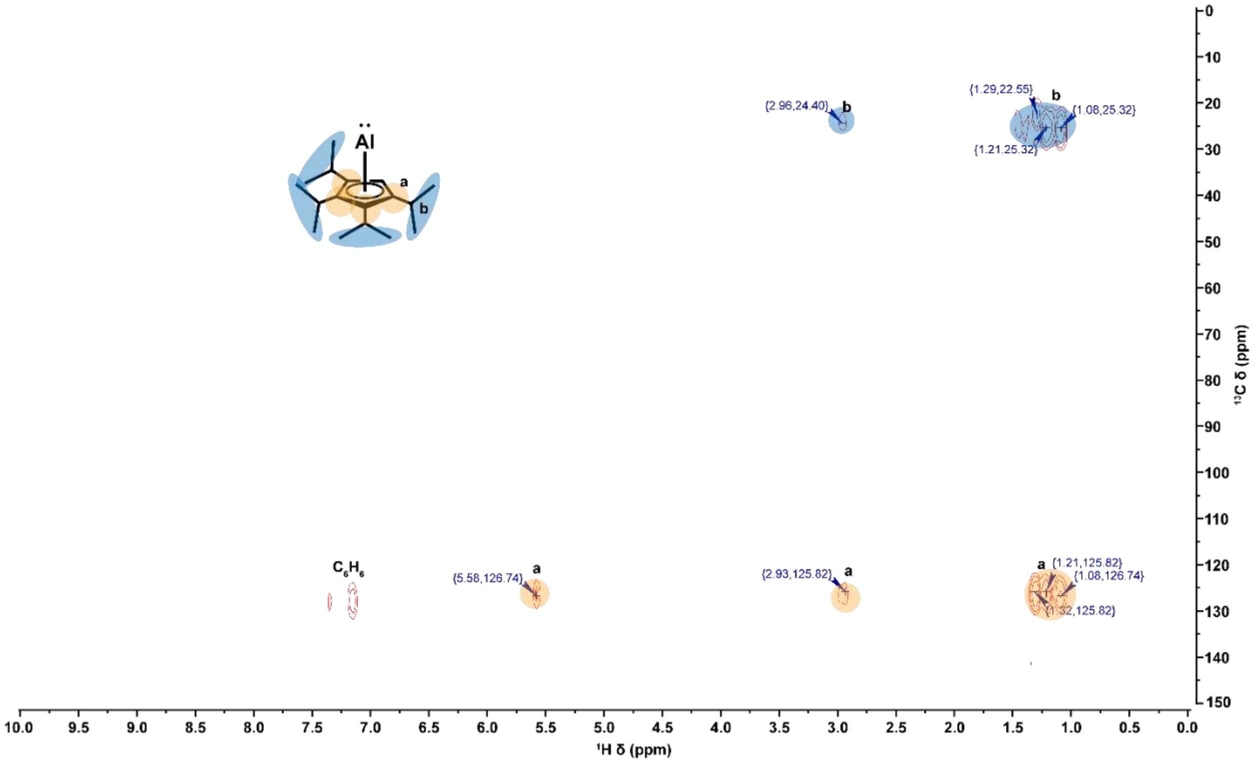

Figure S17. HMBC 2-D correlation spectra of **AlCp*iPr4***. Assignments of observed ^{1}H and ^{13}C NMR chemical shifts are indicated by color/letter.

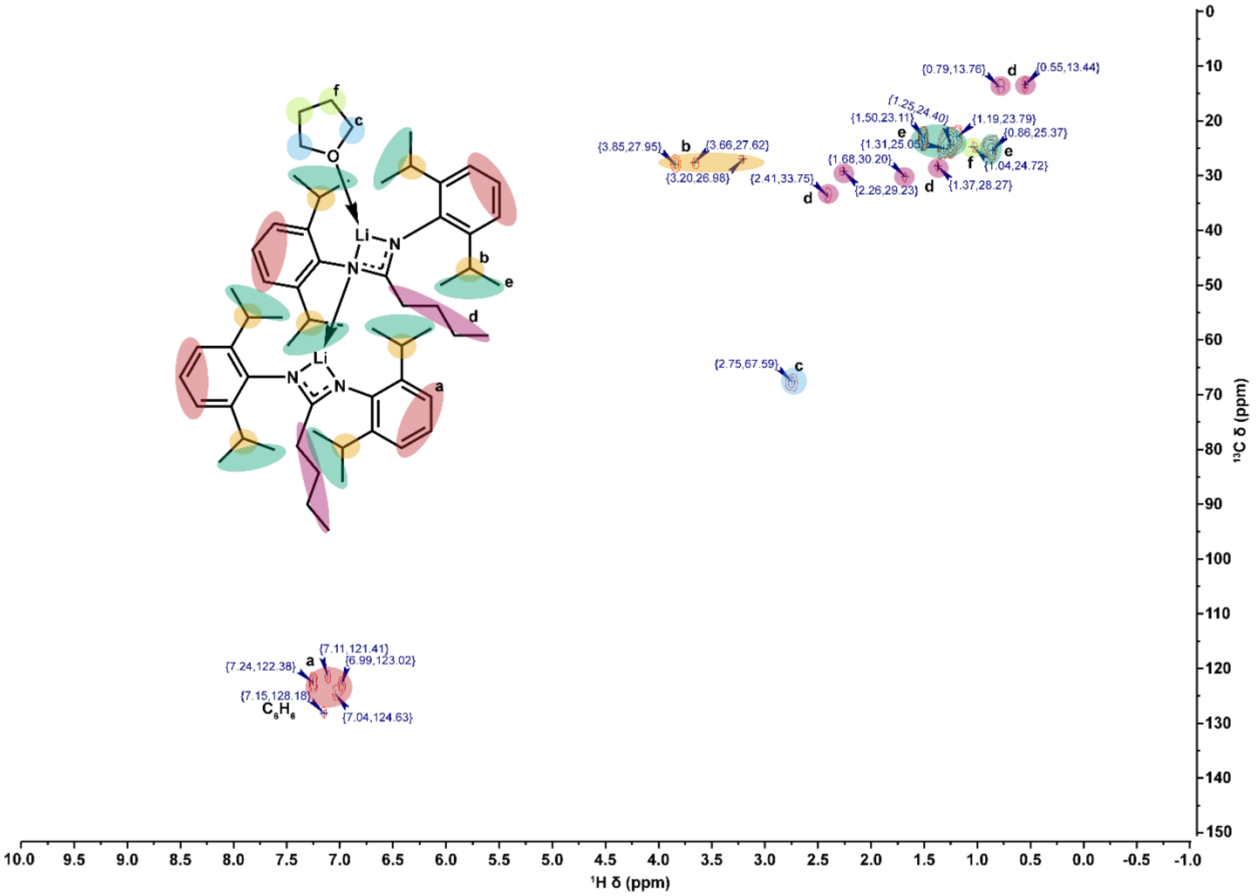

Figure S18. HSQC 2-D correlation spectra of **[(Li(NDipp)$_2$C$_5$H$_9$)$_2$THF]**. Assignments of observed ^{1}H and ^{13}C NMR chemical shifts are indicated by color/letter.

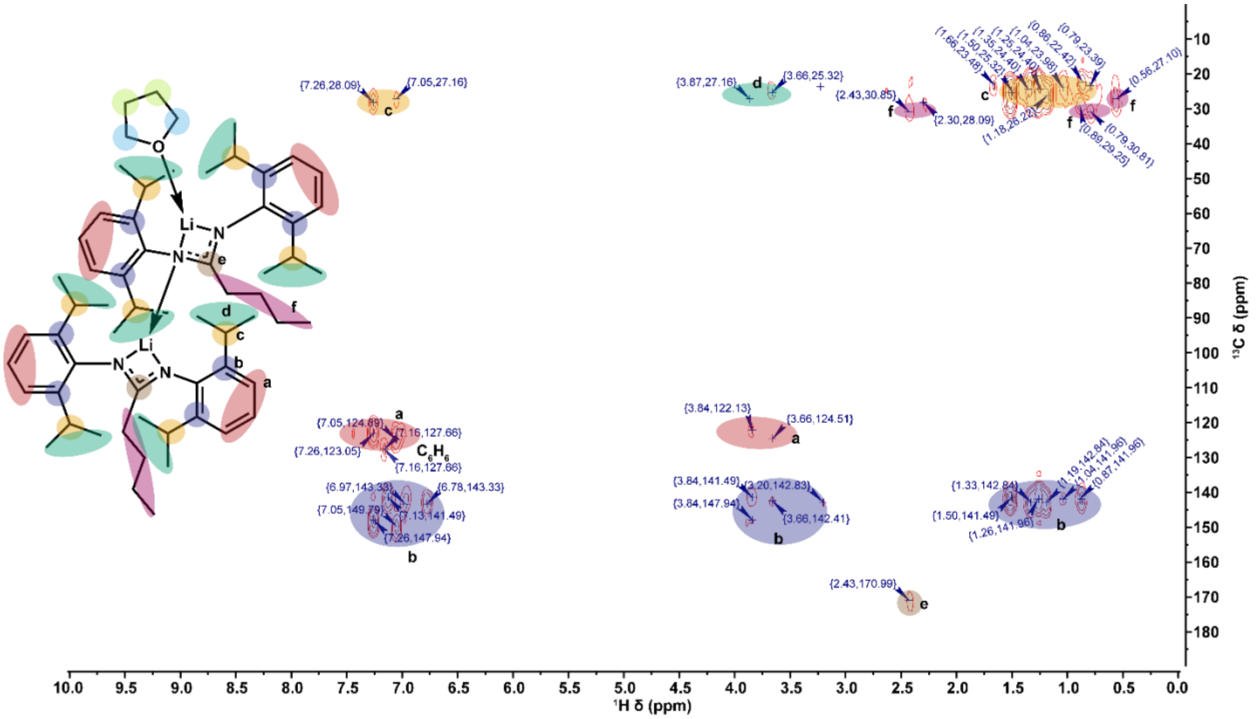

Figure S19. HMBC 2-D correlation spectra of **[(Li(NDipp)$_2$C$_5$H$_9$)$_2$THF]**. Assignments of observed ^{1}H and ^{13}C NMR chemical shifts are indicated by color/letter.

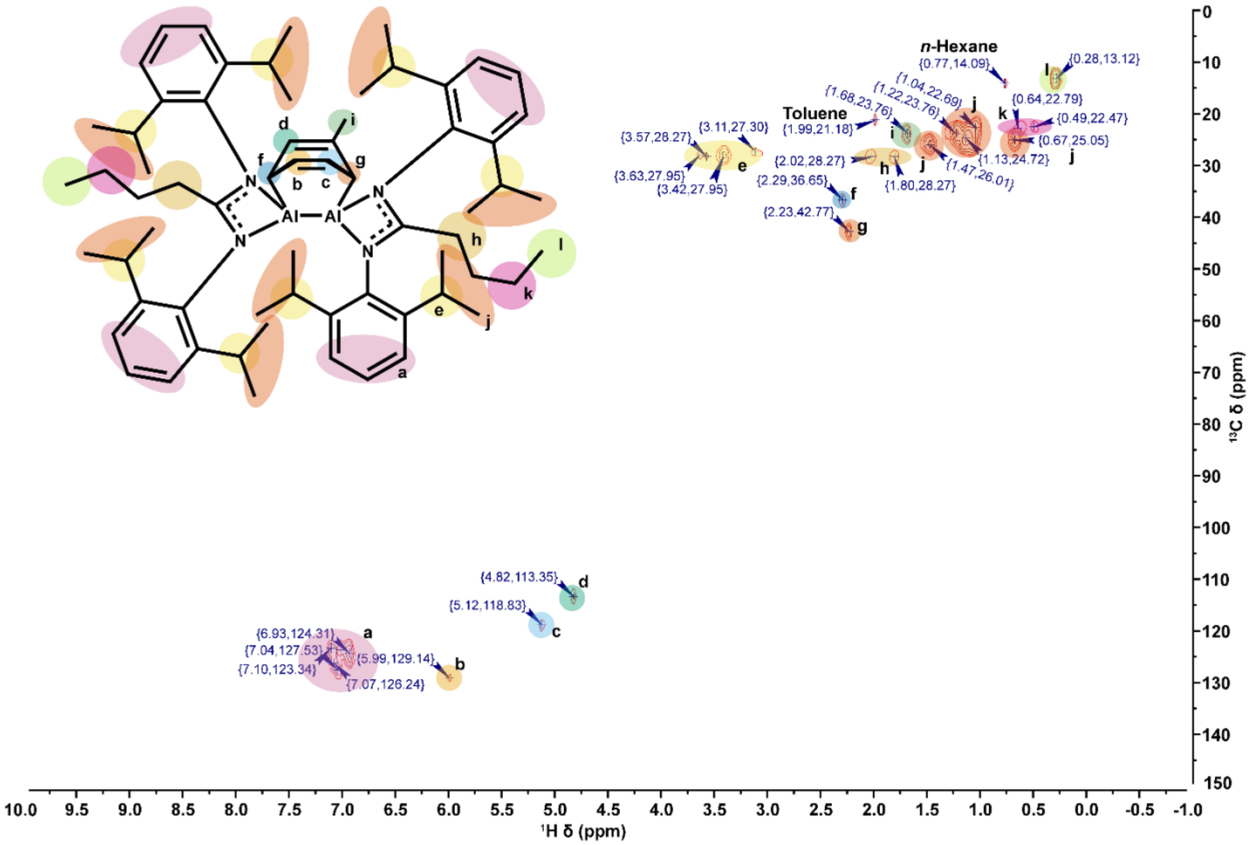

Figure S20. HSQC 2-D correlation spectra of **[(Al(NDipp)$_2$C$_5$H$_9$)$_2$C$_7$H$_8$]·C$_7$H$_8$**. Assignments of observed ^{1}H and ^{13}C NMR chemical shifts are indicated by color/letter.

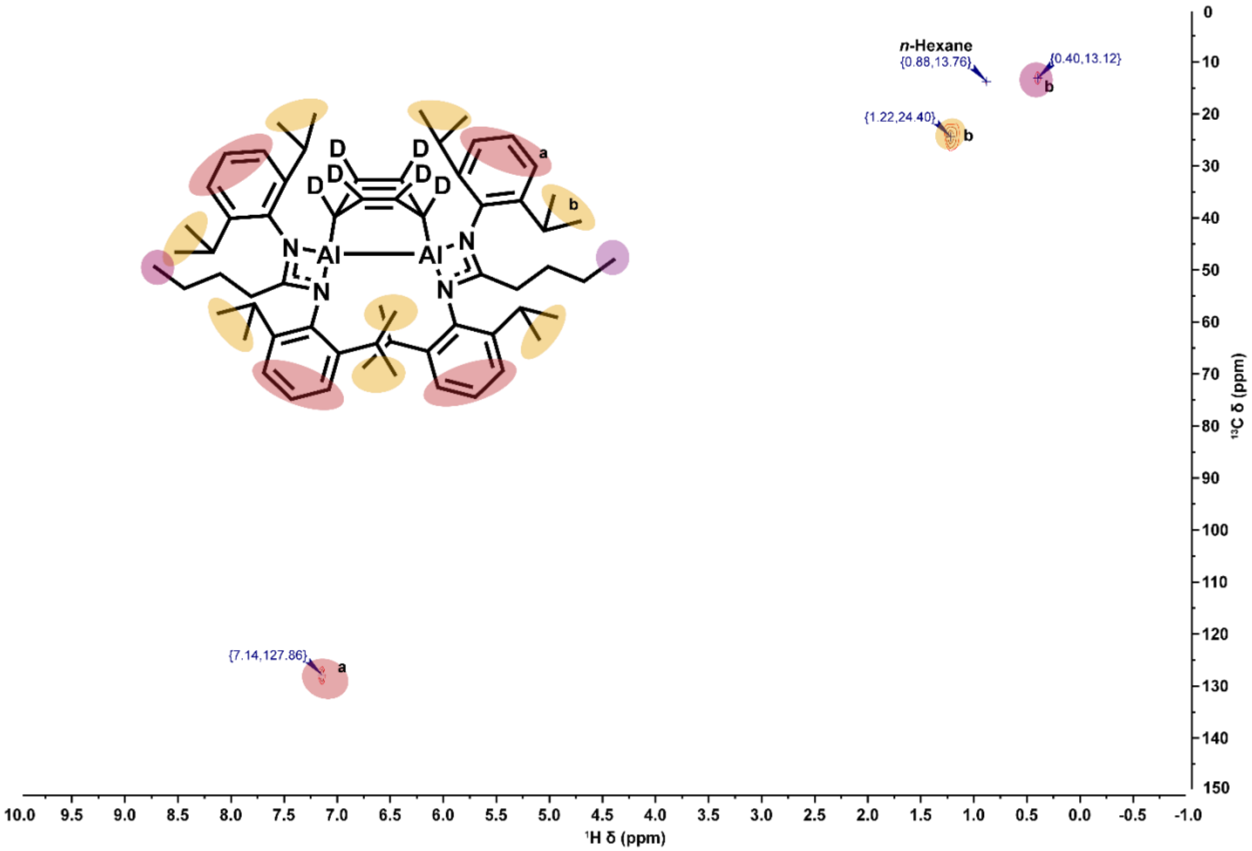

Figure S21. HSQC 2-D correlation spectra of **[(Al(NDipp)$_2$C$_5$H$_9$)$_2$C$_6$D$_6$]·C$_6$H$_{14}$**. Assignments of observed ^{1}H and ^{13}C NMR chemical shifts are indicated by color/letter.

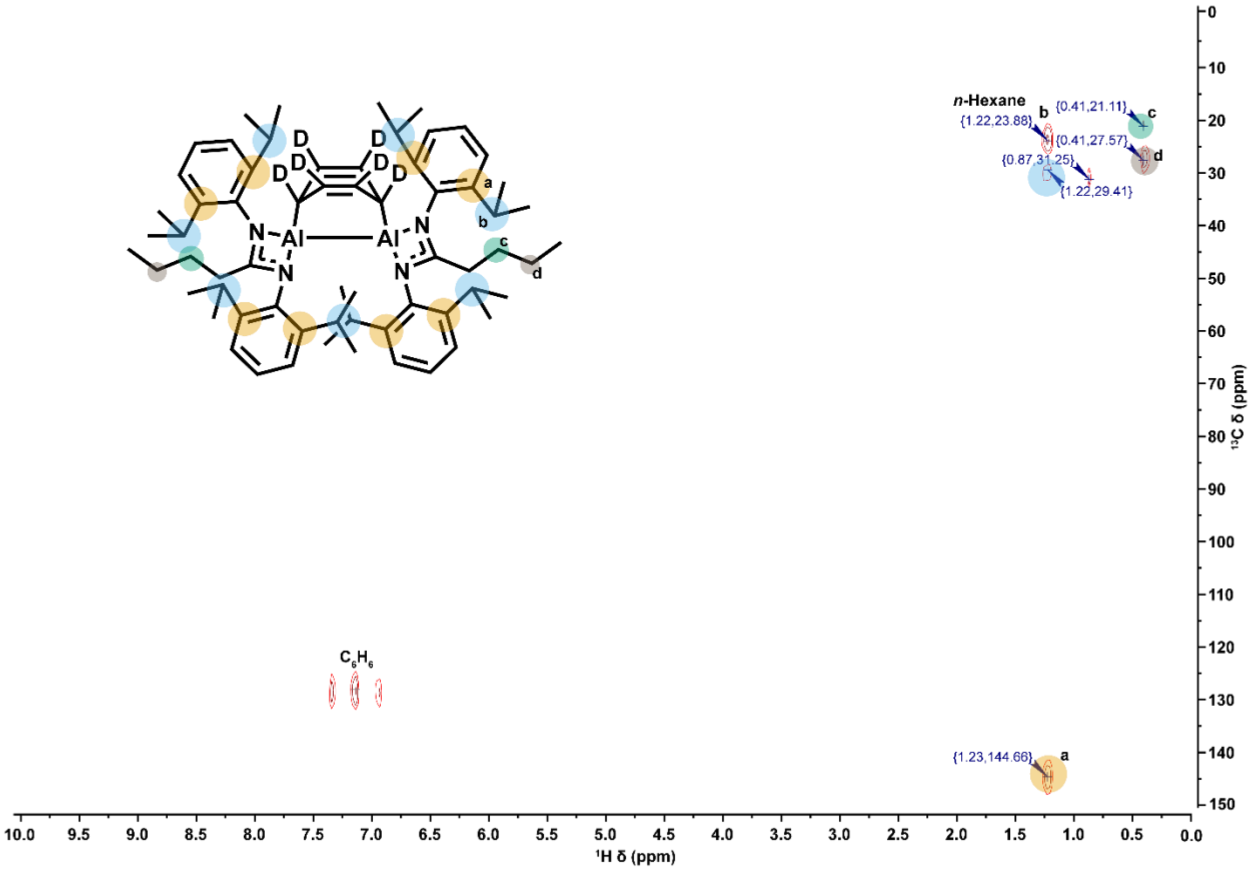

Figure S22. HMBC 2-D correlation spectra of **[(Al(NDipp)$_2$C$_5$H$_9$)$_2$C$_6$D$_6$]·C$_6$H$_{14}$**. Assignments of observed ^{1}H and ^{13}C NMR chemical shifts are indicated by color/letter.

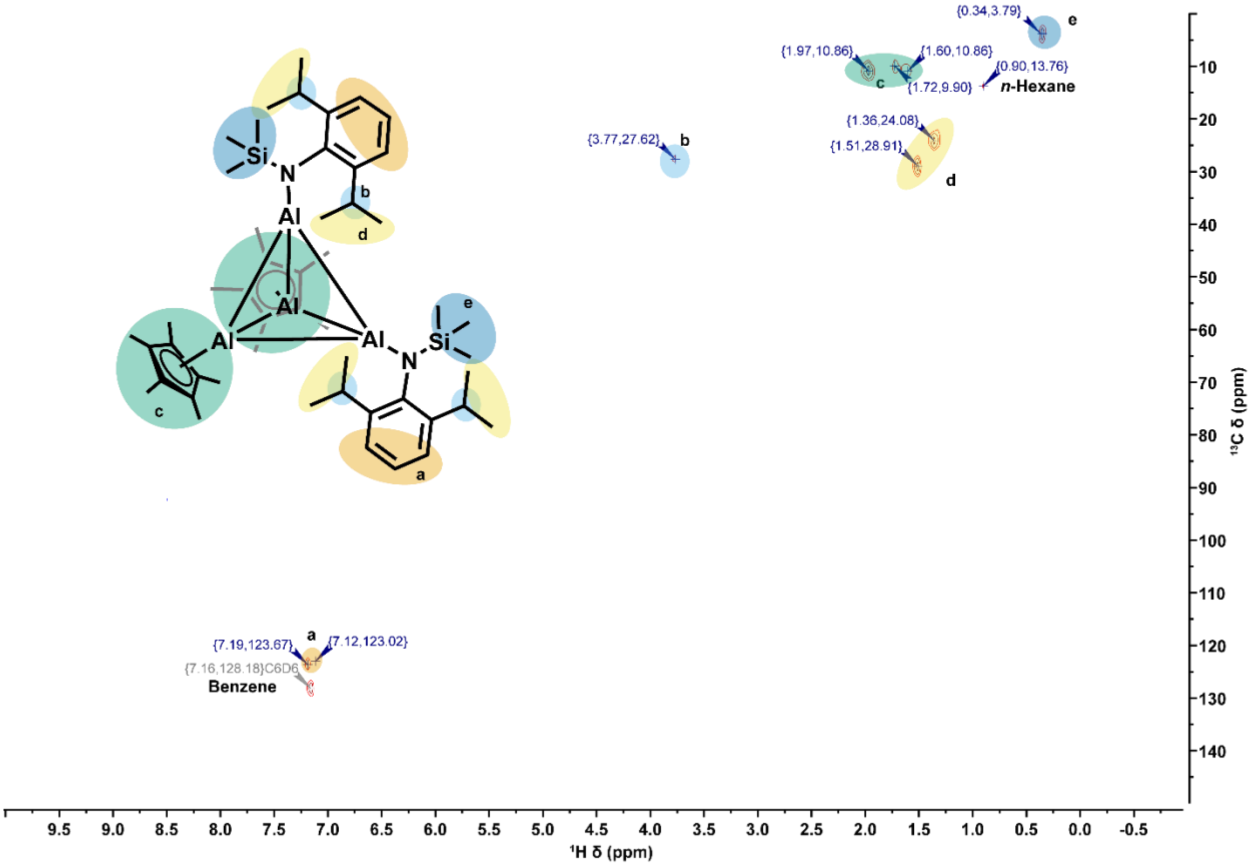

Figure S23. HSQC 2-D correlation spectra of **[AlNTMSDippAlCp*]$_2$·C$_6$H$_{14}$**. Assignments of observed ^{1}H and ^{13}C NMR chemical shifts are indicated by color/letter.

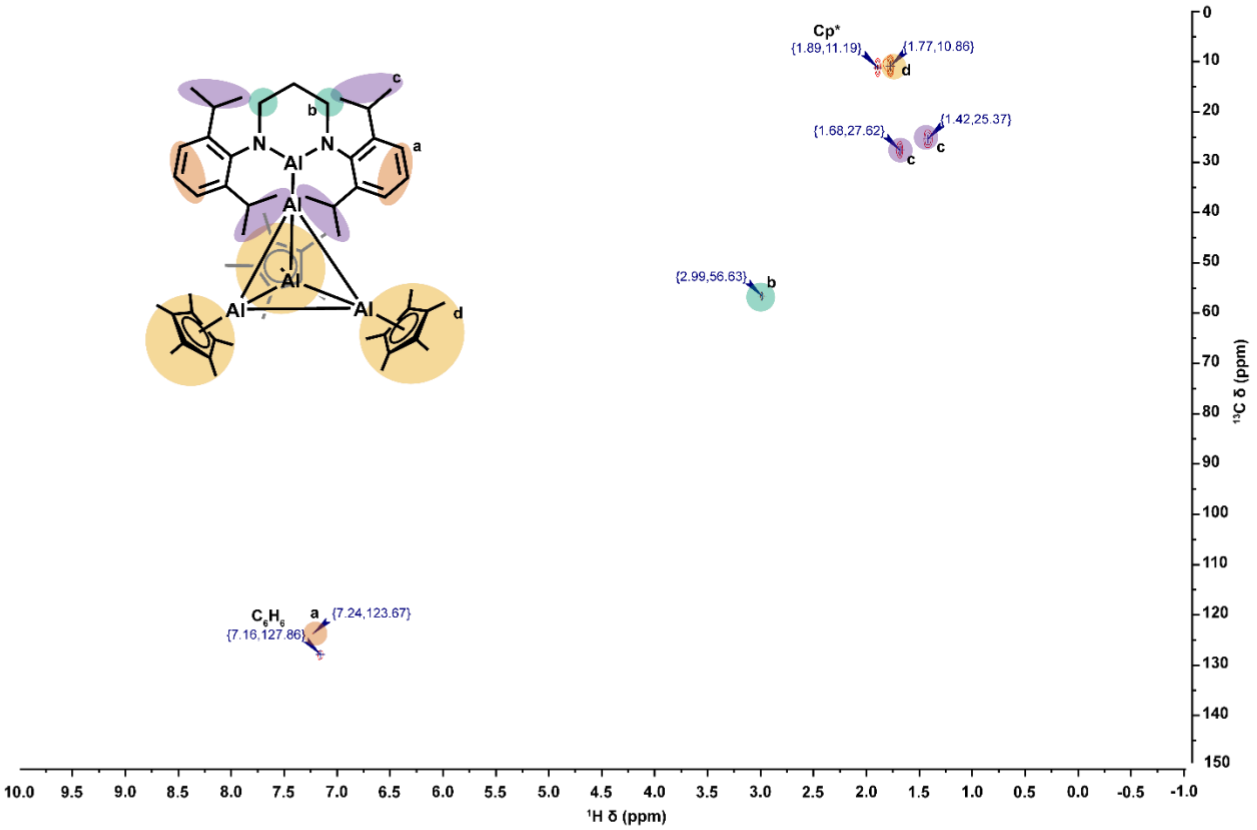

Figure S24. HSQC 2-D correlation spectra of **[Al(N_2Dipp$_2$$C_3H_6$)($Al_4Cp^*_3$)]·0.5$C_6H_6$**. Assignments of observed ^{1}H and ^{13}C NMR chemical shifts are indicated by color/letter.

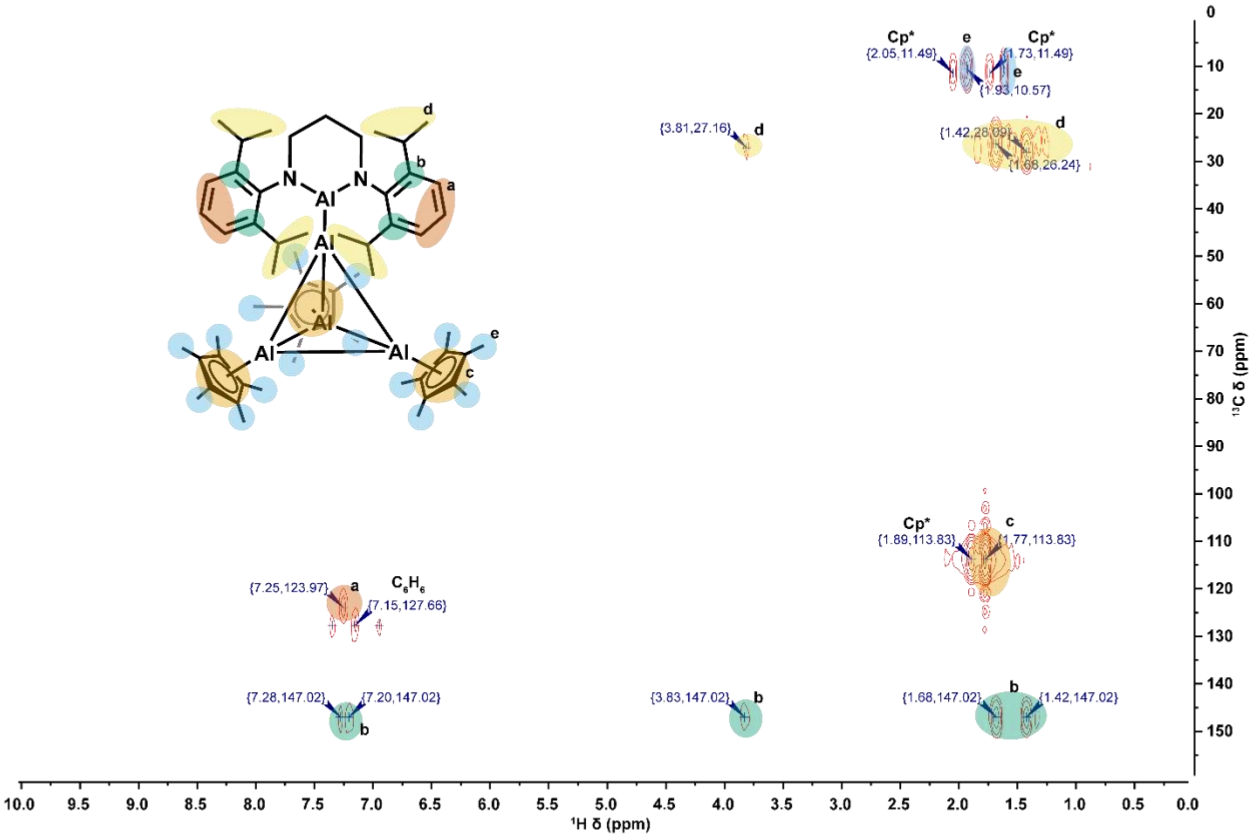

Figure S25. HMBC 2-D correlation spectra of **[Al(N_2Dipp$_2$$C_3H_6$)($Al_4Cp^*_3$)]·0.5$C_6H_6$**. Assignments of observed ^{1}H and ^{13}C NMR chemical shifts are indicated by color/letter.

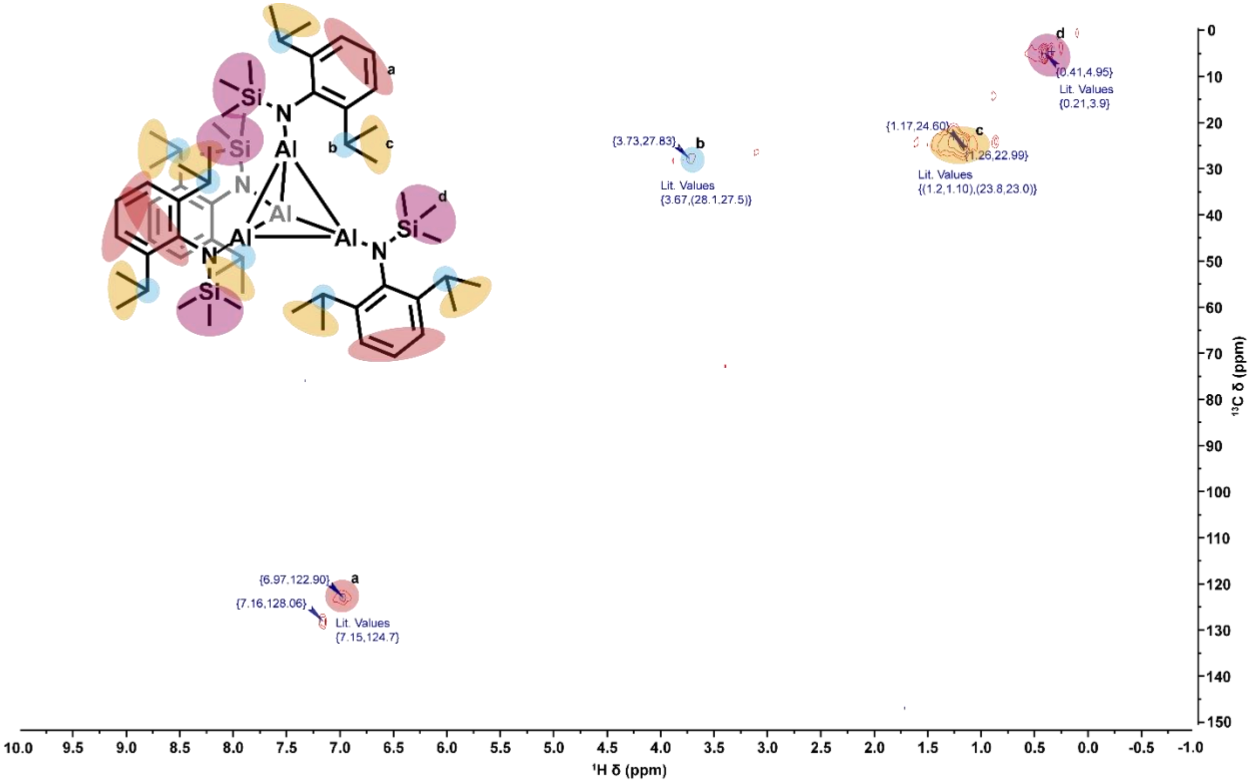

Figure S26. HSQC 2-D correlation spectra of **$[AlN(SiMe_3)(Dipp)]_4$**. Assignments of observed ^{1}H and ^{13}C NMR chemical shifts are indicated by color/letter. Literature values (in $^{i}Pr_2O/C_6D_6$ are correlated to observed signals[19].

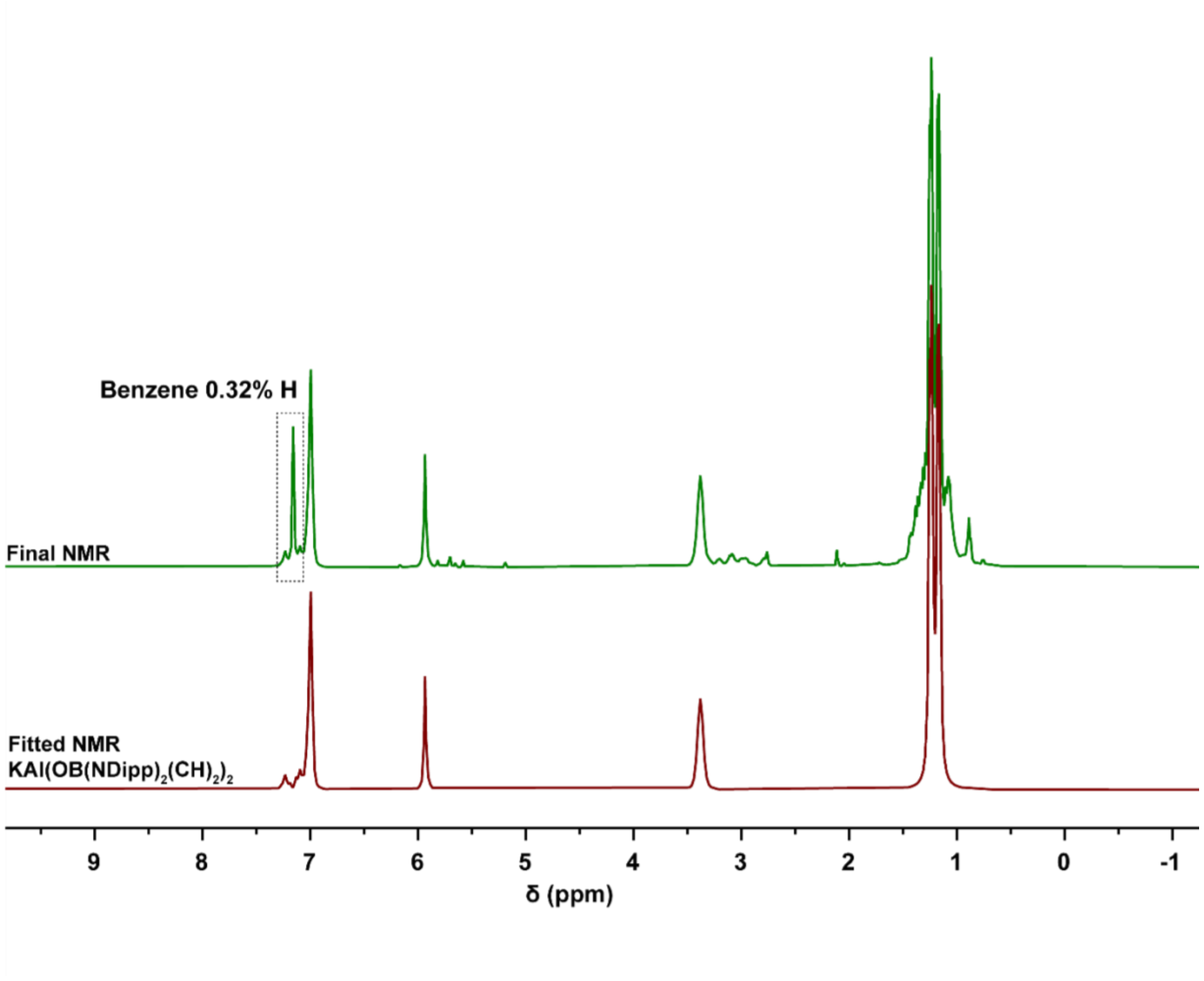

Figure S27. *In-situ* ^{1}H NMR spectrum from the synthesis of **K[Al(OB(NDippCH)₂)₂]**. *Top/green* – raw data from reaction of **AlCp^{iPr4}** and **K(OB(NDippCH)₂)** at room temperature after 30 minutes. *Bottom/red* – deconvolution and fit of peaks associated with **K[Al(OB(NDippCH)₂)₂]**.

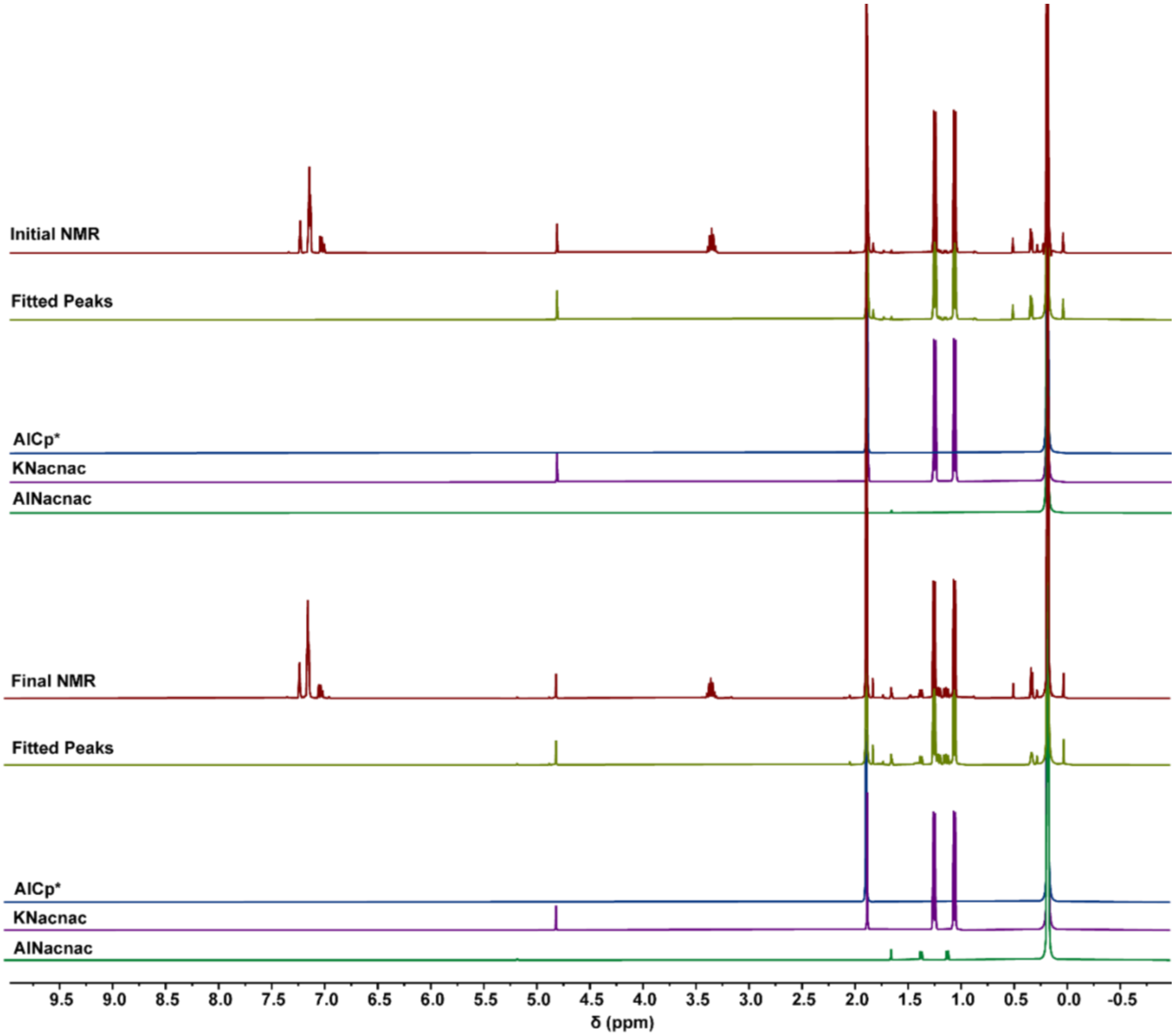

Figure S28. Deconvoluted ^{1}H NMR signals for the reagents and products of **AlCp*** and **K^{Dipp}Nacnac** to produce **Al^{Dipp}Nacnac**. The initial start time of the reaction was 0.5 h before the collection of the first spectrum (top). The last ^{1}H NMR spectrum (bottom) was collected 4 h later with heating at 60 °C. Quantitative yields are reported as the difference between these two spectra.

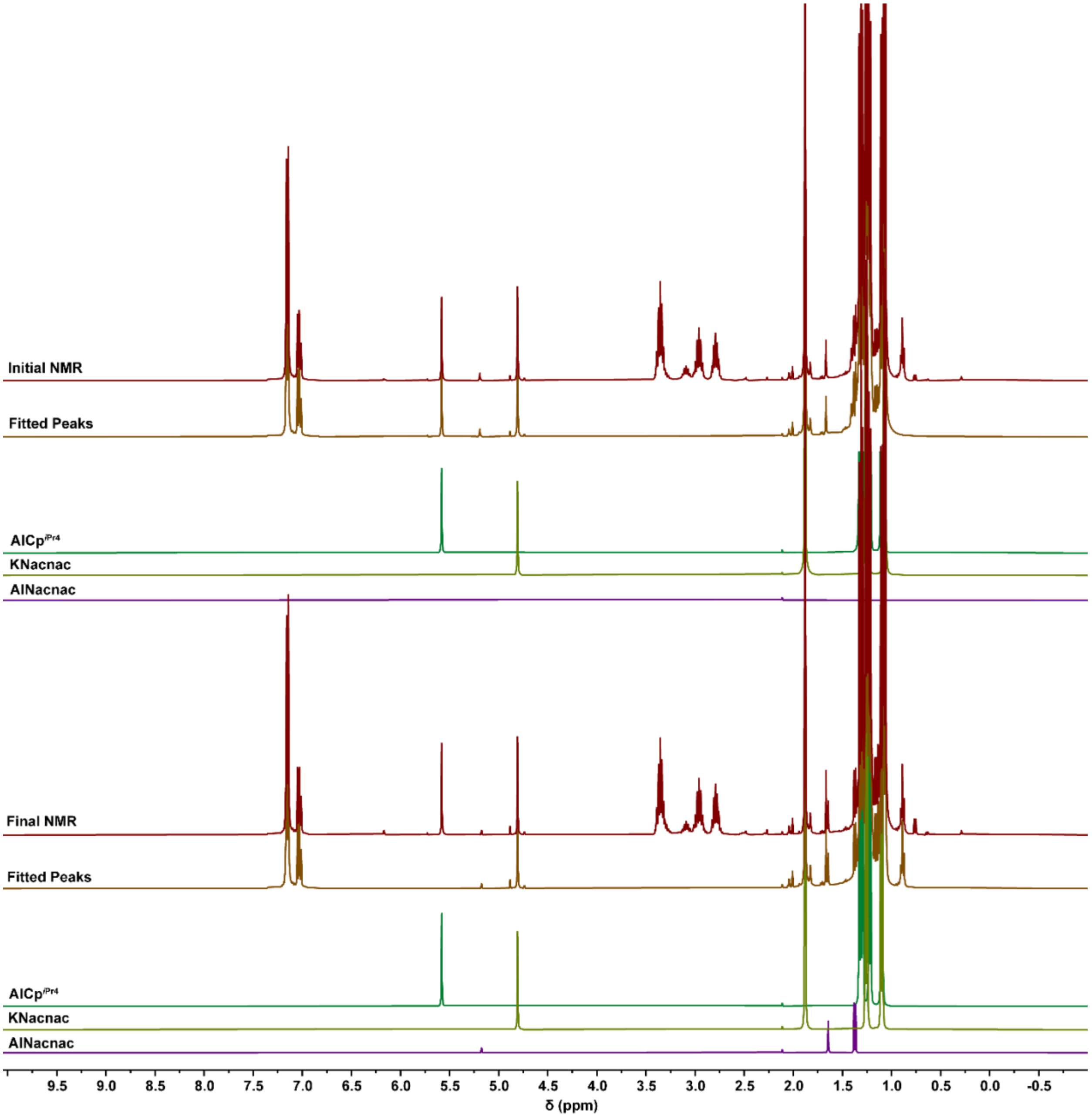

Figure S29. Deconvoluted ^{1}H NMR signals for the reagents and products of **AlCp^{iPr4}** and **K^{Dipp}Nacnac** to produce **Al^{Dipp}Nacnac**. The initial start time of the reaction was 0.5 h before the collection of the first spectrum (top). The last ^{1}H NMR spectrum (bottom) was collected after reacting for a total of 6 h of heating (2 h at 60 °C and 4 h at 70 °C). Quantitative yields are reported as the difference between these two spectra.

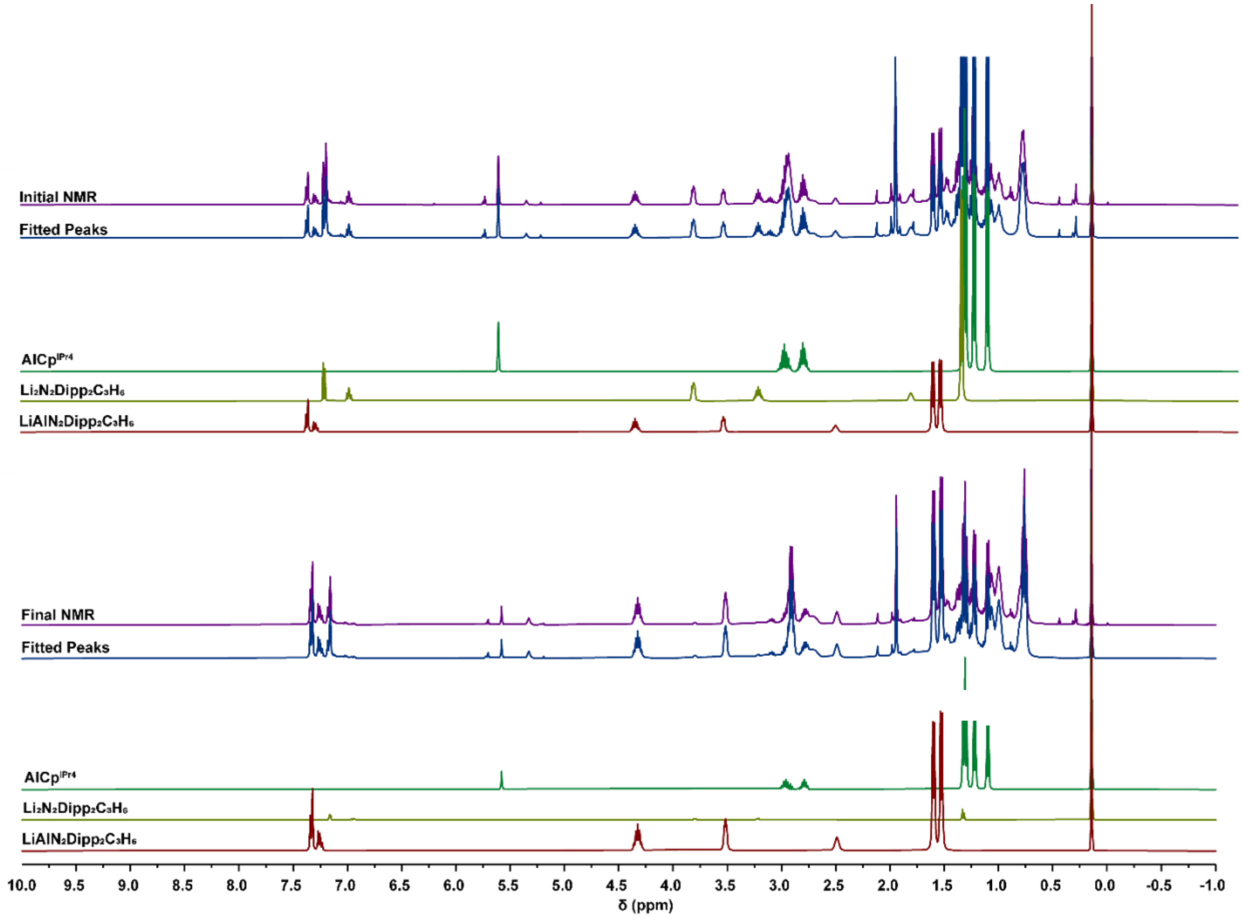

Figure S30. Deconvoluted ^{1}H NMR signals for the reagents and products of **AlCp^{iPr4}** and **LiAl(NDipp)$_2$(CH$_2$)$_3$** to produce **LiAl(NDipp)$_2$(CH$_2$)$_3$**. The initial start time of the reaction was 30 minutes before the collection of the first spectrum (top). The last ^{1}H NMR spectrum (bottom) was collected 12 h later. Quantitative yields are reported as the difference between these two spectra.

5. Single Crystal Structures

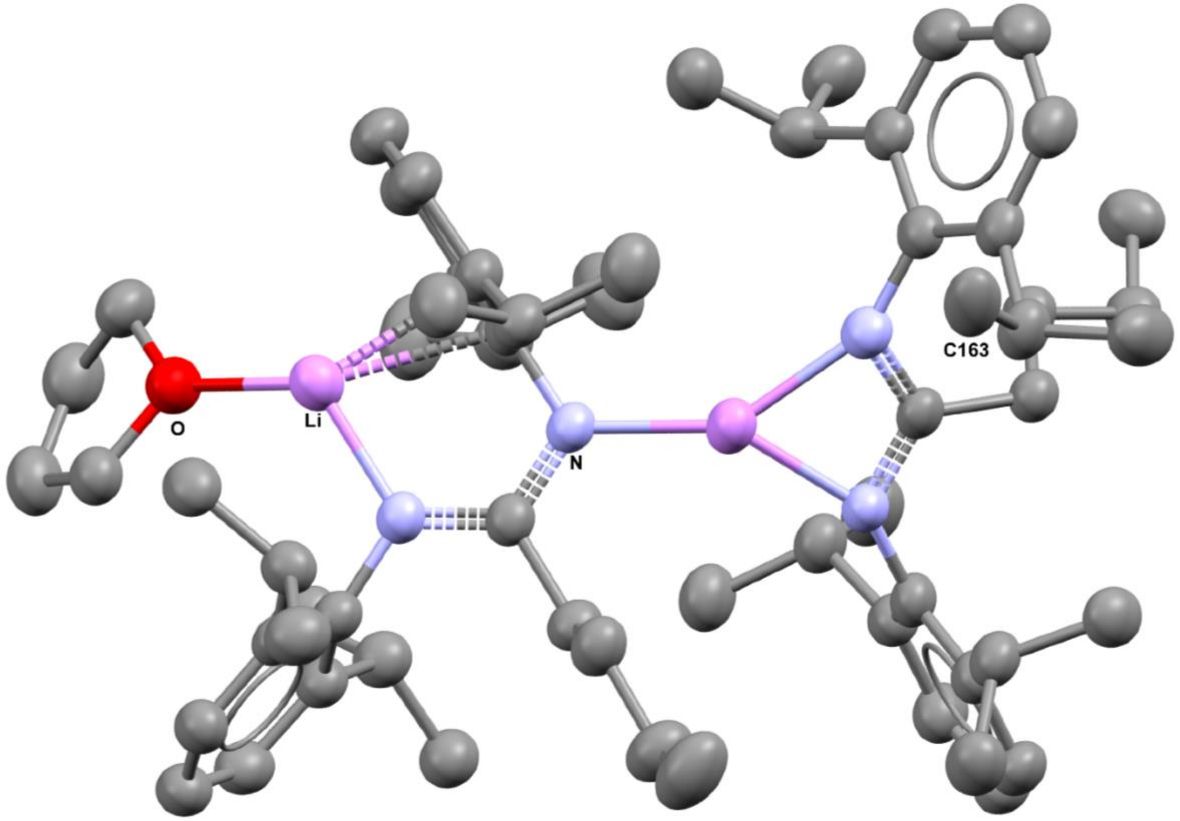

Figure S31. Solid state structure of **[(Li(NDipp)$_2$C$_5$H$_9$)$_2$THF]**, thermal ellipsoids displayed at a 50% probability. Hydrogen atoms are omitted, as well as disordered moiety (2 positions of C163) represented as single occupancies for clarity. Purple, blue, red and gray ellipsoids represent lithium, nitrogen, oxygen, and carbon respectively

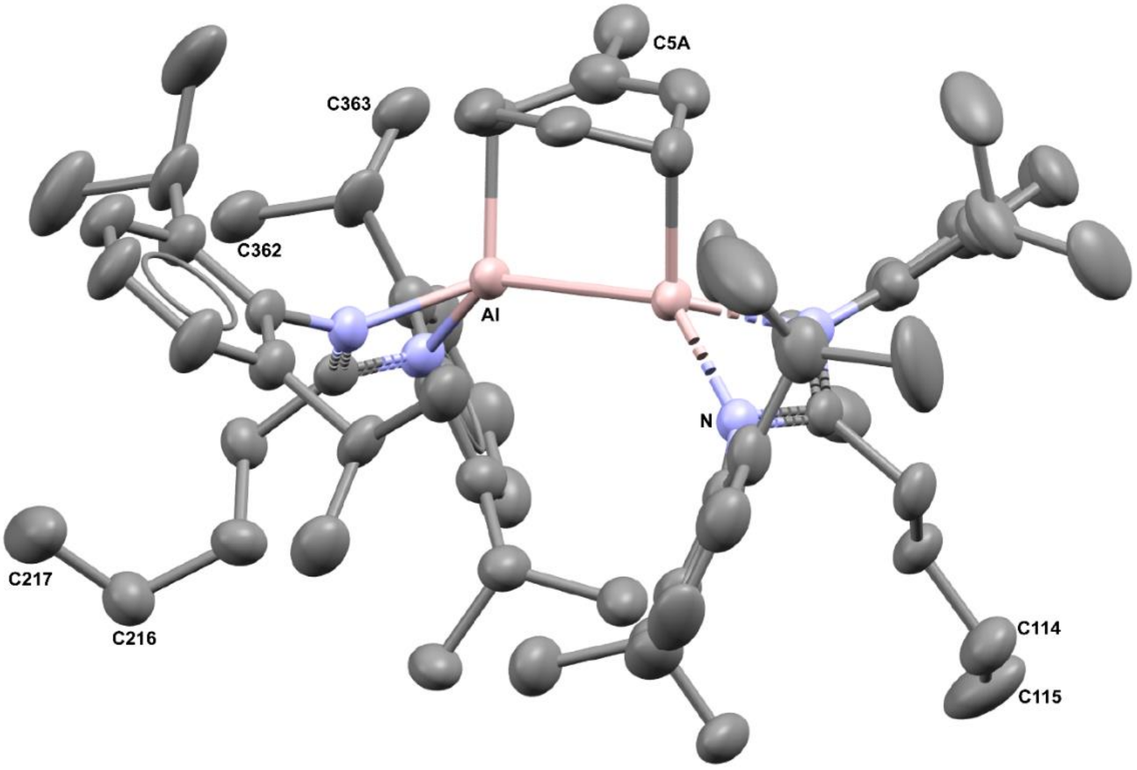

Figure S32. Solid state structure of **$[(Al(NDipp)_2C_5H_9)_2C_7H_8]\cdot C_7H_8$**, thermal ellipsoids displayed at a 50% probability. Hydrogen atoms and outer sphere solvents (C_7H_8 modeled using a solvent mask) are omitted and disordered moieties (4 positions of C5A, and 2 positions of C114, C115; C216, C217; and C362, C363) represented as single occupancies for clarity. Pink, blue and gray ellipsoids represent aluminum, nitrogen, and carbon respectively

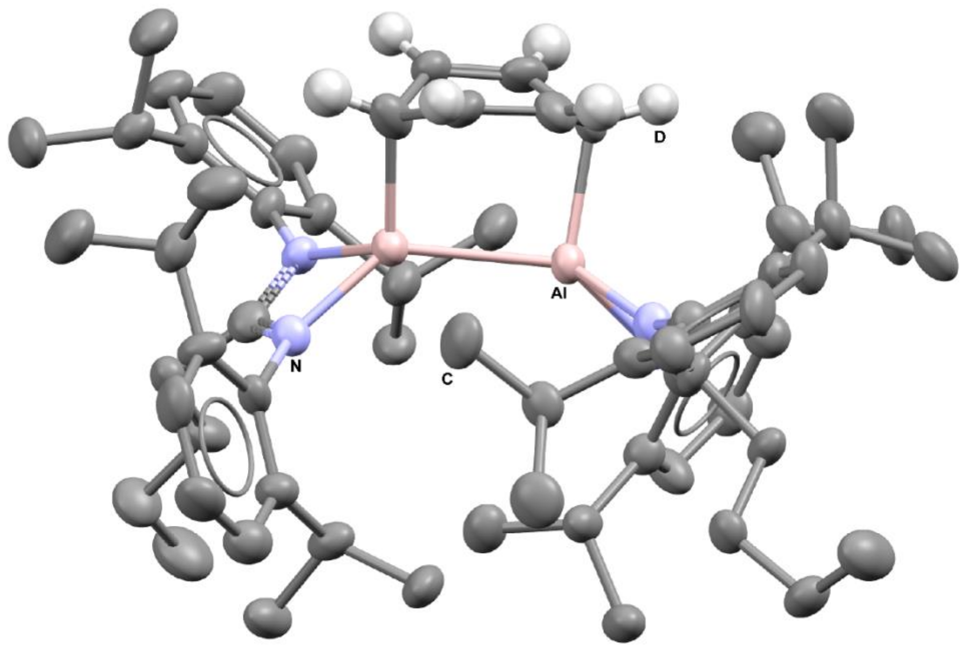

Figure S33. Solid state structure of **$[(Al(NDipp)_2C_5H_9)_2C_6D_6]\cdot C_6H_{14}$**, thermal ellipsoids displayed at a 50% probability. Hydrogen atoms and outer-sphere solvent molecules (C_6H_{14} modeled using a solvent mask) are omitted for clarity. Pink, blue, gray, and white ellipsoids represent aluminum, nitrogen, carbon, and deuterium respectively

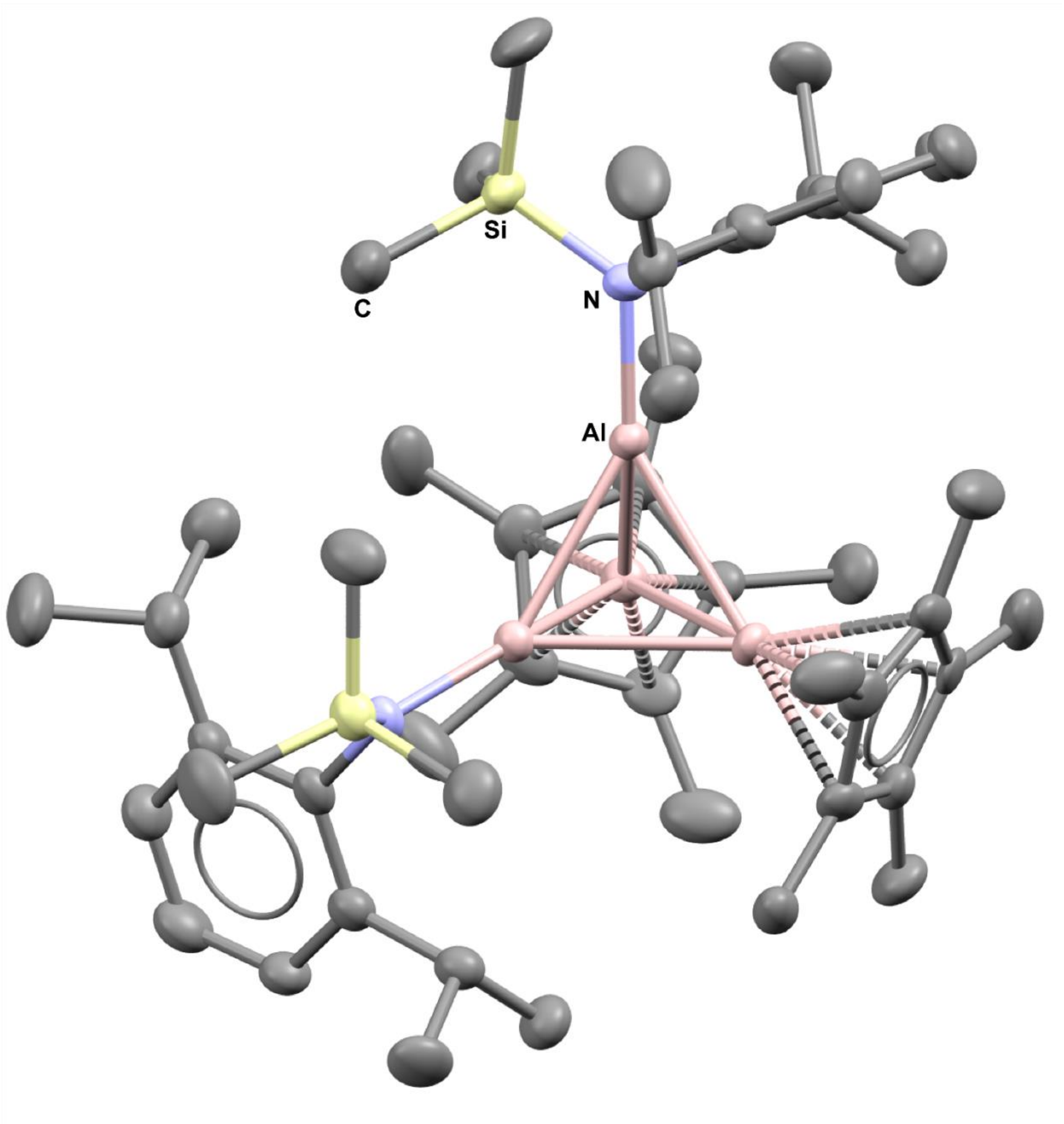

Figure S34. Solid state structure of **[AlNTMSDippAlCp*]$_2$·C$_6$H$_{14}$**, Thermal ellipsoids are displayed at 50% probability. Hydrogen atoms and outer-sphere solvent molecules (C_6H_{14} was explicitly modeled) are omitted for clarity. Pink, blue, yellow, and gray ellipsoids represent aluminum, nitrogen, silicon and carbon respectively

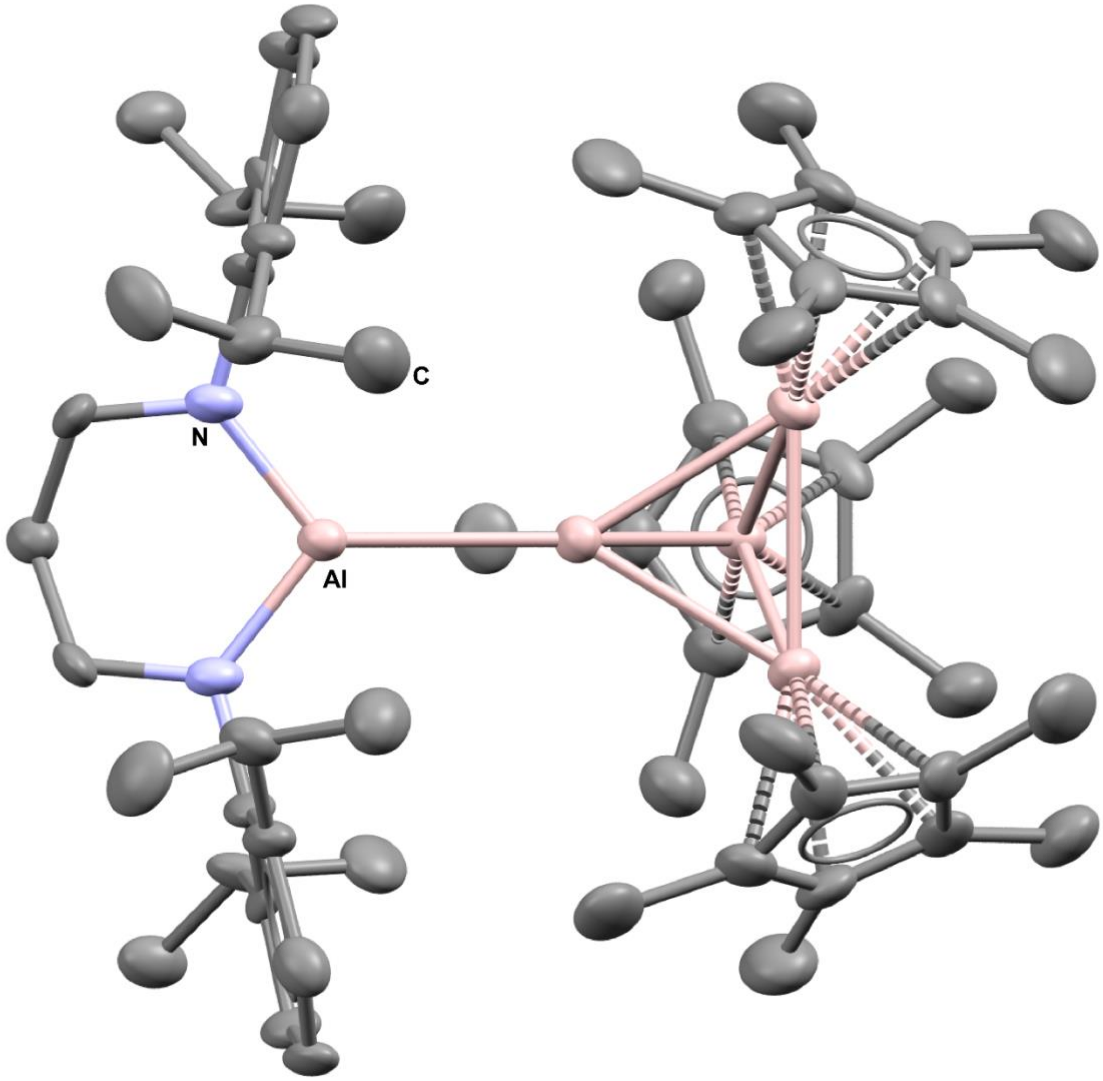

Figure S35. Solid state structure of **[Al(N_2Dipp$_2$$C_3H_6$)($Al_4$Cp*$_3$)]·0.5C6H6**, thermal ellipsoids displayed at a 50% probability. Hydrogen atoms and outer sphere solvent molecules (0.5 C_6H_6 was explicitly modeled) are omitted for clarity. Blue, gray, and pink ellipsoids represent nitrogen, carbon, and aluminum respectively

6. FTIR data

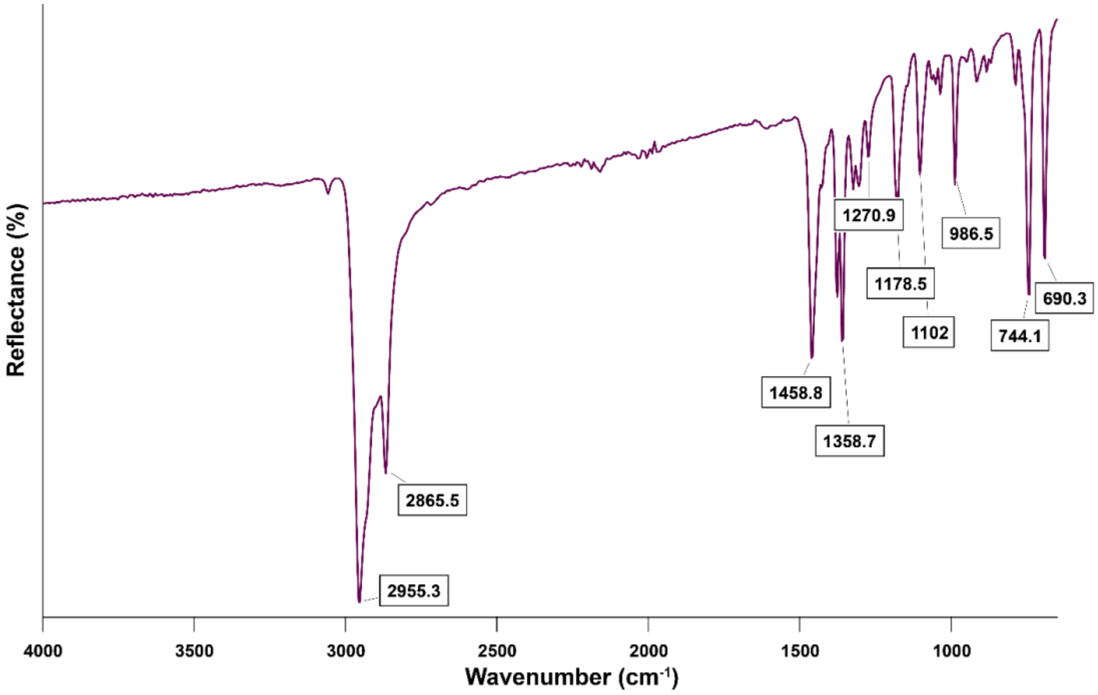

Figure S36. FTIR-ATR of **NaCp^{iPr4}**

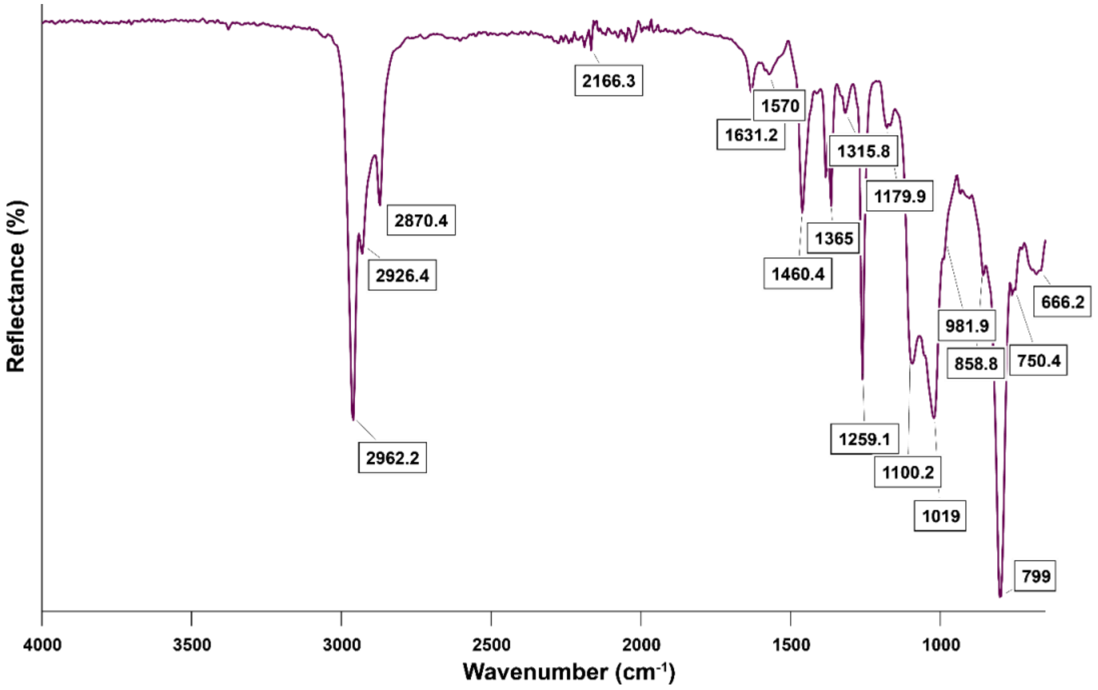

Figure S37. FTIR-ATR of **AlCp^{iPr4}**

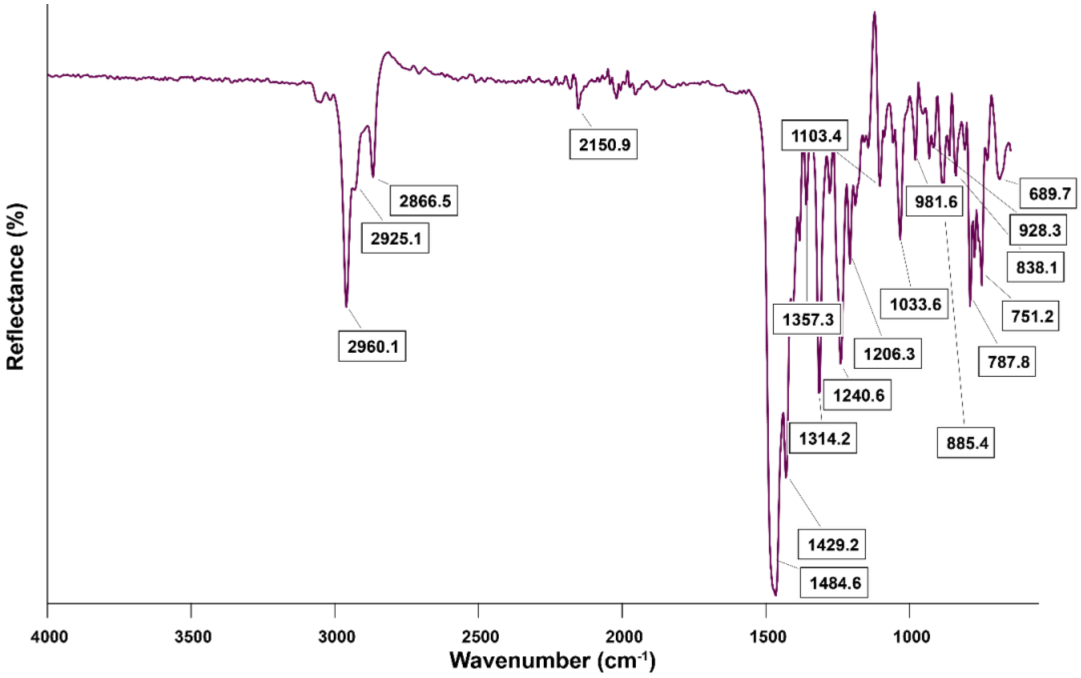

Figure S38. FTIR-ATR of **[(Li(NDipp)$_2$C$_5$H$_9$)$_2$THF]**

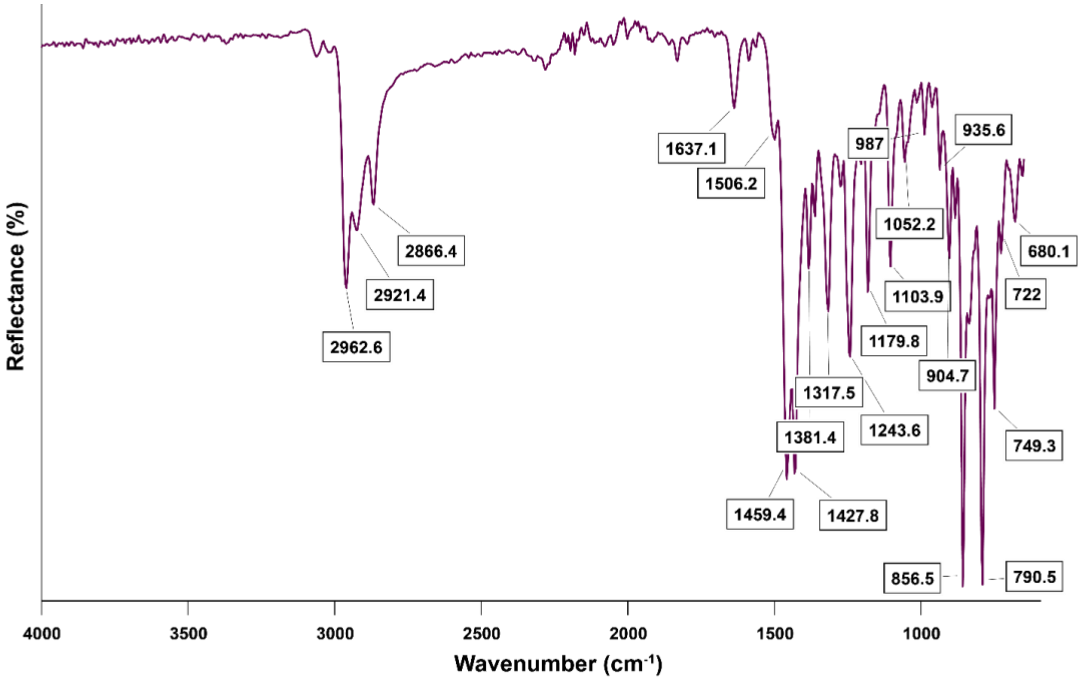

Figure S39. FTIR-ATR of **[(Al(NDipp)$_2$C$_5$H$_9$)$_2$C$_7$H$_8$]·C$_7$H$_8$**

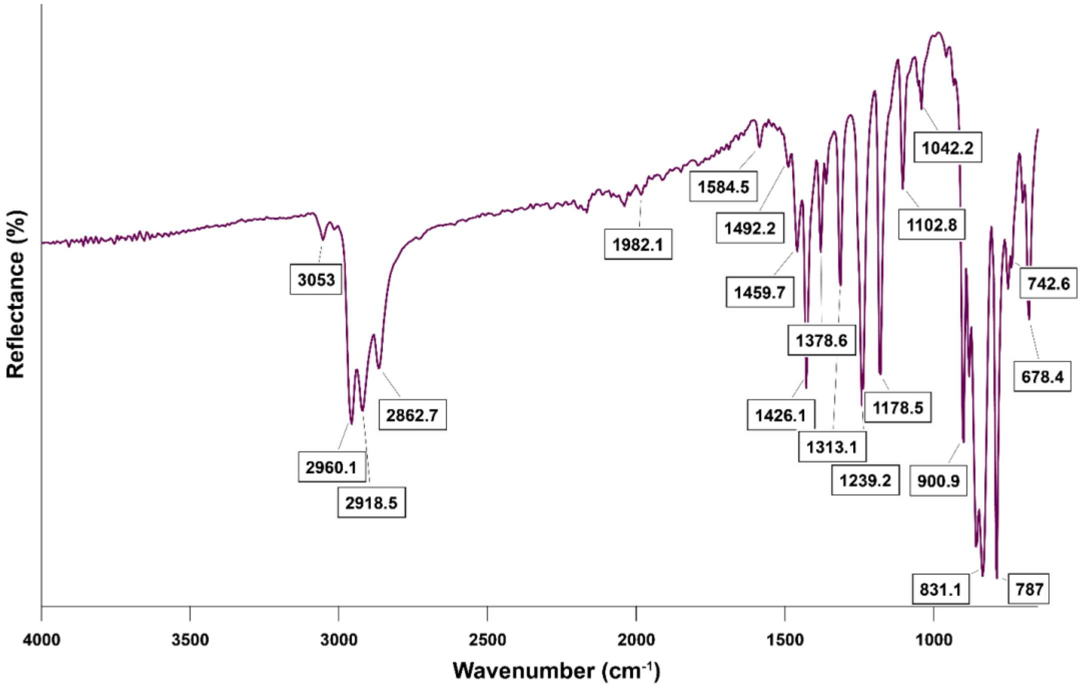

Figure S40. FTIR-ATR of **[AlNTMSDippAlCp*]$_2$·C_6H_{14}**

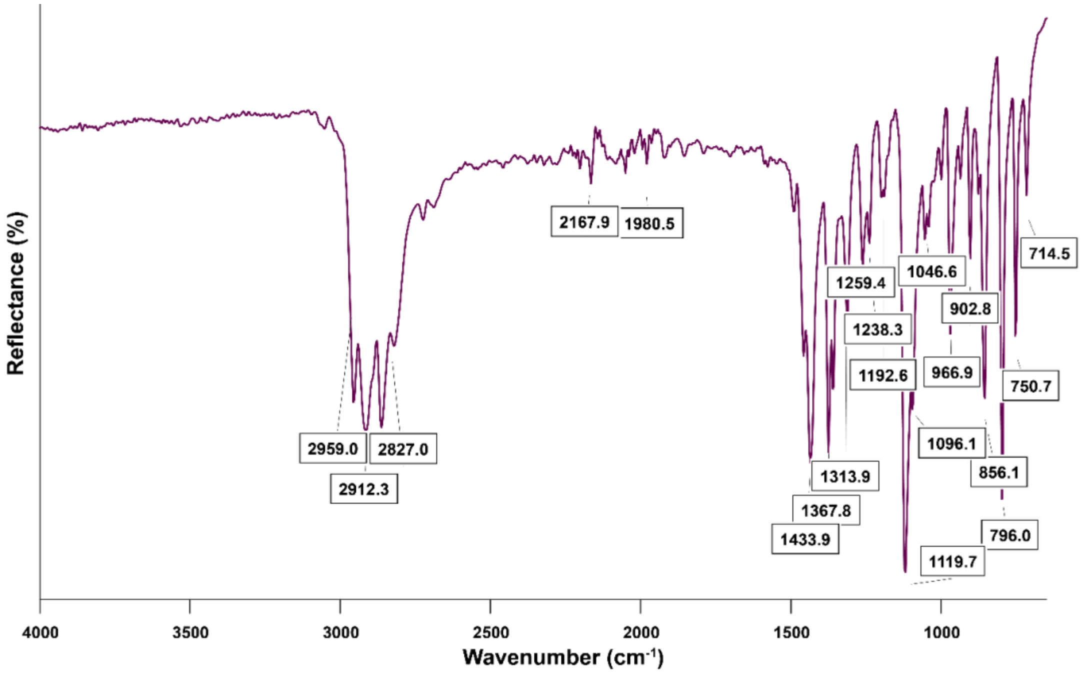

Figure S41. FTIR-ATR of **[Al(N_2Dipp$_2$$C_3H_6$)($Al_4$Cp*$_3$)]·0.5$C_6H_6$**

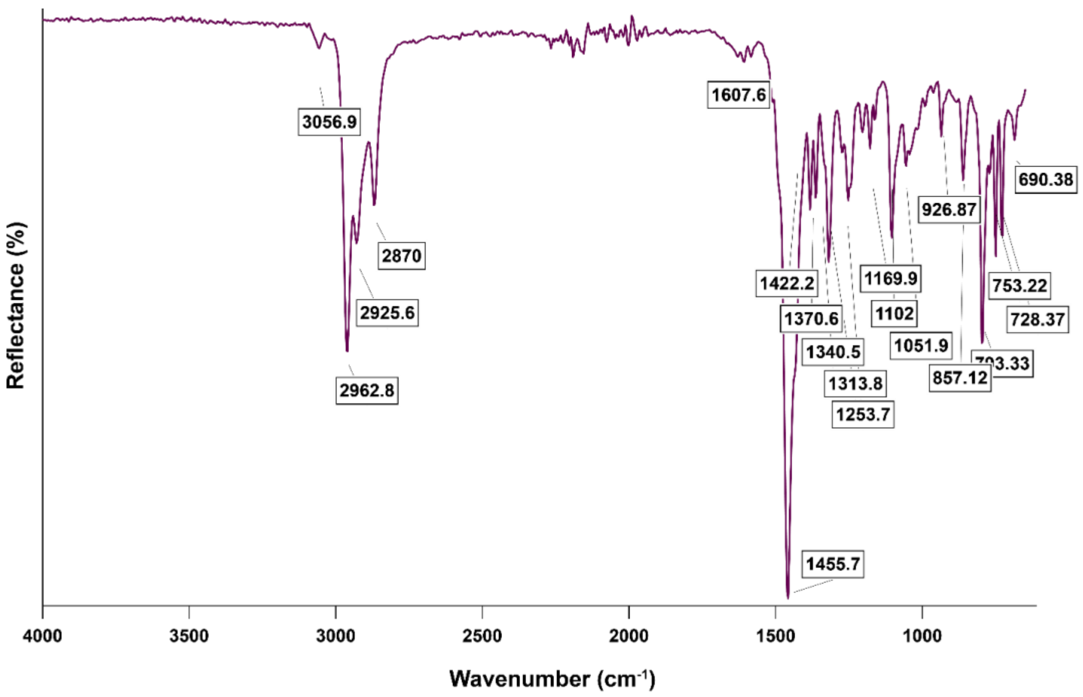

Figure S42. FTIR-ATR of **$[(Al(NDipp)_2C_5H_9)_2C_6D_6]\cdot C_6H_{14}$**

7. UV-Vis

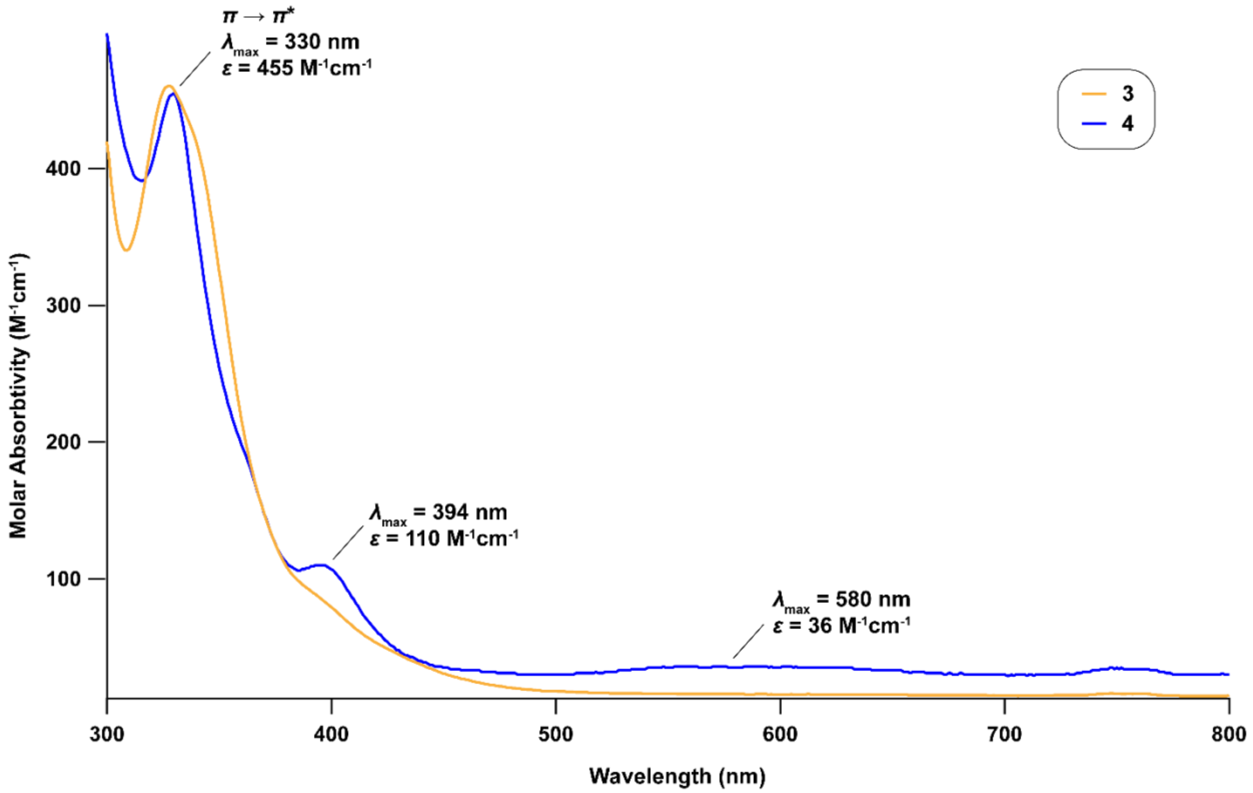

Figure S43. UV-Vis Spectra of **[AlNTMSDippAlCp*]$_2$·C_6H_{14}** (**3**) and **[Al(N_2Dipp$_2$$C_3H_6$)($Al_4$Cp*$_3$)]·0.5$C_6H_6$** (**4**).

8. Molecular orbital (MO) isosurface

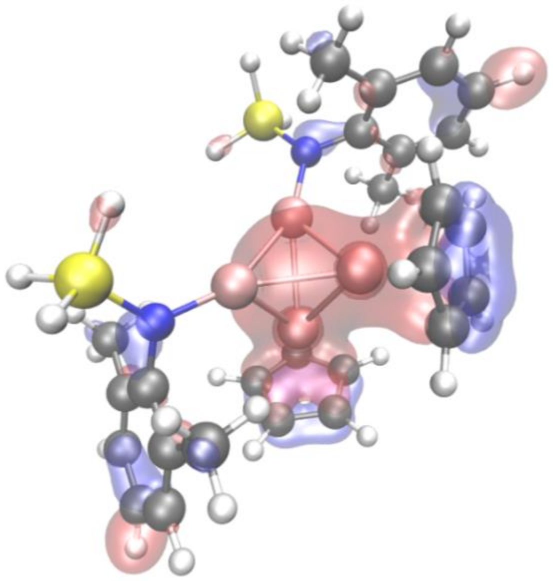

Figure S44. Model **3** isosurface of MO−93, HOMO-49 (isovalue = -0.03, 0.03). Yellow, pink, blue, gray and white atoms represent silicon, aluminum, nitrogen, carbon and hydrogen respectively.

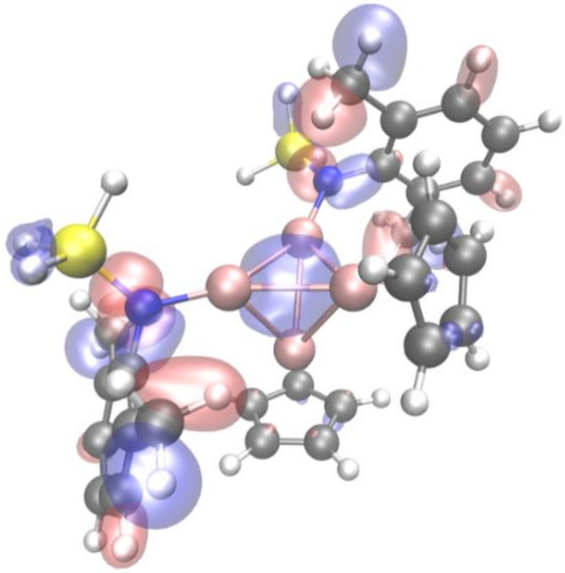

Figure S45. Model **3** isosurface of MO−111 HOMO-31 (isovalue = -0.03, 0.03). Yellow, pink, blue, gray and white atoms represent silicon, aluminum, nitrogen, carbon and hydrogen respectively.

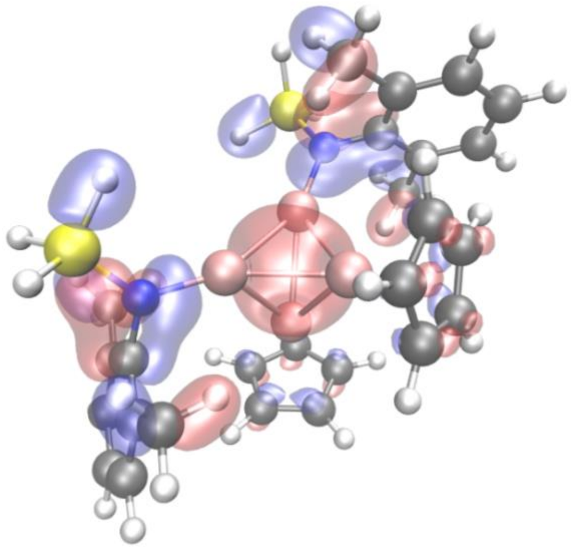

Figure S46. Model **3** isosurface of MO−119 HOMO-23 (isovalue = -0.03, 0.03). Yellow, pink, blue, gray and white atoms represent silicon, aluminum, nitrogen, carbon and hydrogen respectively.

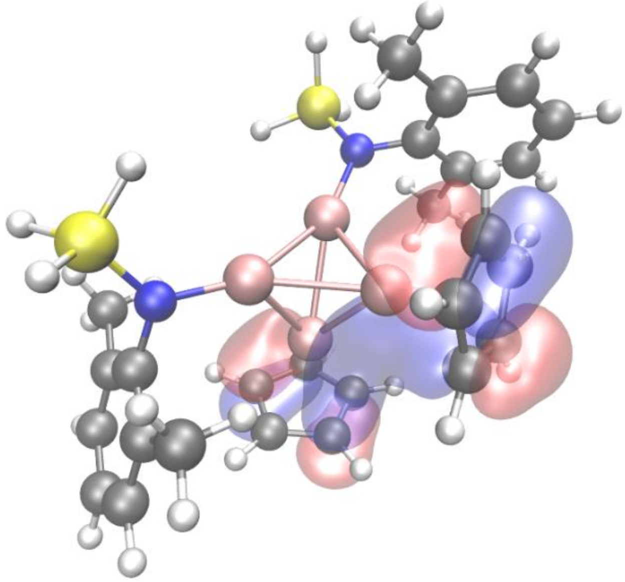

Figure S47. Model **3** isosurface of MO−130 HOMO-12 (isovalue = -0.03, 0.03). Yellow, pink, blue, gray and white atoms represent silicon, aluminum, nitrogen, carbon and hydrogen respectively.

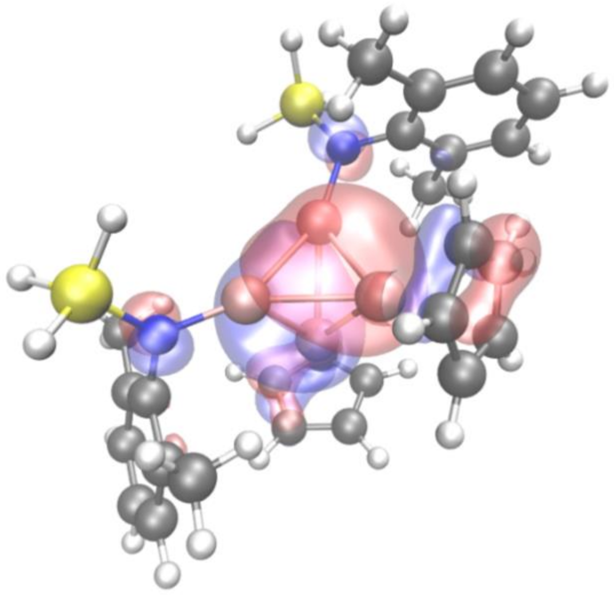

Figure S48. Model **3** isosurface of MO−140 HOMO-2 (isovalue = -0.03, 0.03). Yellow, pink, blue, gray and white atoms represent silicon, aluminum, nitrogen, carbon and hydrogen respectively.

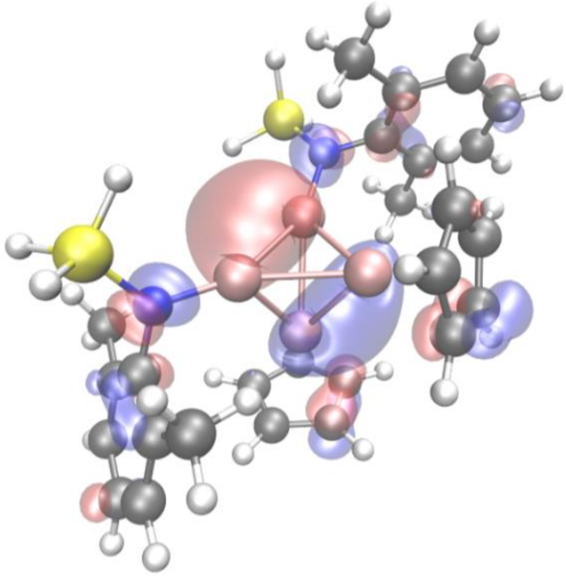

Figure S49. Model **3** isosurface of MO−141 HOMO-1 (isovalue = -0.03, 0.03). Yellow, pink, blue, gray and white atoms represent silicon, aluminum, nitrogen, carbon and hydrogen respectively.

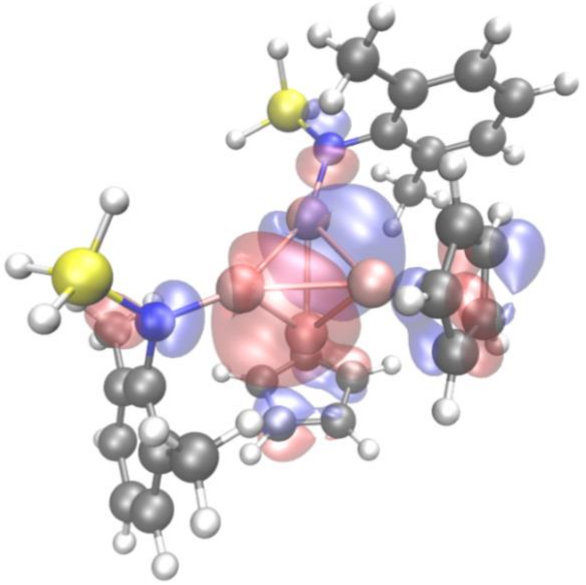

Figure S50. Model **3** isosurface of MO−142 HOMO (isovalue = -0.03, 0.03). Yellow, pink, blue, gray and white atoms represent silicon, aluminum, nitrogen, carbon and hydrogen respectively.

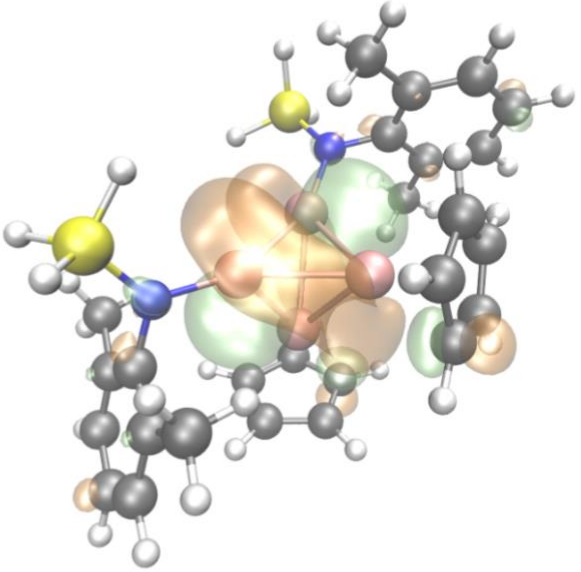

Figure S51. Model **3** isosurface of MO−143 LUMO (isovalue = -0.03, 0.03). Yellow, pink, blue, gray and white atoms represent silicon, aluminum, nitrogen, carbon and hydrogen respectively.

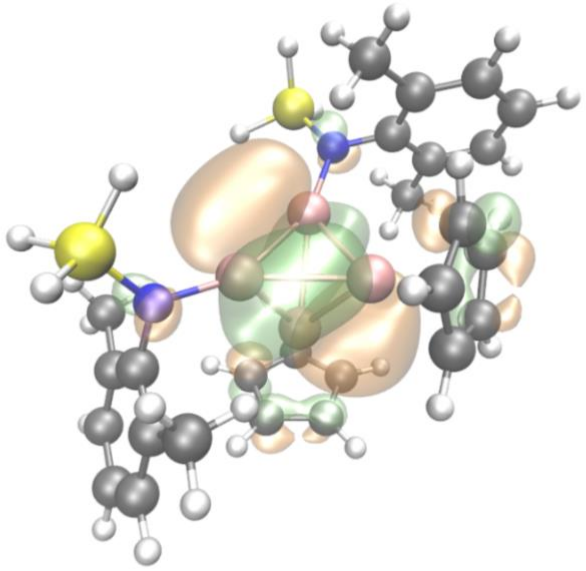

Figure S52. Model **3** isosurface of MO−144 LUMO+1 (isovalue = -0.03, 0.03). Yellow, pink, blue, gray and white atoms represent silicon, aluminum, nitrogen, carbon and hydrogen respectively.

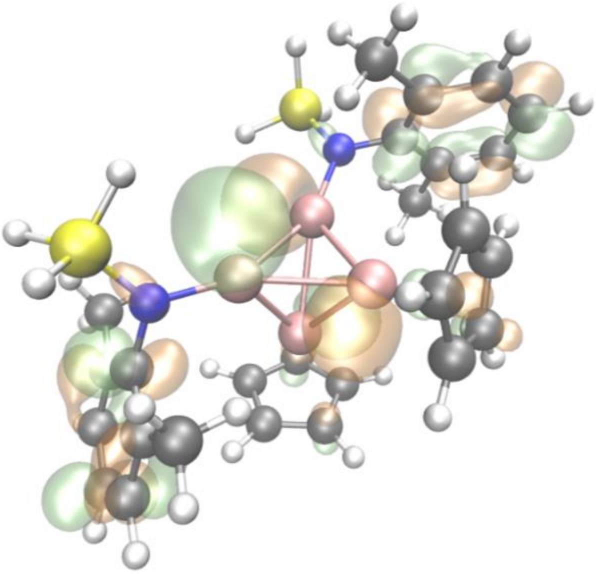

Figure S53. Model **3** isosurface of MO−145 LUMO+2 (isovalue = -0.03, 0.03). Yellow, pink, blue, gray and white atoms represent silicon, aluminum, nitrogen, carbon and hydrogen respectively.

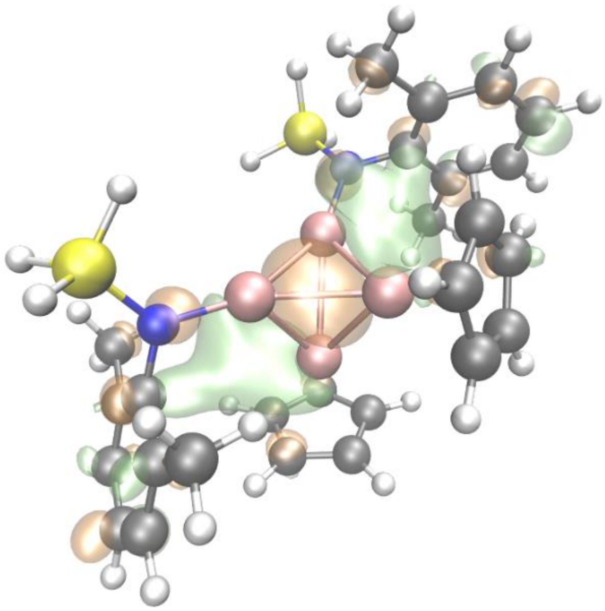

Figure S54. Model **3** isosurface of MO−146 LUMO+3 (isovalue = -0.03, 0.03). Yellow, pink, blue, gray and white atoms represent silicon, aluminum, nitrogen, carbon and hydrogen respectively.

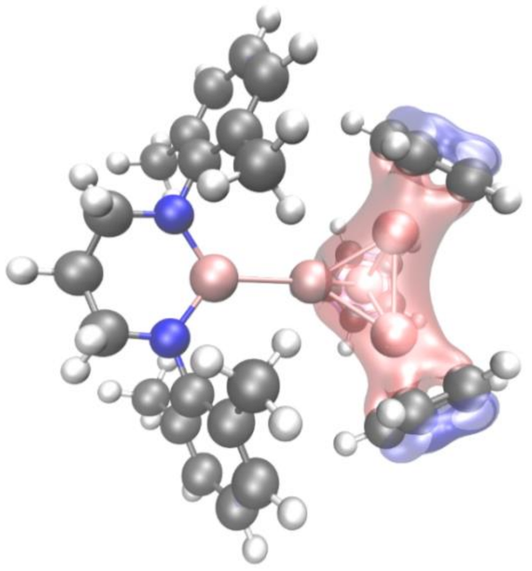

Figure S55. Model **4** isosurface of MO−104, HOMO-57 (isovalue = -0.03, 0.03). Pink, blue, gray and white atoms represent aluminum, nitrogen, carbon and hydrogen respectively.

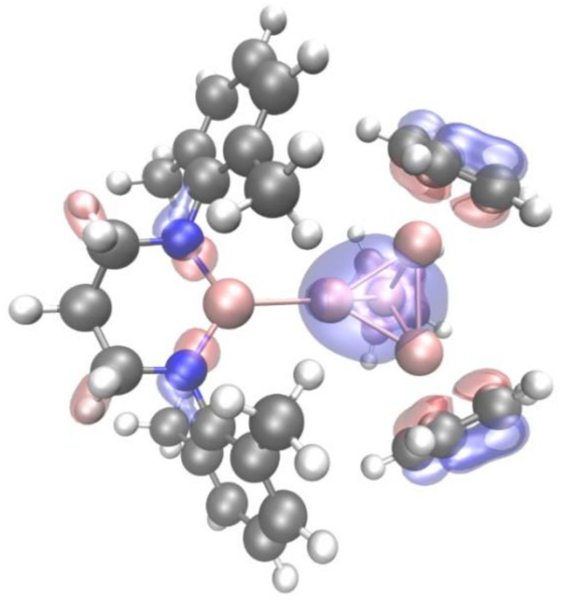

Figure S56. Model **4** isosurface of MO−134, HOMO-27 (isovalue = -0.03, 0.03). Pink, blue, gray and white atoms represent aluminum, nitrogen, carbon and hydrogen respectively.

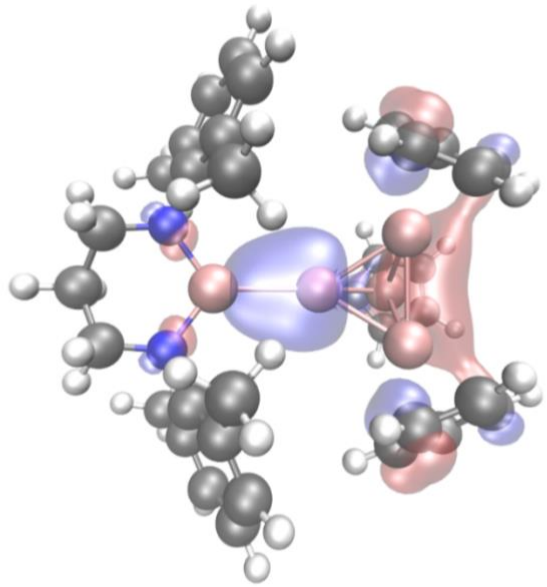

Figure S57. Model **4** isosurface of MO−146, HOMO-15 (isovalue = -0.03, 0.03). Pink, blue, gray and white atoms represent aluminum, nitrogen, carbon and hydrogen respectively.

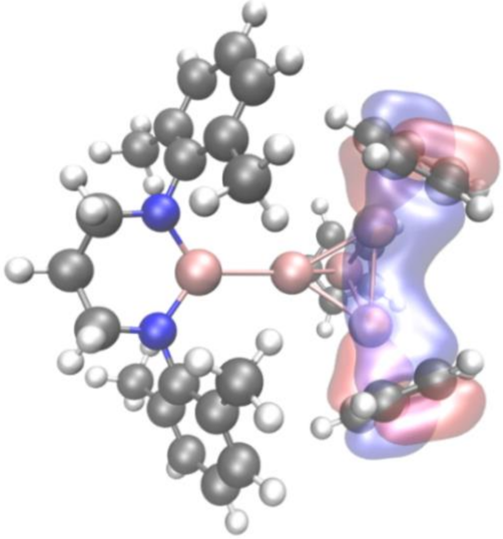

Figure S58. Model **4** isosurface of MO−147, HOMO-14 (isovalue = -0.03, 0.03). Pink, blue, gray and white atoms represent aluminum, nitrogen, carbon and hydrogen respectively.

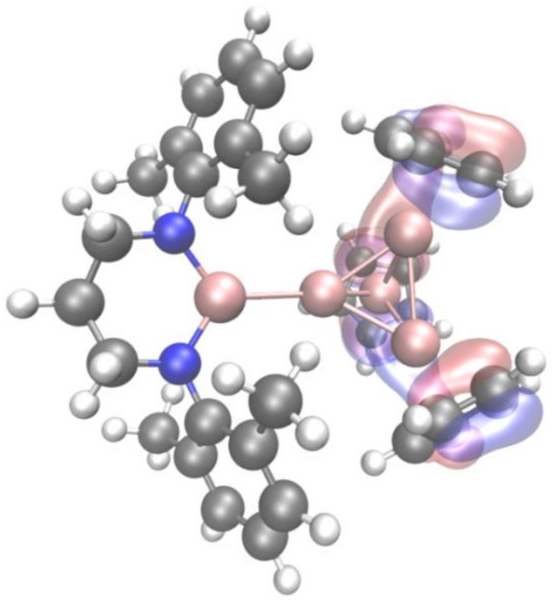

Figure S59. Model **4** isosurface of MO−148, HOMO-13 (isovalue = -0.03, 0.03). Pink, blue, gray and white atoms represent aluminum, nitrogen, carbon and hydrogen respectively.

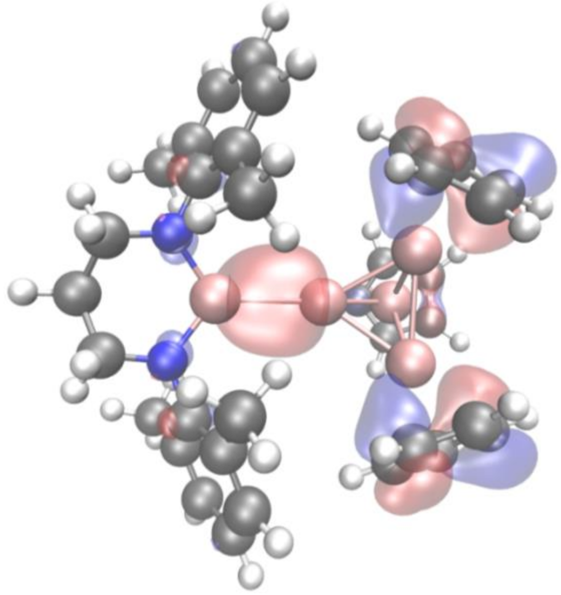

Figure S60. Model **4** isosurface of MO−151, HOMO-10 (isovalue = -0.03, 0.03). Pink, blue, gray and white atoms represent aluminum, nitrogen, carbon and hydrogen respectively.

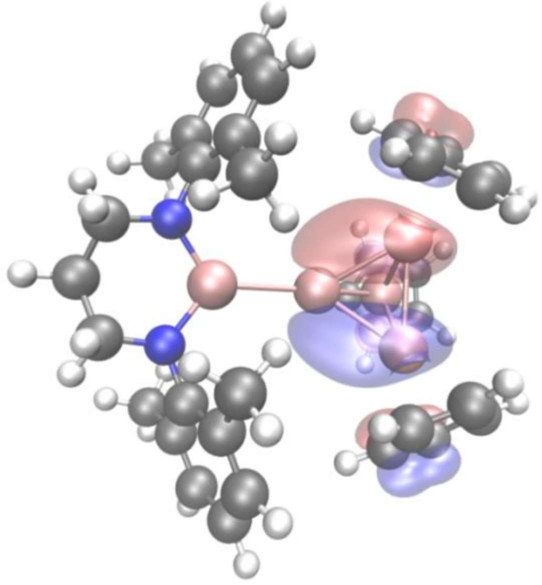

Figure S61. Model **4** isosurface of MO−159, HOMO-2 (isovalue = -0.03, 0.03). Pink, blue, gray and white atoms represent aluminum, nitrogen, carbon and hydrogen respectively.

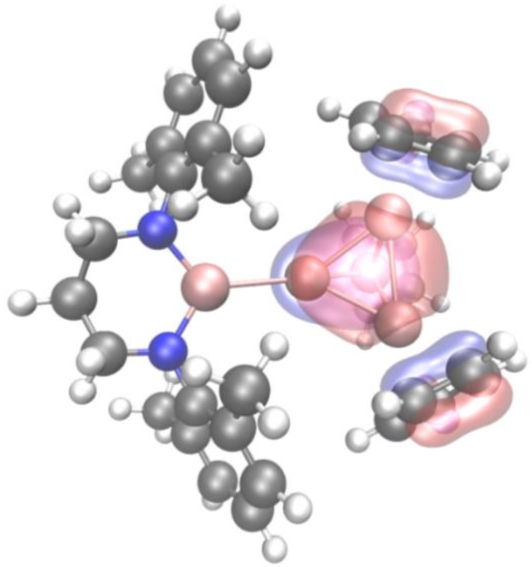

Figure S62. Model **4** isosurface of MO−160, HOMO-1 (isovalue = -0.03, 0.03). Pink, blue, gray and white atoms represent aluminum, nitrogen, carbon and hydrogen respectively.

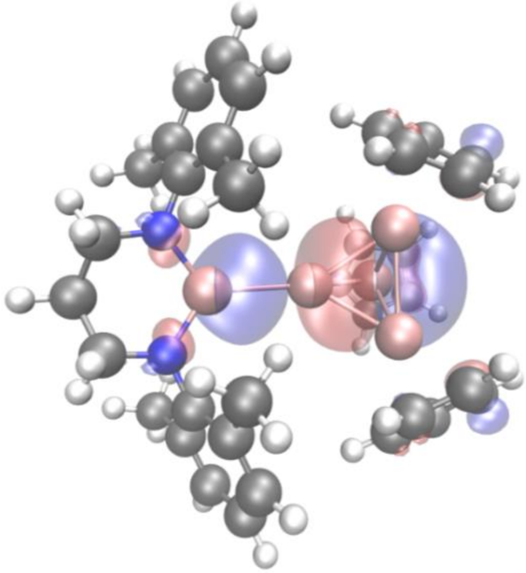

Figure S63. Model **4** isosurface of MO−161, HOMO (isovalue = -0.03, 0.03). Pink, blue, gray and white atoms represent aluminum, nitrogen, carbon and hydrogen respectively.

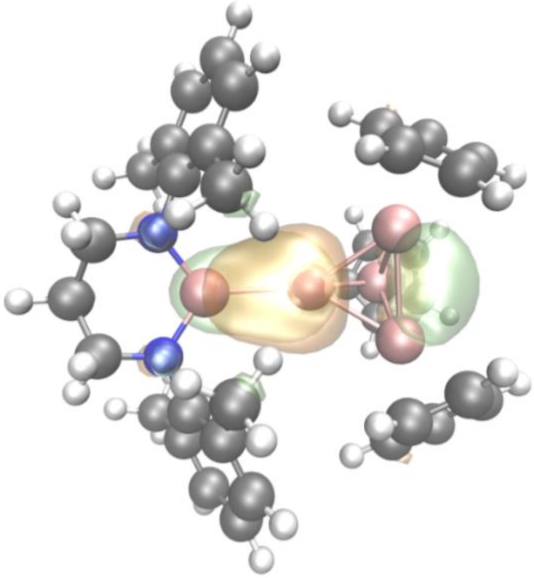

Figure S64. Model **4** isosurface of MO−162, LUMO (isovalue = -0.03, 0.03). Pink, blue, gray and white atoms represent aluminum, nitrogen, carbon and hydrogen respectively.

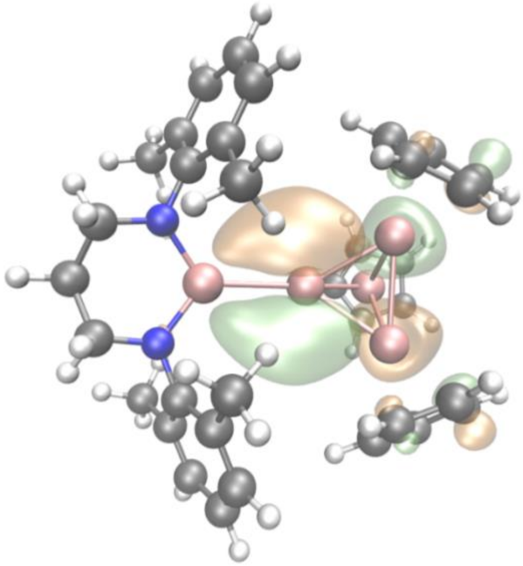

Figure S65. Model **4** isosurface of MO−163, LUMO+1 (isovalue = -0.03, 0.03). Pink, blue, gray and white atoms represent aluminum, nitrogen, carbon and hydrogen respectively.

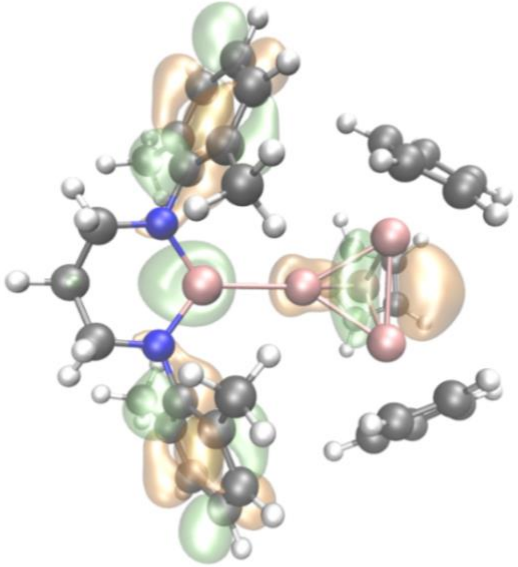

Figure S66. Model **4** isosurface of MO−164, LUMO+2 (isovalue = -0.03, 0.03). Pink, blue, gray and white atoms represent aluminum, nitrogen, carbon and hydrogen respectively.

9. ELF plots

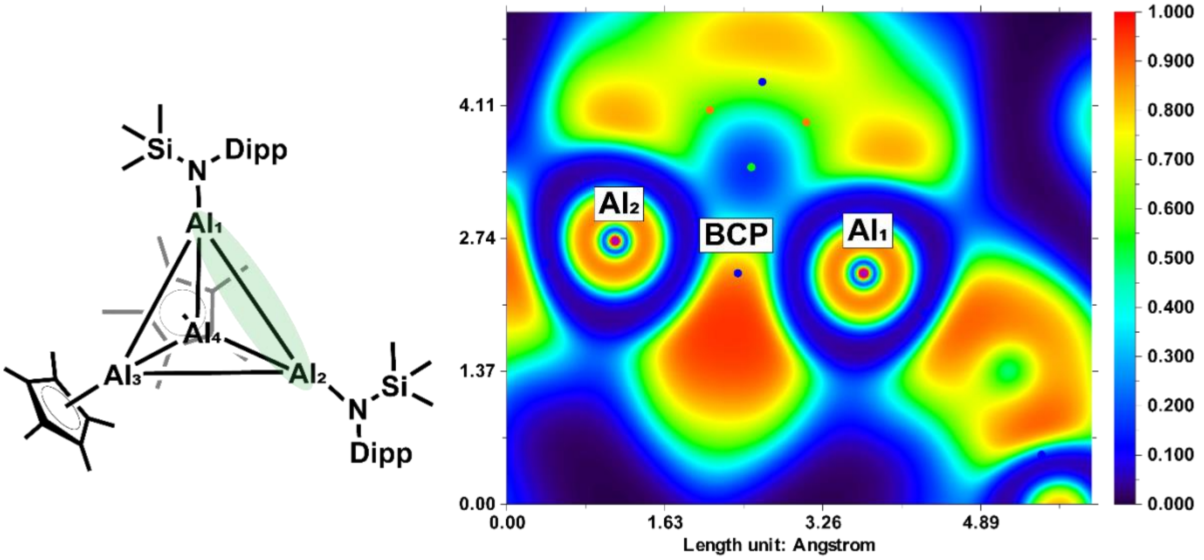

Figure S67. Electron localization function (ELF) map of model **3** plotted in the plane defined by Al1 and Al2 and passing through the Al1–Al2 bond critical point (BCP). At this BCP, the dominant contributions to the electron density arise from MO−141 (51.24%), MO−119 (10.45%), MO−93 (7.58%), and MO−111 (6.28%).

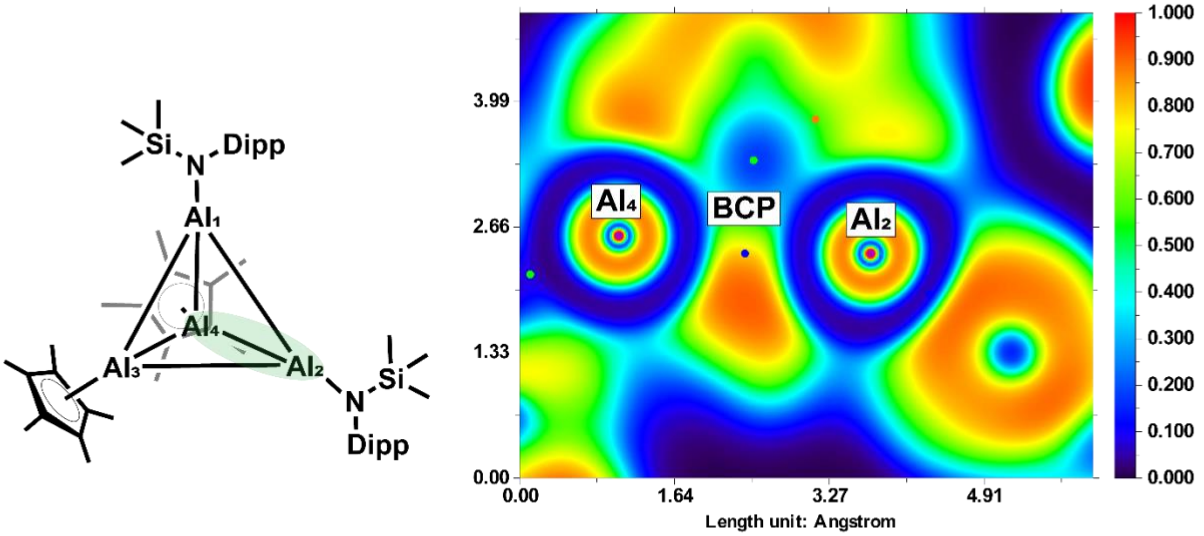

Figure S68. Electron localization function (ELF) map of model **3** plotted in the plane defined by Al2 and Al4 and passing through the Al2–Al4 bond critical point (BCP). At this BCP, the dominant contributions to the electron density arise from MO−142 (26.09%), MO−140 (19.65%), MO−119 (12.75%), MO−93 (10.66%) and MO−111 (5.29%).

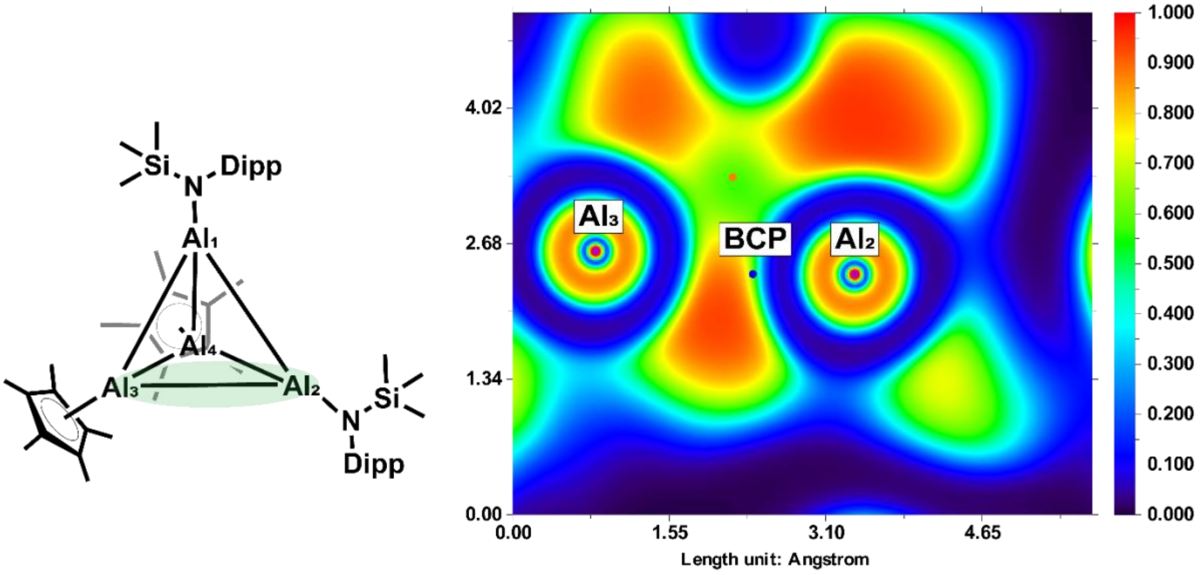

Figure S69. Electron localization function (ELF) map of model **3** plotted in the plane defined by Al2 and Al3 and passing through the Al2–Al3 bond critical point (BCP). At this BCP, the dominant contributions to the electron density arise from MO−142 (30.41%), MO−140 (20.95%), MO−119 (9.80%), MO−93 (9.63%) and MO−111 (5.34%).

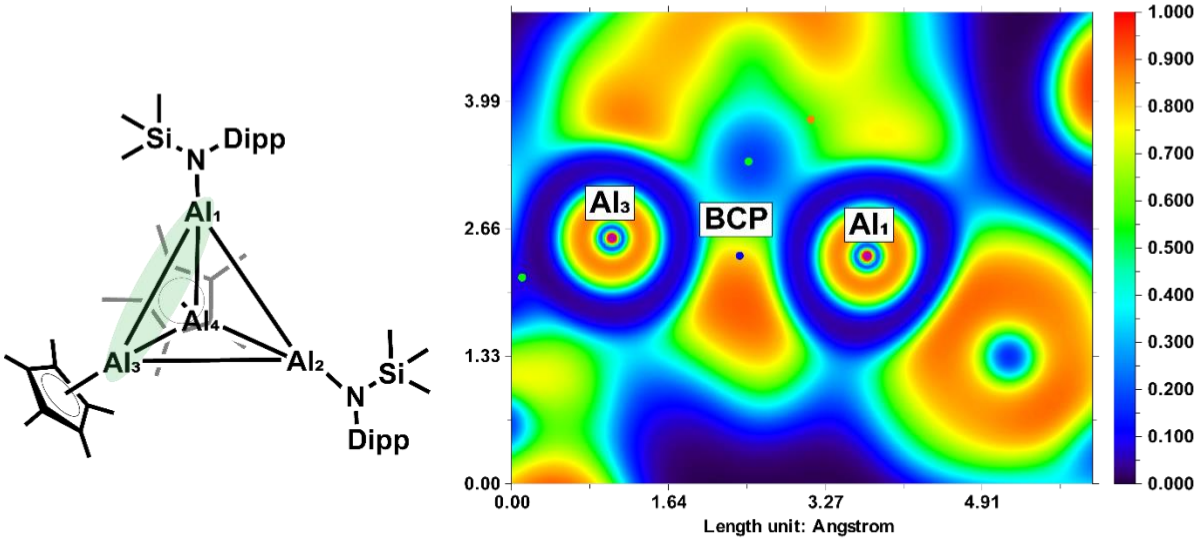

Figure S70. Electron localization function (ELF) map of model **3** plotted in the plane defined by Al1 and Al3 and passing through the Al1–Al3 bond critical point (BCP). At this BCP, the dominant contributions to the electron density arise from MO−142 (26.07%), MO−140 (19.64%), MO−119 (12.63%), MO−93 (10.69%) and MO−111 (5.31%).

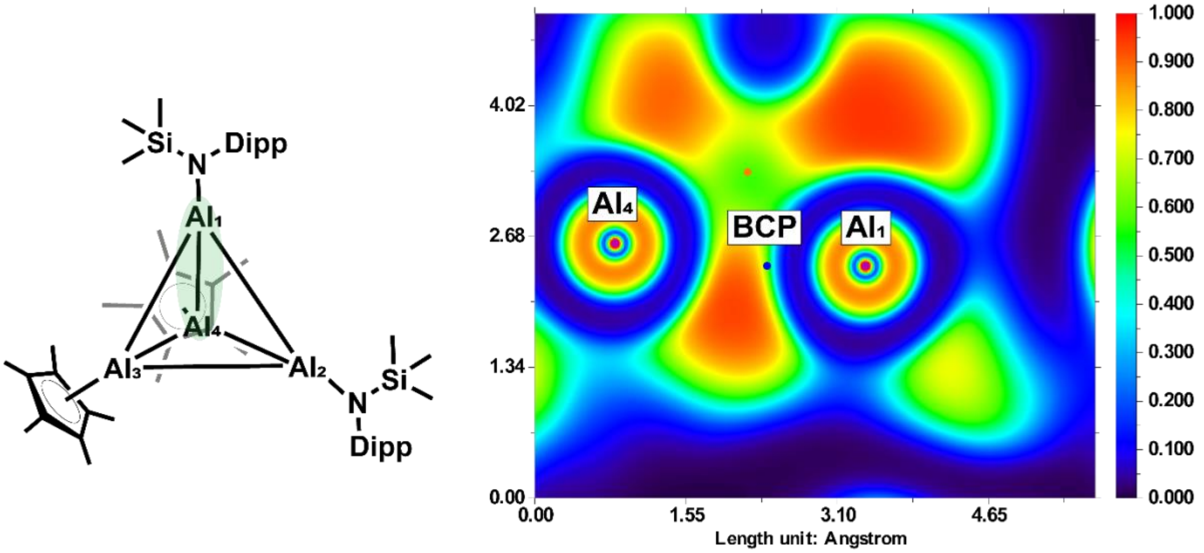

Figure S71. Electron localization function (ELF) map of model **3** plotted in the plane defined by Al1 and Al4 and passing through the Al1–Al4 bond critical point (BCP). At this BCP, the dominant contributions to the electron density arise from MO−142 (30.46%), MO−140 (20.90%), MO−119 (9.83%), MO−93 (9.64%) and MO−111 (5.38%).

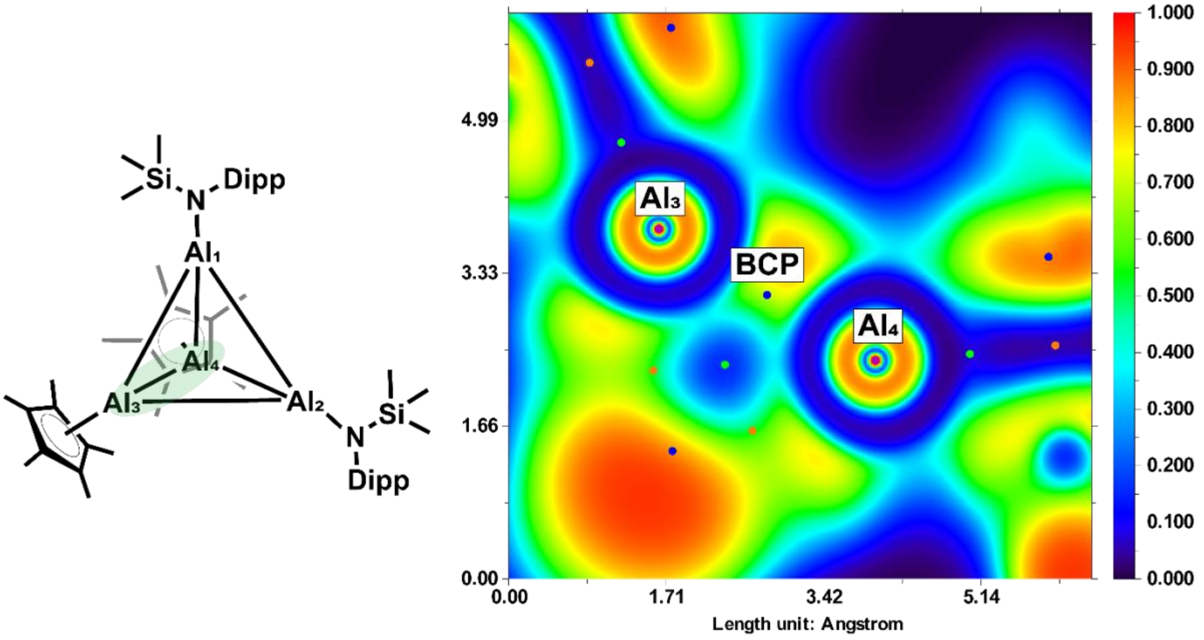

Figure S72. Electron localization function (ELF) map of model **3** plotted in the plane defined by Al3 and Al4 and passing through the Al3–Al4 bond critical point (BCP). At this BCP, the dominant contributions to the electron density arise from MO−141 (36.49%), MO−119 (15.37%), MO−93 (14.84%), MO−130 (7.81%) and MO−111 (5.01%).

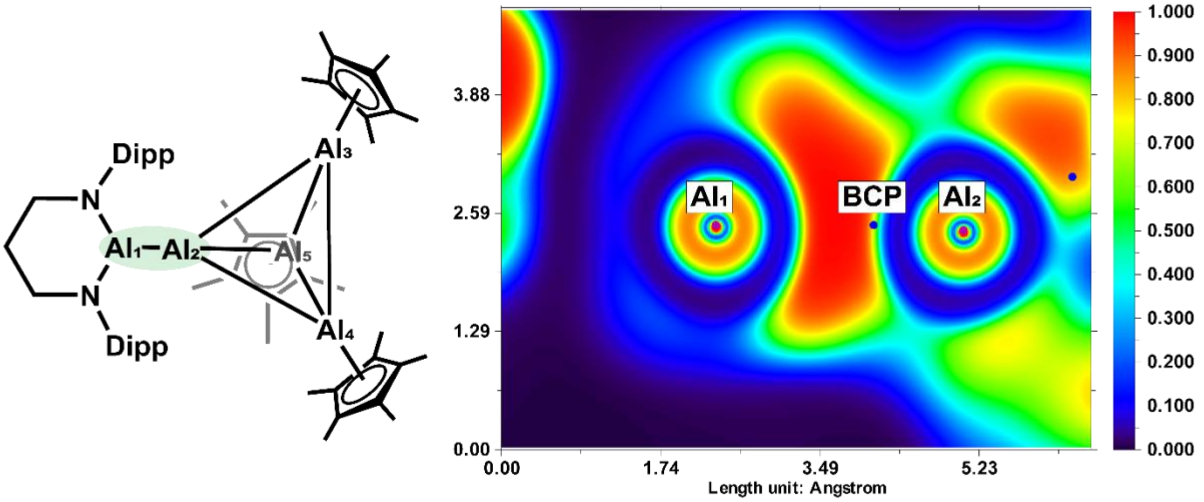

Figure S73. Electron localization function (ELF) map of model **4** plotted in the plane defined by Al1 and Al2 and passing through the Al1–Al2 bond critical point (BCP). At this BCP, the dominant contributions to the electron density arise from MO−151 (33.14%), MO−146 (32.99%), and MO−161 (14.66%).

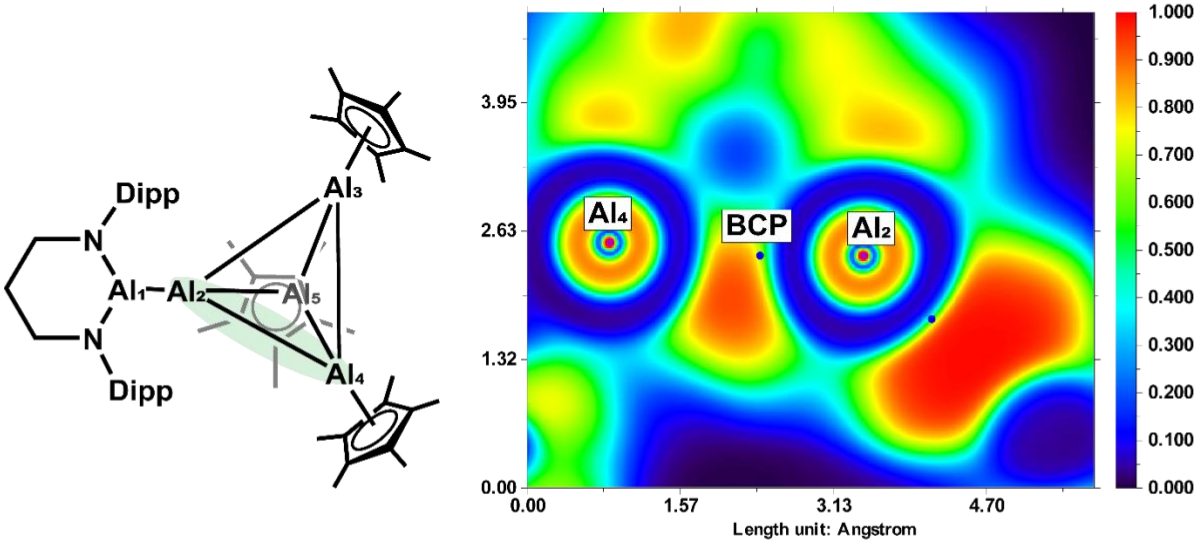

Figure S74. Electron localization function (ELF) map of model **4** plotted in the plane defined by Al2 and Al4 and passing through the Al2–Al4 bond critical point (BCP). At this BCP, the dominant contributions to the electron density arise from MO−161 (25.87%), MO−134 (24.34%), MO−159 (20.37%), MO−104 (10.99%), and MO−160 (6.00%).

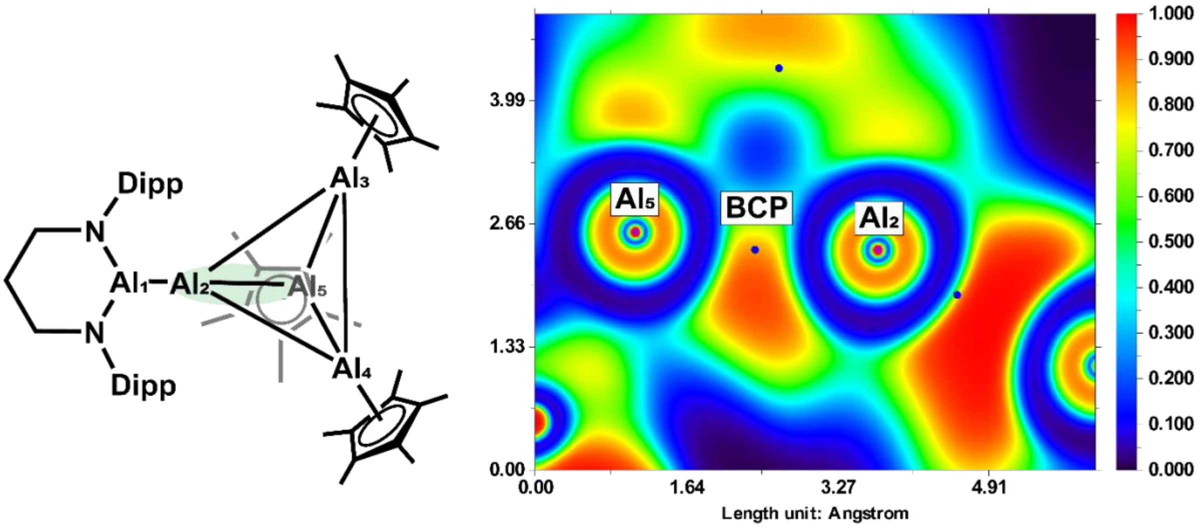

Figure S75. Electron localization function (ELF) map of model **4** plotted in the plane defined by Al2 and Al5 and passing through the Al2–Al5 bond critical point (BCP). At this BCP, the dominant contributions to the electron density arise from MO−160 (36.14%), MO−134 (22.44%), MO−161 (18.74%), and MO−104 (11.07%).

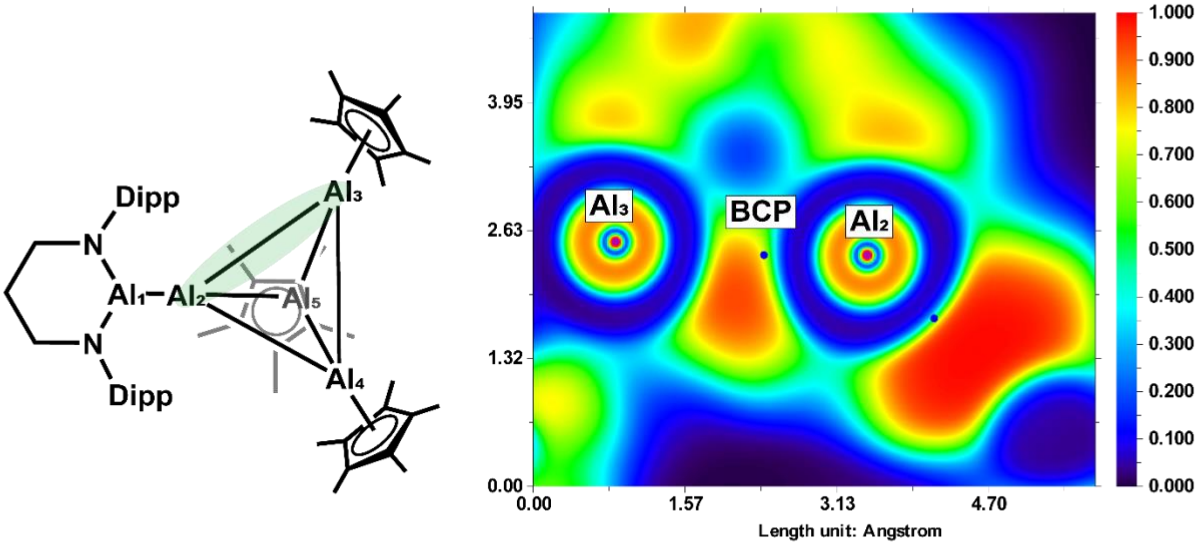

Figure S76. Electron localization function (ELF) map of model **4** plotted in the plane defined by Al2 and Al3 and passing through the Al2–Al3 bond critical point (BCP). At this BCP, the dominant contributions to the electron density arise from MO−161 (25.72%), MO−134 (24.39%), MO−159 (20.88%), MO−104 (11.02%), and MO−160 (5.60%).

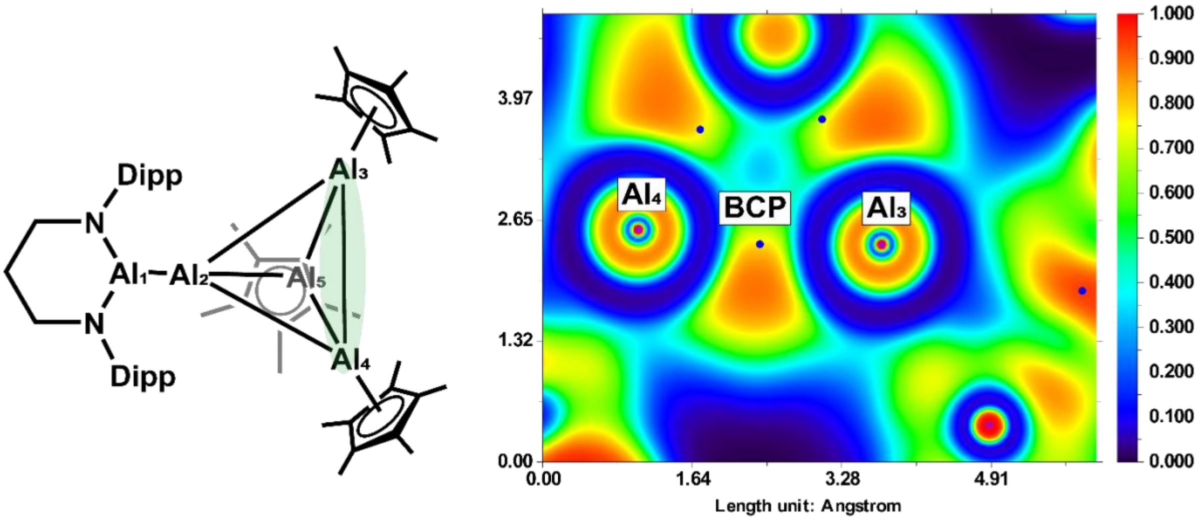

Figure S77. Electron localization function (ELF) map of model **4** plotted in the plane defined by Al3 and Al4 and passing through the Al3–Al4 bond critical point (BCP). At this BCP, the dominant contributions to the electron density arise from MO−160 (33.67%), MO−134 (18.67%), MO−104 (15.25%), MO−146 (11.76%), MO−147 (6.93%), and MO−161 (5.89%).

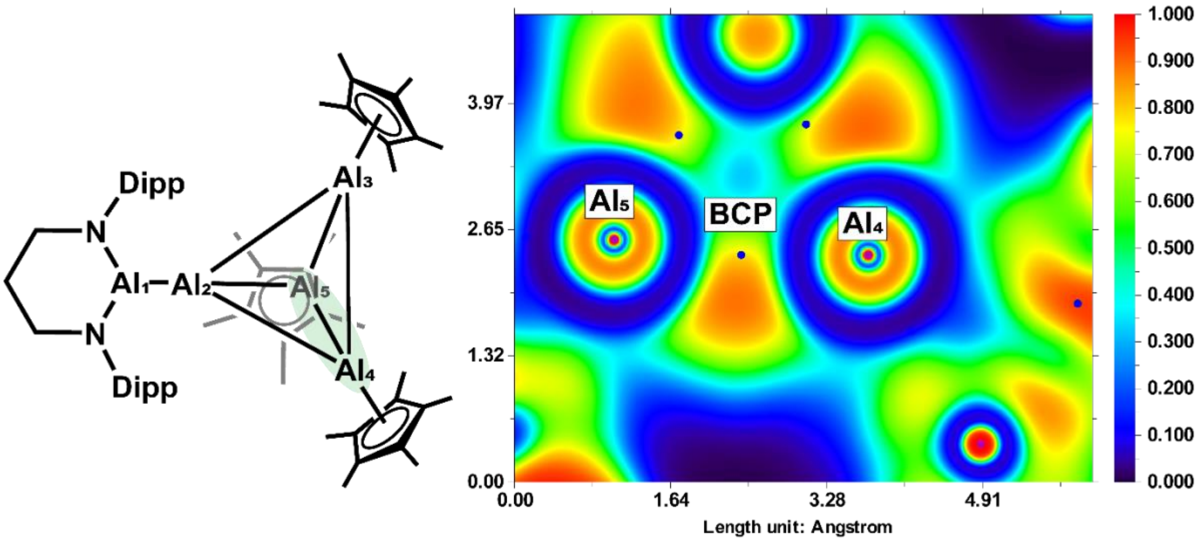

Figure S78. Electron localization function (ELF) map of model **4** plotted in the plane defined by Al4 and Al5 and passing through the Al4–Al5 bond critical point (BCP). At this BCP, the dominant contributions to the electron density arise from MO−159 (22.18%), MO−134 (16.52%), MO−146 (14.91%), MO−104 (14.11%), MO−161 (12.18%) and MO−160 (5.76%).

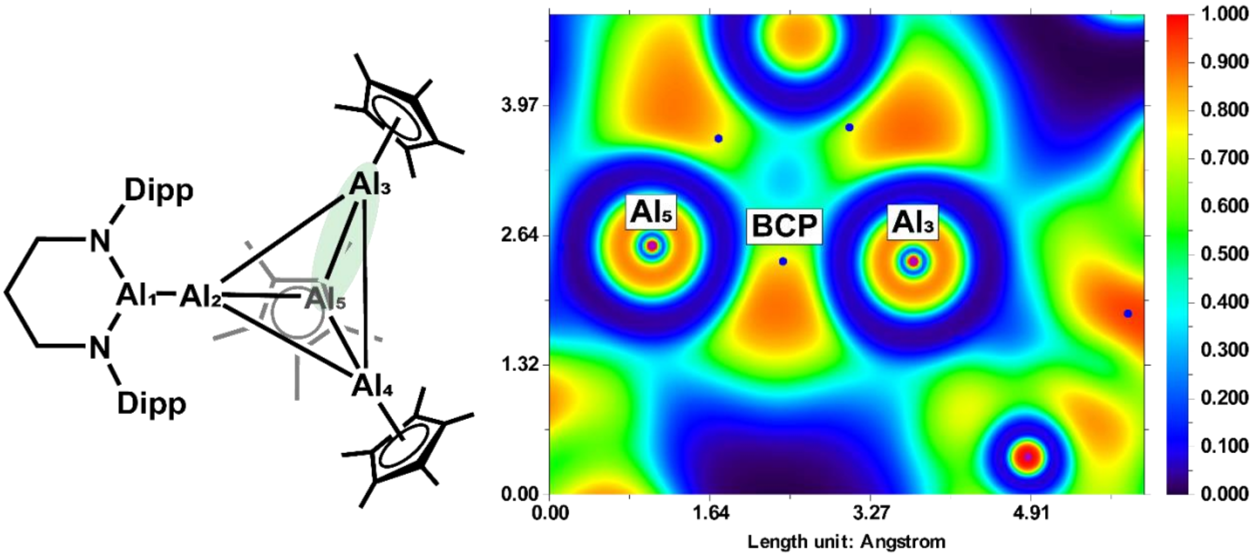

Figure S79. Electron localization function (ELF) map of model **4** plotted in the plane defined by Al3 and Al5 and passing through the Al3–Al5 bond critical point (BCP). At this BCP, the dominant contributions to the electron density arise from MO−159 (21.73%), MO−134 (16.46%), MO−146 (14.89%), MO−104 (14.11%), MO−161 (12.26%), MO−160 (6.21%) and MO−148 (5.19%).

10. Tabulation of NMR data:

Table S1. Comparison of ^{1}H NMR shifts in aluminyl anion K[Al(NDipp)$_2$(CH$_2$)$_3$][13] and Li[Al(NDipp)$_2$(CH$_2$)$_3$] (values reported in ppm)

Aluminyl Anion	C*H*-Ar	C*H*-^{i}Pr	NC*H*$_2$CH$_2$	NCH$_2$C*H*$_2$	C*H*$_3$-^{i}Pr	C*H*$_3$-^{i}Pr
**K[Al(NDipp)$_2$(CH$_2$)$_3$]**	7.00-6.92	4.05	3.41	2.37	1.30	1.22
**Li[Al(NDipp)$_2$(CH$_2$)$_3$]**	7.33-7.25	4.33	3.52	2.48	1.60	1.53

Table S2. *In-situ* relative concentrations of [AlCp^{iPr4}], [K(OB(NDippCH)$_2$)], and [K[Al(OB(NDippCH)$_2$)$_2$]] at different times extracted from ^{1}H NMR spectra. Reaction was run to completion.

Relative concentration	Time = 0 h	Time = 0.5 h	Δ[analyte]/[ref.]
[AlCp^{iPr4}]/[C$_6$H$_6$]	2.3	0.0	-2.3
[K(OB(NDippCH)$_2$)]/[C$_6$H$_6$]	3.9	0.0	-3.9
[K[Al(OB(NDippCH)$_2$)$_2$]]/[C$_6$H$_6$]	0.0	1.8	1.8

Table S3. *In-situ* relative concentrations of [AlCp*], [K^{Dipp}Nacnac], and [Al^{Dipp}Nacnac] at different times extracted from ^{1}H NMR spectra.

Relative concentration	Time = 0.5 h	Time = 4 h	Δ[analyte]/[ref.]
[AlCp*]/[HMDSO]	0.1666	0.1543	-0.0123
[K^{Dipp}Nacnac]/[HMDSO]	0.1307	0.0913	-0.0394
[Al^{Dipp}Nacnac]/[HMDSO]	0.00	0.0033	0.0079

Table S4. *In-situ* relative concentrations of [AlCp^{iPr4}], [K^{Dipp}Nacnac], and [Al^{Dipp}Nacnac] at different times extracted from ^{1}H NMR spectra.

Relative concentration	Time = 0.5 h	Time = 6 h	Δ[analyte]/[ref.]
[AlCp^{iPr4}]/[C$_6$H$_6$]	3.470	2.549	-0.318
[K^{Dipp}Nacnac]/[C$_6$H$_6$]	2.867	1.658	-1.209
[Al^{Dipp}Nacnac]/[C$_6$H$_6$]	0.000	0.177	0.177

Table S5. *In-situ* relative concentrations of [AlCp^{iPr4}], [Li$_2$(NDipp)$_2$(CH$_2$)$_3$(Et$_2$O)$_2$], and [Li[Al(NDipp)$_2$(CH$_2$)$_3$]] at different times. Reaction was run to completion.

Relative concentration	Time = 0.5 h	Time = 15 h	Δ[analyte]/[ref.]
[AlCp^{iPr4}]/[HMDSO]	0.0094	0.0051	-0.0043
[Li$_2$(NDipp)$_2$(CH$_2$)$_3$(Et$_2$O)$_2$]/[HMDSO]	0.0092	0.0000	-0.0092
[Li[Al(NDipp)$_2$(CH$_2$)$_3$]]/[HMDSO]	0.0010	0.0044	0.0034

11. Tabulation of X-ray data

Table S6. X-ray Crystallography Data Collection and Refinement Details.

Identification code (deposition #)	**[(Li(NDipp)$_2$C$_5$H$_9$)$_2$ THF] (2531053)**	**[(Al(NDipp)$_2$C$_5$H$_9$)$_2$ C$_7$H$_8$]·C$_7$H$_8$ (2531050)**	**[(Al(NDipp)$_2$C$_5$H$_9$)$_2$ C$_6$D$_6$]·C$_6$H$_{14}$ (2531049)**
Empirical formula	$C_{62}H_{94}Li_2N_4O$	$C_{71.99}H_{101.98}Al_2N_4$	$C_{70}H_{100}Al_2D_6N_4$
Formula weight	925.29	1077.38	1063.58
Temperature/K	110.00	110.00	110.00
Crystal system	triclinic	monoclinic	monoclinic
Space group	P-1	Pn	Pn
a/Å	10.8117(6)	12.4020(5)	12.3950(7)
b/Å	14.0773(8)	10.7732(4)	10.6767(7)
c/Å	19.9743(11)	24.5107(10)	24.4982(14)
α/°	74.173(3)	90	90
β/°	86.035(3)	99.104(2)	98.873(3)
γ/°	86.570(4)	90	90
Volume/Å^3	2915.2(3)	3233.6(2)	3203.2(3)
Z	2	2	2
ρ_{calc}g/cm^3	1.054	1.107	1.103
μ/mm^{-1}	0.456	0.722	0.718
F(000)	1016.0	1176.0	1160.0
Crystal size/mm^3	0.366 × 0.341 × 0.293	0.544 × 0.253 × 0.215	0.238 × 0.191 × 0.185
Radiation	CuKα (λ = 1.54178)	CuKα (λ = 1.54178)	CuKα (λ = 1.54178)
2Θ range for data collection/°	6.532 to 137.216	7.304 to 143.668	7.57 to 137.422
Index ranges	-13 ≤ h ≤ 13, -16 ≤ k ≤ 16, -24 ≤ l ≤ 24	-14 ≤ h ≤ 15, -13 ≤ k ≤ 13, -30 ≤ l ≤ 29	-14 ≤ h ≤ 14, -11 ≤ k ≤ 12, -29 ≤ l ≤ 29
Reflections collected	24662	52816	36651
Independent reflections	10348 [R_{int} = 0.0405, R_{sigma} = 0.0484]	11872 [R_{int} = 0.0451, R_{sigma} = 0.0353]	11332 [R_{int} = 0.0937, R_{sigma} = 0.0735]
Data/restraints/parameters	10348/1/652	11872/559/517	11332/2/651
Goodness-of-fit on F^2	1.039	1.051	1.066
Final R indexes [I>=2σ (I)]	R_1 = 0.0938, wR_2 = 0.2276	R_1 = 0.0834, wR_2 = 0.2160	R_1 = 0.0773, wR_2 = 0.1811
Final R indexes [all data]	R_1 = 0.1368, wR_2 = 0.2717	R_1 = 0.1050, wR_2 = 0.2485	R_1 = 0.1148, wR_2 = 0.2085
Largest diff. peak/hole / e Å^{-3}	0.36/-0.29	0.68/-0.66	0.40/-0.33
Flack parameter	N/A	0.58(12)	0.42(10)

Identification code (deposition #)	**[Al(N_2Dipp$_2$$C_3H_6$)($Al_4Cp^*_3$)]·0.5$C_6H_6$ (2531052)**	**[AlNTMSDippAlCp*]$_2$·C_6H_{14} (2531051)**
Empirical formula	$C_{60}H_{88}Al_5N_2$	$C_{56.04}H_{96.09}Al_4N_2Si_2$
Formula weight	972.22	961.87
Temperature/K	130.00	110.00
Crystal system	triclinic	monoclinic
Space group	P-1	C2/c
a/Å	10.9764(9)	16.1442(9)
b/Å	13.9661(12)	16.3268(10)
c/Å	21.8278(19)	22.4903(15)
α/°	103.500(5)	90
β/°	93.255(5)	96.065(4)
γ/°	111.999(4)	90
Volume/Å^3	2977.9(4)	5894.9(6)
Z	2	4
ρ_{calc}g/cm^3	1.084	1.084
μ/mm^{-1}	1.135	1.375
F(000)	1054.0	2105.0
Crystal size/mm^3	0.225 × 0.193 × 0.107	0.367 × 0.159 × 0.136
Radiation	CuKα (λ = 1.54178)	CuKα (λ = 1.54178)
2Θ range for data collection/°	7.1 to 136.754	7.722 to 136.898
Index ranges	-13 ≤ h ≤ 13, -16 ≤ k ≤ 16, -26 ≤ l ≤ 26	-19 ≤ h ≤ 19, 0 ≤ k ≤ 19, 0 ≤ l ≤ 26
Reflections collected	26182	5270
Independent reflections	10556 [R_{int} = 0.0460, R_{sigma} = 0.0513]	5270 [R_{int} = N/A, R_{sigma} = 0.0809]
Data/restraints/parameters	10556/0/627	5270/27/332
Goodness-of-fit on F^2	1.072	1.081
Final R indexes [I>=2σ (I)]	R_1 = 0.0941, wR_2 = 0.1861	R_1 = 0.0803, wR_2 = 0.1915
Final R indexes [all data]	R_1 = 0.1277, wR_2 = 0.2101	R_1 = 0.1142, wR_2 = 0.2232
Largest diff. peak/hole / e Å^{-3}	0.67/-0.45	0.52/-0.55
Flack parameter	N/A	N/A

12. Tabulation of QTAIM critical points

Table S7. Real space functions at critical points.

Structure **3**							
	Bond Length (Å) Calc./Exp.	Electron density ρ(r) (e·bohr⁻³)	Energy density H(r) (Eh·bohr⁻³)	Laplacian of electron density $\nabla^2\rho(r)$ (e·bohr⁻⁵)	Kinetic energy density G(r) (Eh·bohr⁻³)	Potential energy density V(r) (Eh·bohr⁻³)	DI
CpAl3-Al4Cp	2.754/2.785	0.0356	-0.0100	-0.0102	0.0075	-0.0175	0.5520
NAl1-Al2N	2.582/2.583	0.0452	-0.0169	-0.0413	0.0066	-0.0235	0.6472
CpAl1-Al4N	2.578/2.650	0.0465	-0.0199	-0.0258	0.0134	-0.0332	0.6092
CpAl1-Al4N	2.578/2.650	0.0465	-0.0199	-0.0257	0.0134	-0.0333	0.6080
CpAl2-Al4N	2.669/2.651	0.0407	-0.0136	-0.0269	0.0068	-0.0204	0.5821
CpAl2-Al4N	2.669/2.651	0.0407	-0.0136	-0.0269	0.0069	-0.0204	0.5815
RCP (3,+1)							
Al_3Cp_2N		0.0332	-0.0063	0.0104	0.0089	-0.0153	
Al_3Cp_2N		0.0332	-0.0064	0.0103	0.0089	-0.0153	
Al_3CpN_2		0.0348	-0.0072	0.0112	0.0100	-0.0172	
Al_3CpN_2		0.0348	-0.0073	0.0111	0.0100	-0.0173	
CCP (3,+3)							
Al_4		0.0270	-0.0028	0.0537	0.0162	-0.0190	
Structure **4**							
Al1-Al4	2.710/2.710	0.0480	-0.0204	-0.0342	0.0118	-0.0322	0.6287
AlAl2-Al4Cp	2.600/2.643	0.0458	-0.0190	-0.0333	0.0106	-0.0296	0.6425
AlAl2-Al5Cp	2.632/2.660	0.0442	-0.0160	-0.0373	0.0067	-0.0227	0.6100
AlAl2-Al3Cp	2.601/2.655	0.0457	-0.0189	-0.0336	0.0105	-0.0294	0.6407
CpAl3-Al4Cp	2.671/2.721	0.0394	-0.0131	-0.0246	0.0069	-0.0200	0.5714
CpAl4-Al5Cp	2.717/2.722	0.0370	-0.0112	-0.0159	0.0073	-0.0185	0.5700

CpAl3-Al5Cp	2.668/2.694	0.0397	-0.0132	-0.0251	0.0069	-0.0201	0.5702
RCP (3,+1)							
Al-Al_3Cp_2		0.0347	-0.0072	0.0102	0.0097	-0.0169	
Al-Al_3Cp_2		0.0347	-0.0072	0.0086	0.0094	-0.0166	
Al-Al_3Cp_2		0.0347	-0.0072	0.0102	0.0097	-0.0169	
Al_3Cp_3		0.0313	-0.0054	0.0134	0.0088	-0.0142	
CCP (3,+3)							
Al_4		0.0268	-0.0027	0.0523	0.0158	-0.0184	

13. DFT coordinates and details

13.1 Model structure 3

Converged coordinates of model **3**:

68

Coordinates from ORCA-job orcaJob E -2667.515205362268 Hartrees

B3LYP/def2-SVP, aux. def2/J, dispersion correction: D3BJ

Si	5.69493527681744	2.08690557405846	8.70691786773547
Al	6.95510116794225	4.29503585935429	6.7606381942156
Al	6.26743669888694	6.12586438849012	4.94419779875206
N	6.6257220778352	3.52795601175308	8.4121645462317
C	7.24159077260659	4.24683200370642	9.48699176160928
C	4.86850176901643	7.91440458676859	5.15712443087598
C	4.07121322818531	6.77596111849386	4.84819019788262
C	5.42622803805467	7.20599071220264	3.03543829602257
C	6.55174572329245	5.31499000435478	10.1099178710722
C	5.70817679925463	8.17588351003997	4.03356569771916
C	9.16988867744317	4.67710860384058	10.9004240908444
C	4.42259835414306	6.33812699331246	3.53960855388787
C	5.16426429745815	5.68799250971098	9.65642536294797
C	8.488303018176	5.71959144725739	11.5303992771935
C	9.32765048099449	2.83966892775378	9.1733214103825
C	8.56748846302706	3.93461384421205	9.87581162738151
C	7.18908307495245	6.03530405895151	11.1298888607364
Si	9.30241676325552	2.14350125347303	2.4328677411719
Al	8.02208390547246	4.31397203123132	4.40944727689063
Al	8.69354834181859	6.12880650170534	6.24837084367903
N	8.35173493714276	3.56702892325646	2.7488744067368
C	7.71708329425105	4.28492501875392	1.68429144107214
C	10.0862170250128	7.92436236166272	6.05711805151453
C	10.8861791795622	6.78799081728698	6.36677614842692
C	9.51446371756151	7.20237800039272	8.17054697127499
C	8.38775593736528	5.36579495281534	1.06233851476942
C	9.23604678141291	8.17603357854137	7.1750754121544
C	5.77204050421861	4.69771844721565	0.28865351652254
C	10.5261830608639	6.34185343583865	7.6702177641094
C	9.77318486538624	5.75573559526443	1.50782292097124
C	6.43497287877987	5.75239103772733	-0.34089050141537
C	5.65241302588725	2.84856945262893	2.00643695990381

C	6.39231576312609	3.95745822854643	1.3041927364363
C	7.73303428869931	6.08305299575694	0.05125416952753
H	5.22372132922761	1.61020798779867	7.37097692219989
H	4.50050474082682	2.32659225651888	9.57758640027587
H	6.48528878500281	0.99575815908943	9.35888366819652
H	4.84487493003155	8.47968463321389	6.08784590168257
H	3.33652674096126	6.31297042660038	5.50546498376957
H	5.93112340453334	7.09685582195553	2.07666724712545
H	6.44761678224127	8.97244027962296	3.96198086776129
H	10.1918422388398	4.43246860892215	11.2035292920573
H	4.01753315428713	5.47122339171645	3.02032529803862
H	4.47274433658088	4.83327136397747	9.71551528184865
H	5.16934335123175	6.01372744962152	8.60154467332242
H	4.75648234491556	6.50787575115013	10.2655658707346
H	8.97122086785035	6.28902848776853	12.3284088201688
H	9.45047655014577	3.06480754367098	8.09987194608098
H	8.79917021444272	1.87540634241211	9.22791294068104
H	10.32798318342	2.70977444211925	9.61183665682041
H	6.65397861949737	6.85748175174594	11.6134546123431
H	8.52054029079226	1.04034799489494	1.79079879470257
H	9.80538751038443	1.67019028236524	3.75836119812071
H	10.4756667661272	2.40937424397975	1.54150864546904
H	10.1150776337862	8.4948369071875	5.12971796410226
H	11.628333400548	6.33173941155782	5.7131858311922
H	9.00216230492106	7.08580237181755	9.12452060963143
H	8.4924915586093	8.96893575304324	7.24465858139083
H	4.75100111498658	4.44128731181923	-0.00772806545214
H	10.9310223627454	5.47417170122445	8.1883284441149
H	10.1653745357108	6.58378523683262	0.89947918928914
H	10.476059289182	4.91100945559049	1.44041550358621
H	9.7708274784333	6.07705248602976	2.56407411913708
H	5.9383928132741	6.31971685415942	-1.13199201409781
H	4.65268206275505	2.70367687382468	1.57126355454964
H	5.52975607093015	3.06923510244412	3.08089780650543
H	6.196377460805	1.89326541433086	1.94764534494551
H	8.25331358406616	6.91492511463275	-0.43191710953434

Vibrational frequencies of model 3:

IR SPECTRUM

Mode	Frequency cm^-1	epsilon L/(mol*cm)	Intensity km/mol	T**2 a.u.	TX,TY,TZ a.u.
6:	7.00	0.000006	0.03	0.000283	(0.0005140.000141-0.016819)
7:	11.45	0	0	0.000003	(0.00003-0.0013620.000919)
8:	21.86	0.000009	0.05	0.000128	(0.0000410.011330.000121)
9:	29.57	0.000001	0.01	0.000014	(0.00007-0.0001610.003741)
10:	32.64	0	0	0.000001	(0.0001970.000682-0.000975)
11:	33.84	0	0	0.000001	(-0.0000150.000413-0.000710)
12:	34.37	0.000006	0.03	0.000056	(-0.0005750.000230.007462)
13:	44.38	0.000002	0.01	0.000012	(-0.000108-0.0034770.000315)
14:	50.68	0.000031	0.16	0.000192	(-0.000349-0.0001060.013848)
15:	54.32	0.000004	0.02	0.000022	(-0.0000390.0047270.000304)
16:	60.88	0.000067	0.34	0.000345	(-0.001885-0.0002250.018485)
17:	61.91	0.000001	0	0.000003	(0.000121-0.000127-0.001693)
18:	67.07	0.000041	0.21	0.000189	(0.0040160.0001390.013157)
19:	90.51	0.00002	0.1	0.000068	(0.000301-0.0000850.008266)
20:	94.54	0.000014	0.07	0.000045	(0.0000140.006683-0.000008)
21:	100.37	0.000016	0.08	0.000051	(-0.006867-0.0001330.001970)
22:	112.00	0.00001	0.05	0.000027	(-0.0000840.0051730.000111)
23:	113.16	0	0	0	(0.000355-0.000311-0.000013)
24:	120.53	0.000018	0.09	0.000046	(0.0066690.000059-0.001077)
25:	120.94	0.000027	0.13	0.000068	(0.0002880.008244-0.000667)
26:	123.50	0.000038	0.19	0.000096	(0.004172-0.000681-0.008859)
27:	127.14	0.000009	0.05	0.000023	(-0.004151-0.000098-0.002438)
28:	128.99	0.000002	0.01	0.000004	(0.0004120.002057-0.000208)
29:	156.06	0.000002	0.01	0.000003	(-0.0005830.0000150.001659)
30:	160.00	0.000002	0.01	0.000005	(0.00006-0.002164-0.000003)
31:	163.74	0.000004	0.02	0.000008	(-0.0008110.002531-0.000989)
32:	163.96	0.000025	0.12	0.000047	(-0.003955-0.000629-0.005557)
33:	166.57	0.000006	0.03	0.00001	(-0.0007140.0028040.001430)
34:	167.05	0.000049	0.25	0.000091	(0.0009360.000728-0.009487)
35:	188.59	0.000968	4.89	0.001601	(0.0138750.0002010.037530)
36:	195.61	0.000284	1.44	0.000454	(0.000192-0.0212960.000423)
37:	201.39	0.000802	4.05	0.001243	(0.015618-0.0002290.031608)
38:	203.66	0.000038	0.19	0.000058	(-0.000045-0.007643-0.000238)

39:	217.79	0.000131	0.66	0.000188	(-0.0137250.000064-0.000087)
40:	237.58	0.000042	0.21	0.000056	(0.000011-0.007454-0.000023)
41:	239.34	0.000021	0.11	0.000028	(-0.004084-0.000113-0.003329)
42:	240.32	0.000126	0.63	0.000163	(0.000077-0.012766-0.000077)
43:	257.82	0.002895	14.63	0.003504	(0.0474450.0000590.035400)
44:	264.72	0.000242	1.22	0.000285	(0.0004740.0168840.000217)
45:	288.49	0.002177	11	0.002355	(0.000108-0.048525-0.000610)
46:	303.36	0.00331	16.73	0.003405	(-0.0127420.000161-0.056942)
47:	309.58	0.00171	8.64	0.001723	(-0.0001770.0415130.000131)
48:	319.19	0.004563	23.06	0.004461	(-0.038263-0.0003630.054743)
49:	328.57	0.000976	4.93	0.000927	(0.000169-0.030439-0.000211)
50:	350.37	0.000335	1.69	0.000298	(-0.017201-0.0004160.001530)
51:	354.88	0.000314	1.59	0.000276	(-0.0002730.0166140.000066)
52:	404.08	0.037185	187.92	0.028717	(0.157640.0006550.062179)
53:	417.36	0.013567	68.56	0.010144	(-0.0008930.1007140.000248)
54:	424.42	0.023441	118.46	0.017235	(0.051367-0.0001550.120816)
55:	471.58	0.012188	61.59	0.008065	(-0.0005370.0898040.000561)
56:	498.20	0.000035	0.18	0.000022	(0.004609-0.0000820.000839)
57:	498.66	0.000015	0.08	0.000009	(0.0000220.0030520.000086)
58:	524.45	0.000017	0.09	0.00001	(-0.002274-0.001829-0.001294)
59:	524.50	0.000019	0.1	0.000011	(0.001453-0.0029240.000853)
60:	555.90	0.015023	75.92	0.008434	(0.0230210.000523-0.088900)
61:	561.30	0.000193	0.98	0.000107	(-0.0083410.0014290.005974)
62:	561.44	0.000105	0.53	0.000058	(-0.000297-0.007633-0.000251)
63:	562.29	0.000912	4.61	0.000506	(0.0004460.02248-0.000636)
64:	566.96	0.001425	7.2	0.000785	(0.0025570.00001-0.027893)
65:	581.89	0.000174	0.88	0.000094	(0.0000160.0096740.000025)
66:	600.54	0	0	0	(-0.0001210.000078-0.000334)
67:	600.98	0.000001	0.01	0.000001	(-0.000031-0.000807-0.000022)
68:	606.67	0.000008	0.04	0.000004	(0.0011620.000059-0.001589)
69:	607.50	0.000005	0.02	0.000002	(-0.0000410.001569-0.000023)
70:	649.34	0.006485	32.77	0.003117	(-0.025403-0.0001980.049713)
71:	657.18	0.002587	13.07	0.001228	(0.00014-0.0350470.000036)
72:	711.21	0.008287	41.88	0.003636	(-0.058828-0.0108580.007578)
73:	711.40	0.004057	20.5	0.00178	(0.015941-0.039014-0.001851)
74:	754.42	0.005942	30.03	0.002458	(0.003013-0.0002990.049484)
75:	755.70	0.000013	0.07	0.000005	(0.00015-0.00229-0.000351)
76:	763.08	0.005068	25.61	0.002073	(0.020054-0.00005-0.040872)
77:	764.51	0.002881	14.56	0.001176	(-0.000096-0.0342930.000197)

78:	786.37	0.007232	36.55	0.00287	(0.007902-0.003959-0.052839)
79:	786.46	0.001885	9.53	0.000748	(-0.001303-0.0259830.008431)
80:	818.03	0.012204	61.67	0.004655	(-0.063817-0.000015-0.024142)
81:	819.32	0.016365	82.7	0.006233	(-0.078055-0.000928-0.011818)
82:	821.54	0.012591	63.63	0.004783	(-0.0013980.0691430.000063)
83:	823.26	0.004287	21.66	0.001625	(-0.0008760.040301-0.000002)
84:	831.67	0.047767	241.4	0.017923	(-0.111567-0.001874-0.073978)
85:	836.27	0.034751	175.62	0.012968	(0.001624-0.1138650.000161)
86:	854.02	0.00004	0.2	0.000015	(-0.0021270.0031440.000455)
87:	854.12	0.00015	0.76	0.000055	(-0.000783-0.0073580.000184)
88:	859.62	0.000001	0	0	(0.000437-0.0001220.000115)
89:	859.64	0.000008	0.04	0.000003	(-0.001601-0.000043-0.000605)
90:	891.19	0.089699	453.3	0.031409	(0.0215560.001418-0.175906)
91:	898.13	0.001328	6.71	0.000461	(0.0003940.0214360.001353)
92:	911.63	0.000002	0.01	0.000001	(-0.0000490.000664-0.000431)
93:	912.03	0.000057	0.29	0.00002	(0.0006250.0001780.004387)
94:	916.22	0.000049	0.25	0.000017	(0.0012470.0038540.000505)
95:	916.23	0.000031	0.16	0.000011	(0.001689-0.0026470.000887)
96:	931.99	0.002828	14.29	0.000947	(-0.0292610.001298-0.009439)
97:	932.39	0.001497	7.57	0.000501	(-0.003373-0.0221210.000684)
98:	932.54	0.008345	42.17	0.002793	(0.049779-0.003505-0.017391)
99:	932.77	0.002967	15	0.000993	(0.004510.031153-0.001386)
100:	934.80	0.008266	41.77	0.00276	(-0.0223190.0001880.047554)
101:	936.08	0.000165	0.83	0.000055	(-0.001862-0.0067540.002400)
102:	939.03	0.075703	382.57	0.025158	(0.1053980.000611-0.118528)
103:	949.85	0.063373	320.26	0.02082	(-0.0014150.1442810.001199)
104:	955.45	0.02467	124.67	0.008057	(0.070168-0.0108750.054916)
105:	956.10	0.048323	244.2	0.015772	(0.0059790.1253260.005465)
106:	994.87	0.01692	85.51	0.005307	(-0.0572780.0038210.044854)
107:	995.73	0.03026	152.92	0.009484	(0.002460.097339-0.001620)
108:	998.86	0.00314	15.87	0.000981	(-0.0307250.004376-0.004227)
109:	999.03	0.007494	37.87	0.002341	(0.0029120.0482920.000624)
110:	1001.79	0.001045	5.28	0.000326	(-0.0001140.003572-0.017685)
111:	1001.86	0.000368	1.86	0.000115	(-0.000356-0.007-0.008096)
112:	1024.32	0.002279	11.52	0.000694	(0.023998-0.001011-0.010831)
113:	1024.77	0.001388	7.02	0.000423	(0.0009340.020427-0.002143)
114:	1025.02	0.003242	16.39	0.000987	(-0.0007480.0009130.031397)
115:	1025.63	0.00097	4.9	0.000295	(0.0003590.017172-0.000011)
116:	1048.90	0.000017	0.09	0.000005	(0.001955-0.001121-0.000223)

117:	1048.93	0.000017	0.09	0.000005	(0.0017710.001422-0.000027)
118:	1051.47	0.000083	0.42	0.000025	(-0.001116-0.0007930.004766)
119:	1051.52	0.000046	0.23	0.000014	(-0.0002750.0035950.000759)
120:	1052.34	0.000114	0.58	0.000034	(-0.000008-0.001531-0.005612)
121:	1052.39	0.000058	0.29	0.000017	(0.000003-0.0033740.002399)
122:	1068.26	0.000165	0.83	0.000048	(0.006937-0.000094-0.000041)
123:	1069.80	0.000043	0.22	0.000013	(-0.0000360.0035360.000036)
124:	1074.61	0.000227	1.15	0.000066	(-0.002963-0.0074250.001396)
125:	1074.66	0.000614	3.1	0.000178	(0.011981-0.00149-0.005704)
126:	1115.19	0.005036	25.45	0.001409	(-0.019118-0.010267-0.030631)
127:	1115.26	0.002679	13.54	0.00075	(0.009594-0.0209820.014746)
128:	1147.63	0.003162	15.98	0.00086	(0.0237410.0001940.017206)
129:	1148.52	0.000766	3.87	0.000208	(-0.0003720.014422-0.000160)
130:	1176.97	0.000168	0.85	0.000045	(0.005284-0.003949-0.001001)
131:	1177.04	0.000163	0.82	0.000043	(-0.004891-0.0043050.000870)
132:	1236.42	0.0305	154.13	0.007698	(0.035703-0.0053210.079969)
133:	1236.99	0.006591	33.31	0.001663	(0.0043470.0390090.011059)
134:	1267.19	0.000002	0.01	0	(-0.000235-0.000327-0.000481)
135:	1267.26	0.000002	0.01	0	(0.000215-0.0003850.000420)
136:	1278.75	0.000026	0.13	0.000006	(-0.001819-0.000067-0.001776)
137:	1278.86	0.000025	0.13	0.000006	(0.0000060.002477-0.000034)
138:	1287.25	0.009824	49.65	0.002382	(0.021777-0.0007470.043667)
139:	1287.55	0.002182	11.03	0.000529	(0.0005030.0229320.001675)
140:	1339.76	0.000023	0.12	0.000005	(-0.001297-0.0015490.001162)
141:	1339.81	0.000024	0.12	0.000005	(0.001297-0.001557-0.001171)
142:	1396.66	0.000886	4.48	0.000198	(-0.013179-0.0005730.004897)
143:	1396.77	0.000684	3.46	0.000153	(-0.0005850.0123460.000183)
144:	1399.16	0.000154	0.78	0.000034	(-0.005356-0.0000880.002359)
145:	1399.94	0.000007	0.04	0.000002	(-0.0000270.001243-0.000019)
146:	1401.34	0.001607	8.12	0.000358	(-0.0082770.000093-0.017009)
147:	1401.79	0.000008	0.04	0.000002	(-0.0000030.001309-0.000111)
148:	1402.41	0.000225	1.14	0.00005	(0.002753-0.0000040.006518)
149:	1403.61	0.00003	0.15	0.000007	(-0.000031-0.0025830.000020)
150:	1438.79	0.002007	10.14	0.000435	(-0.0084840.014821-0.011986)
151:	1438.81	0.003437	17.37	0.000745	(0.0146230.0083950.021474)
152:	1452.26	0.000576	2.91	0.000124	(0.0085510.000035-0.007117)
153:	1452.49	0.000743	3.76	0.00016	(-0.000047-0.0126350.000074)
154:	1453.43	0.000376	1.9	0.000081	(0.0056520.001972-0.006708)
155:	1453.56	0.000463	2.34	0.000099	(0.001194-0.009773-0.001596)

156:	1456.12	0.000586	2.96	0.000125	(-0.0111210.0003030.001315)
157:	1456.22	0.000329	1.66	0.00007	(0.0003420.008387-0.000090)
158:	1469.29	0.000536	2.71	0.000114	(-0.005670-0.000057-0.009040)
159:	1470.28	0.001364	6.89	0.000289	(-0.0002640.0170080.000366)
160:	1471.20	0.002803	14.16	0.000594	(-0.013874-0.0003260.020047)
161:	1471.81	0.000095	0.48	0.00002	(-0.000332-0.0044310.000579)
162:	1490.61	0.021488	108.59	0.004499	(0.025087-0.0000950.062203)
163:	1490.97	0.003611	18.25	0.000756	(-0.0002600.0274880.000404)
164:	1506.83	0.00146	7.38	0.000302	(0.0119350.01257-0.001352)
165:	1506.84	0.00232	11.73	0.000481	(-0.0206030.0071330.002273)
166:	1633.57	0.000087	0.44	0.000017	(0.003731-0.001543-0.000503)
167:	1633.57	0.000063	0.32	0.000012	(-0.002244-0.002630.000335)
168:	1644.88	0.001187	6	0.000225	(-0.0014910.000225-0.014930)
169:	1644.99	0.000067	0.34	0.000013	(-0.000322-0.000726-0.003487)
170:	2179.24	0.028578	144.42	0.004092	(0.058404-0.020929-0.015597)
171:	2179.67	0.01943	98.19	0.002782	(-0.039491-0.0337980.008940)
172:	2193.24	0.048944	247.34	0.006964	(0.0246310.004947-0.079579)
173:	2194.00	0.027886	140.92	0.003966	(0.00308-0.06269-0.005176)
174:	2206.22	0.022519	113.8	0.003185	(-0.029528-0.01362-0.046129)
175:	2206.48	0.011863	59.95	0.001678	(-0.0120510.035017-0.017502)
176:	3018.58	0.00331	16.73	0.000342	(-0.0158740.007861-0.005331)
177:	3018.71	0.003155	15.94	0.000326	(0.0151260.0085760.004877)
178:	3021.94	0.002296	11.6	0.000237	(0.0093170.008772-0.008562)
179:	3022.24	0.002367	11.96	0.000244	(-0.0098130.0086710.008542)
180:	3090.97	0.005371	27.14	0.000542	(0.0140190.000682-0.018580)
181:	3091.20	0.002749	13.89	0.000278	(-0.0100150.0004410.013305)
182:	3093.19	0.004351	21.99	0.000439	(-0.006995-0.007132-0.018415)
183:	3093.27	0.003482	17.59	0.000351	(0.002915-0.0168580.007652)
184:	3123.64	0.002566	12.97	0.000256	(0.014449-0.0011980.006794)
185:	3123.91	0.002217	11.2	0.000221	(-0.013477-0.001578-0.006109)
186:	3124.95	0.002776	14.03	0.000277	(-0.0029500.0098460.013097)
187:	3125.09	0.002826	14.28	0.000282	(0.0016510.013825-0.009398)
188:	3162.44	0.000395	2	0.000039	(-0.0007620.000634-0.006162)
189:	3162.47	0.000208	1.05	0.000021	(-0.000469-0.000893-0.004421)
190:	3169.05	0.003251	16.43	0.00032	(-0.013802-0.0110910.002579)
191:	3169.08	0.003576	18.07	0.000352	(0.015911-0.00952-0.002880)
192:	3189.36	0.006839	34.56	0.000669	(0.009433-0.0044390.023675)
193:	3189.40	0.002312	11.68	0.000226	(0.003920.0101790.010354)
194:	3226.67	0.000087	0.44	0.000008	(-0.000884-0.0006560.002681)

195:	3226.80	0.000147	0.75	0.000014	(0.001152-0.000503-0.003561)
196:	3230.78	0.00069	3.49	0.000067	(0.0026680.001969-0.007461)
197:	3230.84	0.000333	1.68	0.000032	(-0.0013600.0038840.003902)
198:	3241.98	0.001133	5.73	0.000109	(-0.003778-0.0038670.008934)
199:	3242.11	0.000829	4.19	0.00008	(0.002875-0.004932-0.006874)
200:	3242.92	0.000014	0.07	0.000001	(-0.0008720.000417-0.000645)
201:	3242.97	0.000022	0.11	0.000002	(-0.000425-0.001361-0.000223)
202:	3253.09	0.000739	3.74	0.000071	(0.002964-0.007408-0.002691)
203:	3253.14	0.000647	3.27	0.000062	(-0.004949-0.004370.004294)

GIBBS FREE ENERGY

The Gibbs free energy is G = H - T*S

Total enthalpy ... -2666.94927811 Eh

Total entropy correction ... -0.10836405 Eh -68.00 kcal/mol

Final Gibbs free energy ... -2667.05764216 Eh

For completeness - the Gibbs free energy minus the electronic energy

G-E(el) ... 0.45756245 Eh 287.12 kcal/mol

13.2 Model structure 4

Converged coordinates of model 4:

80

Coordinates from ORCA-job orcaJob E -2638.527616205628 Hartrees

B3LYP/def2-SVP, aux. def2/J, dispersion correction: D3BJ

Al	1.824793004	7.179718377	5.180733956
Al	0.361506799	4.997740147	5.029032869
Al	-0.448577851	7.133056379	3.784335808
Al	-0.490494073	6.967955734	6.495710594
C	3.879984707	7.553487996	4.109982083
C	3.78365308	7.594913478	6.407043103
C	-2.139793586	6.825340783	2.159826075
C	-0.897364783	6.845420534	1.476467268
C	3.383322354	8.826369308	4.507119243
C	4.133810021	6.795500806	5.284772503
C	3.3256122	8.852373245	5.924815872
C	-0.30632551	8.122897628	1.678278788
C	-2.321329719	8.088946172	2.788153517
C	-2.253672691	6.45321227	7.993414784
C	-1.286028071	8.54123575	7.983478756
C	-2.406995305	7.789695131	7.530908718
C	-0.438155539	7.661708442	8.714706343
C	-1.188978663	8.895944168	2.483982063
C	-1.040160825	6.373451437	8.722510875
H	-3.232997759	8.171740736	6.932512843
H	-2.926080137	5.62253408	7.789023271
H	-1.1009043	9.596629796	7.788795466
H	0.510630885	7.928448247	9.178378824
H	-0.62961324	5.468410744	9.16748811
H	2.963972619	9.678365419	6.535474225
H	3.075236196	9.629403502	3.839135123
H	4.027921639	7.214361451	3.085500457
H	4.501659718	5.771180323	5.318499774
H	3.846145716	7.293103773	7.451773742
H	-3.173359513	8.386389189	3.397910108
H	-1.018788917	9.916197773	2.824822329
H	0.658762253	8.450381425	1.294047582
H	-0.462929989	6.008536131	0.931836847
H	-2.814905775	5.974483188	2.224166292

Al	-0.13107097	2.338110089	4.862194354
N	-0.508108898	1.412583097	3.346006191
N	-0.567073478	1.232226711	6.234573441
C	-0.672351363	1.697007502	7.573334958
C	-0.565816519	2.041007325	2.072797919
C	0.363998276	1.43173399	8.500226511
C	0.501784326	1.886700256	1.155984942
C	-0.942651219	0.016577306	3.398325392
C	-1.800866276	2.452649583	7.971956318
C	-1.679223435	2.845915265	1.731938212
C	1.588143893	0.667994914	8.066035183
C	-1.883734176	2.916921033	9.292304848
C	0.249844113	1.914472605	9.811012978
C	-0.867190469	2.650175458	10.21030225
C	-0.998494196	-0.144483881	5.99016414
C	-0.473837587	-0.706441339	4.66496229
C	-2.893573505	2.771672958	6.981509505
C	1.710978752	1.071902372	1.535939728
C	-1.716899004	3.467870068	0.476031179
C	-0.670002658	3.310240206	-0.43305797
C	-2.803895334	3.048981725	2.717281787
C	0.432717596	2.526636729	-0.088922382
H	-3.683309087	3.493082308	2.227015651
H	-2.502663105	3.729455554	3.533830336
H	-3.110257141	2.104762268	3.190767874
H	2.492347153	1.137153053	0.76463963
H	1.462116625	0.00700269	1.675712841
H	2.131580949	1.417312673	2.494099581
H	-3.784509851	3.168582646	7.490969644
H	-3.192561589	1.886932532	6.400313742
H	-2.564467751	3.532504786	6.2514782
H	2.331438628	0.616319927	8.875066807
H	2.057369437	1.141152997	7.188146001
H	1.343277083	-0.363532623	7.764279382
H	-2.758884535	3.494611808	9.602946571
H	-0.945162187	3.017003559	11.23707876
H	1.053127684	1.71260773	10.52546201
H	1.260345963	2.410708482	-0.794698726
H	-0.712976606	3.799968685	-1.409398291
H	-2.580420185	4.083599342	0.209107954
H	-2.107374292	-0.223355209	6.001933638

H	-0.648707703	-0.80101525	6.808941596
H	0.631355108	-0.699441399	4.689360564
H	-0.786782962	-1.761591711	4.592348744
H	-0.554232147	-0.532898562	2.520276322
H	-2.049612301	-0.05884382	3.327911193

Vibrational frequencies of model 4:

IR SPECTRUM

Mode	Frequency cm^-1	epsilon L/(mol*cm)	Intensity km/mol	T**2 a.u.	TX,TY,TZ a.u.
6:	-11.65	***imaginary mode***			
7:	8.98	0	0	0.000002	(0.0006070.000142-0.001440)
8:	18.22	0.000037	0.19	0.000637	(-0.000408-0.0029050.025070)
9:	19.27	0.000002	0.01	0.000026	(-0.0049590.0003220.001202)
10:	22.34	0.000026	0.13	0.000369	(-0.0001620.0191670.001379)
11:	24.91	0.000007	0.03	0.000083	(-0.000195-0.000188-0.009093)
12:	26.22	0.000001	0.01	0.000016	(0.0036130.001446-0.000646)
13:	31.1	0.000013	0.06	0.000129	(0.005448-0.0098530.001348)
14:	34.03	0.000006	0.03	0.00006	(-0.0023880.0027890.006789)
15:	38.1	0.000004	0.02	0.000031	(0.00359-0.0041940.000796)
16:	40.93	0	0	0.000002	(0.00025-0.000617-0.001411)
17:	52.11	0.000012	0.06	0.00007	(-0.005006-0.006646-0.000848)
18:	53.81	0.000004	0.02	0.000021	(-0.000037-0.0022270.004034)
19:	55.6	0.000006	0.03	0.000036	(-0.003493-0.004735-0.001195)
20:	62.5	0.000013	0.07	0.000067	(-0.000977-0.00167-0.007943)
21:	70.67	0.000212	1.07	0.000937	(0.0048890.0301220.002396)
22:	85.23	0.000024	0.12	0.000088	(-0.000474-0.0011280.009291)
23:	90.7	0.000323	1.63	0.00111	(-0.0001430.0332570.001991)
24:	107.14	0.000071	0.36	0.000207	(-0.001504-0.0000780.014315)
25:	108.31	0.000069	0.35	0.000198	(-0.0106640.009106-0.001010)
26:	110.29	0.000001	0.01	0.000003	(-0.0010430.0007630.001288)
27:	113.63	0.000002	0.01	0.000006	(0.000217-0.0025090.000275)
28:	120.8	0.000149	0.75	0.000385	(-0.005743-0.01875-0.000638)
29:	136.24	0.000221	1.12	0.000506	(-0.009493-0.020355-0.001205)
30:	147.97	0.000528	2.67	0.001114	(0.0183360.027630.003813)
31:	148.83	0.000065	0.33	0.000135	(0.009991-0.005911-0.000710)

32:	152.9	0.00011	0.56	0.000225	(0.0006310.001725-0.014904)
33:	161.38	0.000226	1.14	0.000436	(0.0009150.002113-0.020761)
34:	166.27	0.000461	2.33	0.000866	(0.008520.008452-0.026870)
35:	166.5	0.000267	1.35	0.0005	(-0.008578-0.007225-0.019344)
36:	172.9	0.000045	0.23	0.000082	(-0.000679-0.0009330.008968)
37:	176.45	0.000675	3.41	0.001194	(0.0176880.02964-0.001599)
38:	177.63	0.000579	2.93	0.001018	(0.0144440.0280630.004629)
39:	183.68	0.000871	4.4	0.00148	(0.01973-0.03294-0.002466)
40:	185.41	0.000082	0.42	0.000139	(0.010471-0.004652-0.002749)
41:	190.83	0.000336	1.7	0.000549	(-0.0002760.003324-0.023199)
42:	205.67	0.002088	10.55	0.003169	(0.0487620.0280640.001836)
43:	221.31	0.001757	8.88	0.002477	(0.0457860.0188950.004851)
44:	223.69	0.002043	10.32	0.00285	(0.0049260.006532-0.052758)
45:	237.84	0.000028	0.14	0.000037	(-0.005991-0.000882-0.000282)
46:	240.03	0.000004	0.02	0.000006	(-0.000456-0.0004120.002288)
47:	251.76	0.000419	2.12	0.00052	(-0.0003310.000372-0.022793)
48:	260.15	0.000871	4.4	0.001044	(0.0310150.0088250.002159)
49:	269.28	0.002949	14.91	0.003418	(0.0013720.002559-0.058393)
50:	302.72	0.001289	6.51	0.001328	(0.0141870.0334920.002318)
51:	312.08	0.018165	91.8	0.018164	(0.0014260.1344970.008515)
52:	331.39	0.00097	4.9	0.000913	(-0.0067050.029248-0.003579)
53:	331.89	0.000868	4.39	0.000816	(-0.0018140.0030720.028346)
54:	348.77	0.000671	3.39	0.0006	(-0.0054740.017-0.016764)
55:	349.38	0.005181	26.18	0.004628	(0.026344-0.062277-0.007459)
56:	365.62	0.000562	2.84	0.00048	(0.003899-0.0031630.021316)
57:	367.86	0.055092	278.41	0.046735	(-0.206767-0.061147-0.015624)
58:	372.8	0.0126	63.68	0.010548	(-0.1014830.015615-0.002191)
59:	382.25	0.057818	292.19	0.047202	(0.0129810.016233-0.216265)
60:	444.16	0.00029	1.47	0.000204	(-0.0129910.0059060.000276)
61:	483.86	0.00772	39.02	0.004979	(-0.0013140.0703910.004746)
62:	487.73	0.002656	13.42	0.001699	(-0.000734-0.0024720.041143)
63:	498.22	0.001346	6.8	0.000843	(-0.024296-0.015829-0.001412)
64:	504.14	0.001402	7.08	0.000868	(0.0007580.001975-0.029379)
65:	523.79	0.00009	0.46	0.000054	(-0.000808-0.0007040.007263)
66:	524.04	0.000014	0.07	0.000008	(-0.002299-0.000208-0.001762)
67:	546.49	0.005366	27.12	0.003064	(0.0069450.0547970.003668)
68:	559.91	0.000404	2.04	0.000225	(0.0107370.0104420.001036)
69:	563.36	0.000195	0.99	0.000108	(0.0004590.000635-0.010369)
70:	575.36	0.002536	12.81	0.001375	(-0.0031430.0369390.000935)
71:	575.7	0.002895	14.63	0.001569	(0.0008350.001842-0.039563)
72:	596.39	0.000002	0.01	0.000001	(-0.0004950.000577-0.000759)

73:	597.23	0.000002	0.01	0.000001	(0.0001510.000004-0.001026)
74:	597.83	0.000003	0.01	0.000001	(-0.0009780.000612-0.000354)
75:	600.28	0.000019	0.1	0.00001	(-0.001743-0.002562-0.000690)
76:	603.97	0.000008	0.04	0.000004	(-0.001175-0.000879-0.001327)
77:	604.32	0.000053	0.27	0.000027	(-0.004057-0.00331-0.000007)
78:	606.43	0.003219	16.27	0.001656	(-0.002052-0.040522-0.003194)
79:	612.29	0.002702	13.66	0.001377	(0.0007520.002683-0.037008)
80:	739.75	0.000422	2.13	0.000178	(-0.0011720.0132320.001244)
81:	750.12	0.00331	16.73	0.001377	(0.0006070.002005-0.037048)
82:	771.86	0.000207	1.05	0.000084	(-0.009034-0.001423-0.000170)
83:	783.05	0.000543	2.74	0.000216	(-0.001263-0.0020490.014511)
84:	787.39	0.005875	29.69	0.002329	(-0.017320-0.0257890.036925)
85:	787.9	0.0091	45.99	0.003604	(0.0332830.044140.023410)
86:	805.83	0.003379	17.08	0.001309	(-0.017923-0.031046-0.004857)
87:	808.2	0.000597	3.02	0.00023	(0.005650.002831-0.013801)
88:	812.55	0.015261	77.12	0.005861	(0.0042510.025169-0.072177)
89:	812.82	0.008343	42.16	0.003203	(-0.0183700.0374440.038259)
90:	814.48	0.074608	377.04	0.028586	(0.1677270.0178370.011619)
91:	815.31	0.006316	31.92	0.002418	(-0.011501-0.0064120.047372)
92:	820.16	0.015823	79.96	0.006021	(0.0359580.0681670.008994)
93:	824.06	0.051158	258.53	0.019373	(0.0032240.013357-0.138508)
94:	831.18	0.018869	95.36	0.007084	(0.029403-0.078537-0.007201)
95:	853.17	0.000138	0.7	0.000051	(0.0007490.0003660.007061)
96:	853.53	0.000069	0.35	0.000025	(0.0036840.003142-0.001357)
97:	854.84	0.000006	0.03	0.000002	(0.0003720.0003410.001333)
98:	855.6	0.000015	0.08	0.000006	(-0.002317-0.000332-0.000257)
99:	857.64	0.000011	0.06	0.000004	(0.000436-0.0005030.001924)
100:	858.37	0.000048	0.24	0.000017	(0.002217-0.003515-0.000105)
101:	869.48	0.002021	10.21	0.000725	(0.0071680.0259060.001661)
102:	871.83	0.009533	48.18	0.003412	(-0.013418-0.056711-0.004021)
103:	887.63	0.004197	21.21	0.001476	(-0.000904-0.0024710.038325)
104:	907.39	0.000031	0.16	0.000011	(-0.000202-0.000233-0.003254)
105:	909.42	0.000004	0.02	0.000001	(-0.0010620.0005260.000144)
106:	910.33	0.000014	0.07	0.000005	(0.0011040.000314-0.001835)
107:	910.95	0.000009	0.05	0.000003	(-0.001408-0.0010190.000234)
108:	916.46	0.000058	0.29	0.00002	(-0.002374-0.0035110.001381)
109:	917.06	0.000119	0.6	0.00004	(0.0034510.0051720.001320)
110:	921.00	0.008718	44.05	0.002954	(-0.001138-0.003590.054218)
111:	927.62	0.000249	1.26	0.000084	(-0.0018650.0033570.008315)
112:	928.50	0.000255	1.29	0.000086	(0.003435-0.006480.005654)
113:	930.52	0.001635	8.27	0.000548	(-0.0161420.016955-0.000671)

114:	930.95	0.000759	3.84	0.000255	(0.001694-0.000577-0.015854)
115:	983.14	0.003612	18.26	0.001147	(-0.005163-0.033391-0.002233)
116:	996.23	0.003047	15.4	0.000955	(0.0007770.002807-0.030758)
117:	997.32	0.0003	1.51	0.000094	(0.0005460.002476-0.009344)
118:	998.08	0.000276	1.39	0.000086	(0.002340.0084350.003103)
119:	1000.74	0.001057	5.34	0.00033	(-0.000897-0.01801-0.002115)
120:	1024.43	0.004458	22.53	0.001358	(0.004706-0.000173-0.036550)
121:	1024.57	0.000492	2.49	0.00015	(0.00178-0.0042640.011335)
122:	1025.23	0.003313	16.74	0.001008	(-0.0214630.022998-0.004335)
123:	1025.88	0.002888	14.59	0.000878	(-0.002206-0.0008910.029542)
124:	1026.28	0.000892	4.51	0.000271	(0.0094510.0083480.010597)
125:	1026.47	0.002075	10.49	0.000631	(-0.018036-0.0174040.001646)
126:	1026.72	0.002645	13.37	0.000804	(0.0047680.0278840.001938)
127:	1044.64	0.000381	1.92	0.000114	(-0.000273-0.000811-0.010630)
128:	1045.31	0.000196	0.99	0.000058	(0.0028230.006658-0.002481)
129:	1049.31	0.000094	0.48	0.000028	(-0.0001670.000668-0.005246)
130:	1049.77	0.003535	17.87	0.001051	(-0.007286-0.031518-0.002109)
131:	1051.89	0.001998	10.1	0.000593	(0.0020630.0241140.002632)
132:	1053.86	0.000044	0.22	0.000013	(-0.0001410.001776-0.003116)
133:	1068.88	0.000022	0.11	0.000006	(-0.0000400.000105-0.002519)
134:	1070.39	0.000115	0.58	0.000034	(0.0010440.005707-0.000107)
135:	1071.42	0.000005	0.02	0.000001	(-0.000342-0.000731-0.000852)
136:	1071.54	0.000002	0.01	0.000001	(0.0002980.000045-0.000743)
137:	1073.35	0.000018	0.09	0.000005	(-0.0006520.002054-0.000788)
138:	1074.12	0.001269	6.41	0.000369	(-0.0047400.0185860.000876)
139:	1111.64	0.000573	2.89	0.000161	(-0.0109980.0063090.000144)
140:	1114.01	0.007257	36.67	0.002033	(-0.000819-0.0029670.044983)
141:	1118.03	0.000773	3.91	0.000216	(0.0001810.0146780.000649)
142:	1132.36	0.00048	2.43	0.000132	(-0.000581-0.002020.011309)
143:	1149.19	0.003266	16.5	0.000887	(0.0016040.001534-0.029696)
144:	1149.65	0.002423	12.25	0.000658	(0.025213-0.0044490.001499)
145:	1150.96	0.003496	17.67	0.000948	(-0.015921-0.026304-0.001599)
146:	1168.83	0.055927	282.63	0.014932	(0.0275340.1187830.008019)
147:	1177.27	0.000091	0.46	0.000024	(0.0034440.001735-0.003062)
148:	1177.36	0.00041	2.07	0.000109	(0.0095510.0039330.001451)
149:	1207.82	0.002461	12.44	0.000636	(-0.000383-0.0013750.025174)
150:	1254.84	0.014192	71.72	0.003529	(0.0011450.004013-0.059262)
151:	1259.97	0.002944	14.88	0.000729	(0.0013630.0268720.002255)
152:	1265.88	0.000373	1.89	0.000092	(0.0001350.000384-0.009583)

153:	1266.53	0.000236	1.19	0.000058	(0.0016320.0074540.000136)
154:	1278.70	0.000017	0.08	0.000004	(0.0003470.001694-0.001028)
155:	1278.93	0.000109	0.55	0.000027	(0.0003310.0050670.000847)
156:	1279.10	0.000001	0	0	(-0.0000010.000037-0.000357)
157:	1280.95	0.000013	0.07	0.000003	(-0.0002210.001776-0.000152)
158:	1281.25	0.001689	8.54	0.000411	(0.002559-0.020114-0.000519)
159:	1297.43	0.025741	130.08	0.006191	(-0.001557-0.0046550.078532)
160:	1306.01	0.000932	4.71	0.000223	(0.002370.0146770.001284)
161:	1338.43	0.000054	0.27	0.000013	(0.000519-0.000918-0.003387)
162:	1338.75	0.000082	0.41	0.000019	(-0.0019320.003858-0.000709)
163:	1362.57	0.003616	18.27	0.000828	(-0.000469-0.0016520.028727)
164:	1384.77	0.000813	4.11	0.000183	(-0.000037-0.000465-0.013529)
165:	1390.22	0.012859	64.98	0.002886	(-0.011514-0.052378-0.003213)
166:	1393.28	0.000296	1.5	0.000066	(-0.0007180.001808-0.007905)
167:	1394.78	0.000781	3.95	0.000175	(-0.0113310.0067330.000980)
168:	1397.97	0.000754	3.81	0.000168	(-0.000317-0.0012630.012908)
169:	1400.68	0.000044	0.22	0.00001	(-0.003050-0.000389-0.000633)
170:	1401.76	0.000008	0.04	0.000002	(-0.0003990.0009970.000860)
171:	1402.42	0.00021	1.06	0.000047	(-0.0029720.0061640.000004)
172:	1404.18	0.000005	0.02	0.000001	(-0.000716-0.0004960.000540)
173:	1404.43	0.000005	0.03	0.000001	(0.000410.0009550.000377)
174:	1405.43	0.000003	0.01	0.000001	(-0.0006030.000122-0.000469)
175:	1405.84	0.000007	0.03	0.000001	(-0.0010340.00064-0.000111)
176:	1440.22	0.002091	10.57	0.000453	(-0.000704-0.0021540.021167)
177:	1440.82	0.000955	4.83	0.000207	(-0.000513-0.014154-0.002493)
178:	1443.07	0.001364	6.89	0.000295	(-0.0159690.006301-0.000487)
179:	1445.86	0.000047	0.24	0.00001	(-0.000145-0.0004850.003139)
180:	1448.79	0.000917	4.64	0.000198	(0.0099260.0099460.000354)
181:	1453.26	0.000376	1.9	0.000081	(0.000066-0.000637-0.008964)
182:	1455.22	0.001947	9.84	0.000418	(0.0190580.007250.001337)
183:	1456.23	0.00046	2.33	0.000099	(0.0024090.001339-0.009542)
184:	1459.87	0.000661	3.34	0.000141	(-0.0017240.011761-0.000073)
185:	1470.38	0.000051	0.26	0.000011	(-0.0025380.0001290.002103)
186:	1471.02	0.001389	7.02	0.000295	(0.0147270.00822-0.003187)
187:	1471.32	0.001069	5.4	0.000227	(0.0044240.0019850.014252)
188:	1472.96	0.001025	5.18	0.000217	(-0.0045770.0137350.002753)
189:	1473.05	0.002275	11.5	0.000482	(-0.0050880.021352-0.000392)
190:	1474.11	0.000681	3.44	0.000144	(0.0005930.0119450.001057)
191:	1484.52	0.002895	14.63	0.000609	(-0.000627-0.0014320.024620)

192:	1493.43	0.000641	3.24	0.000134	(-0.0045430.0106410.000318)
193:	1497.01	0.025338	128.05	0.005282	(0.0011980.004886-0.072502)
194:	1500.93	0.000954	4.82	0.000198	(0.000815-0.013784-0.002756)
195:	1506.30	0.000353	1.78	0.000073	(-0.000381-0.0005430.008520)
196:	1506.72	0.00304	15.36	0.00063	(0.023299-0.009309-0.000333)
197:	1635.02	0.000032	0.16	0.000006	(0.001856-0.001303-0.001008)
198:	1635.60	0.000248	1.25	0.000047	(-0.0056310.00395-0.000217)
199:	1646.84	0.001374	6.94	0.00026	(0.000002-0.0014150.016075)
200:	1647.77	0.000166	0.84	0.000031	(0.00377-0.003742-0.001772)
201:	2932.79	0.001192	6.02	0.000127	(0.000368-0.0000660.011254)
202:	2939.06	0.031515	159.26	0.003346	(-0.042171-0.03947-0.003144)
203:	3004.01	0.004121	20.83	0.000428	(0.001888-0.020575-0.001109)
204:	3008.44	0.010193	51.51	0.001057	(-0.000621-0.0019370.032453)
205:	3011.74	0.003928	19.85	0.000407	(-0.0034040.0025780.019718)
206:	3014.99	0.003569	18.04	0.000369	(0.012796-0.0136420.004423)
207:	3024.69	0.007339	37.09	0.000757	(0.013716-0.023829-0.001111)
208:	3027.83	0.000935	4.72	0.000096	(0.006422-0.0073670.000903)
209:	3028.20	0.013693	69.2	0.001411	(-0.0262330.0268270.001807)
210:	3074.76	0.009333	47.17	0.000947	(0.0155410.026490.002006)
211:	3086.71	0.001374	6.94	0.000139	(0.0033690.000770.011266)
212:	3088.03	0.007191	36.34	0.000727	(-0.024545-0.0111390.000432)
213:	3093.32	0.001087	5.49	0.00011	(-0.003315-0.006691-0.007342)
214:	3093.41	0.004416	22.32	0.000446	(-0.007323-0.0197130.001824)
215:	3122.15	0.004517	22.83	0.000451	(-0.0080470.004588-0.019123)
216:	3122.45	0.003551	17.95	0.000355	(-0.0126800.0042380.013272)
217:	3123.31	0.002717	13.73	0.000271	(0.002-0.00484-0.015620)
218:	3124.70	0.002337	11.81	0.000233	(0.00426-0.0135790.005559)
219:	3160.12	0.000388	1.96	0.000038	(-0.0028020.0011580.005394)
220:	3160.30	0.000227	1.15	0.000022	(0.002423-0.0015830.003747)
221:	3167.40	0.003867	19.54	0.000381	(-0.0156730.0086420.007786)
222:	3167.54	0.004075	20.59	0.000401	(0.01596-0.0098960.006987)
223:	3186.47	0.008253	41.71	0.000808	(-0.000330-0.0019760.028360)
224:	3186.52	0.000391	1.97	0.000038	(-0.0023140.0055690.001374)
225:	3225.82	0.000002	0.01	0	(0.0001850.000118-0.000353)
226:	3226.17	0.00002	0.1	0.000002	(0.0005980.000445-0.001178)
227:	3226.29	0.000016	0.08	0.000002	(0.0005210.0003680.001079)
228:	3226.49	0.000009	0.05	0.000001	(-0.0007720.000493-0.000277)
229:	3230.52	0.000187	0.95	0.000018	(0.001049-0.0035350.002117)
230:	3230.60	0.000256	1.29	0.000025	(-0.0015450.0043410.001866)

231:	3239.80	0.000057	0.29	0.000005	(0.0000840.000216-0.002323)
232:	3241.65	0.000002	0.01	0	(0.0002590.000140.000242)
233:	3241.83	0.000379	1.92	0.000037	(0.0013710.0055150.002057)
234:	3241.98	0.000427	2.16	0.000041	(0.0014310.006049-0.001584)
235:	3245.06	0.000112	0.57	0.000011	(-0.0003910.0029510.001378)
236:	3245.11	0.000081	0.41	0.000008	(0.000251-0.0024340.001347)
237:	3251.57	0.000284	1.44	0.000027	(-0.004200-0.003102-0.000161)
238:	3256.12	0.000657	3.32	0.000063	(-0.0028130.007305-0.001311)
239:	3256.21	0.000327	1.65	0.000031	(0.001788-0.004539-0.002739)

GIBBS FREE ENERGY

The Gibbs free energy is G = H - T*S

Total enthalpy ... -2637.83988895 Eh

Total entropy correction ... -0.11603936 Eh -72.82 kcal/mol

Final Gibbs free energy ... -2637.95592831 Eh

For completeness - the Gibbs free energy minus the electronic energy

G-E(el) ... 0.57168747 Eh 358.74 kcal/mol

References:

1. M. R. Willcott, MestRe Nova, *J. Am. Chem. Soc.*, 2009, **131**, 13180.
2. Bruker, APEX4, 2021.
3. Bruker, SAINT, 2012.
4. G. M. Sheldrick, SADABS, 1996, Program for Area Detector Absorption Correction.
5. Bruker, Bruker SHELXTL.
6. O. V. Dolomanov, L. J. Bourhis, R. J. Gildea, J. A. K. Howard, H. Puschmann, O. V. Dolomanov, L. J. Bourhis, R. J. Gildea, J. A. K. Howard and H. Puschmann, OLEX2: a complete structure solution, refinement and analysis program, *J. Appl. Cryst.*, 2009, **42**, 339-341.
7. G. M. Sheldrick, Crystal structure refinement with SHELXL, *Acta Cryst. C*, 2015, **71**, 3-8.
8. C. Ganesamoorthy, S. Loerke, C. Gemel, P. Jerabek, M. Winter, G. Frenking and R. A. Fischer, Reductive elimination: a pathway to low-valent aluminium species, *Chem. Commun.*, 2013, **49**, 2858-2860.
9. N. E. Travia, M. J. Monreal, B. L. Scott and J. L. Kiplinger, Thorium-mediated ring-opening of tetrahydrofuran and the development of a new thorium starting material: preparation and chemistry of $ThI_4(DME)_2$, *Dalton Trans.*, 2012, **41**, 14514-14523.
10. C. Schädle, C. Meermann, K. W. Törnroos and R. Anwander, Rare-Earth Metal Phenyl(trimethylsilyl)amide Complexes, *Euro. J. Inorg. Chem.*, 2010, **2010**, 2841-2852.
11. J. D. Scollard, D. H. McConville and J. J. Vittal, Bulky Chelating Diamide Complexes of Zirconium: Synthesis, Structure, and Reactivity of d0 Alkyl Derivatives, *Organometallics*, 1997, **16**, 4415-4420.
12. Y. K. Loh, L. Ying, M. Á. Fuentes, D. C. H. Do and S. Aldridge, An N-Heterocyclic Boryloxy Ligand Isoelectronic with N-Heterocyclic Imines: Access to an Acyclic Dioxysilylene and its Heavier Congeners, *Angew. Chem. Int. Ed.*, 2019, **58**, 4847-4851.
13. D. Weismann, D. Saurenz, R. Boese, D. Bläser, G. Wolmershäuser, Y. Sun and H. Sitzmann, Cyclopentadiene Alkylation and Nickel Complexes with Tri-, Tetra-, or Pentaisopropylcyclopentadienide or an Even Bulkier Lithium Alkylcyclopentadienide, *Organometallics*, 2011, **30**, 6351-6364.
14. T. Chlupatý, Z. Padělková, A. Lyčka and A. Růžička, Structure and properties of lithium *n*-butyl amidinates, *J. Organomet. Chem.*, 2011, **696**, 2346-2354.
15. H. Sitzmann, M. F. Lappert, C. Dohmeier, C. Üffing and H. Schnöckel, Cyclopentadienylderivate von Aluminium(I), *J. Organomet. Chem.*, 1998, **561**, 203-208.
16. D. Sarkar, P. Vasko, A. F. Roper, A. E. Crumpton, M. M. D. Roy, L. P. Griffin, C. Bogle and S. Aldridge, Reversible [4 + 1] Cycloaddition of Arenes by a "Naked" Acyclic Aluminyl Compound, *J. Am. Chem. Soc.*, 2024, **146**, 11792–11800.
17. O. Kysliak, H. Görls and R. Kretschmer, Salt metathesis as an alternative approach to access aluminium(I) and gallium(I) β-diketiminates, *Dalton Trans.*, 2020, **49**, 6377-6383.
18. C. Cui, H. W. Roesky, H. G. Schmidt, M. Noltemeyer, H. Hao and F. Cimpoesu, Synthesis and Structure of a Monomeric Aluminum(I) Compound [{$HC(CMeNAr)_2$}Al] (Ar=2,6–

i$Pr_2C_6H_3$): A Stable Aluminum Analogue of a Carbene, *Angew. Chem. Int. Ed.*, 2000, **39**, 4274-4276.
19. M. Schiefer, N. Dastagiri Reddy, Herbert W. Roesky and D. Vidovic, Synthesis and Structural Characterization of an Exclusively N-Based Tetrameric Aluminum(I) Compound, *Organometallics*, 2003, **22**, 3637-3638.
20. G. F. Feng, K. L. Chan, Z. Y. Lin and M. Yamashita, Al-Sc Bonded Complexes: Synthesis, Structure, and Reaction with Benzene in the Presence of Alkyl Halide, *J. Am. Chem. Soc.*, 2022, **144**, 22662-22668.
21. G. F. Feng, K. L. Chan, Z. Y. Lin and M. Yamashita, Alumanyl-Samarium(II): Synthesis, Characterization, and Reactivity Studies, *J. Am. Chem. Soc.*, 2024, **146**, 7204-7209.
22. R. D. Shannon, Revised effective ionic radii and systematic studies of interatomic distances in halides and chalcogenides, *Acta Cryst.*, 1976, **32**, 751-767.
23. G. M. Sheldrick and W. S. Sheldrick, Crystal and molecular structure of tris[bis(trimethylsilyl)amino]aluminium, $Al[N(SiMe_3)_2]_3$, *J. Chem. Soc. A*, 1969, 2279-2282.
24. J. S. Ghotra, M. B. Hursthouse and A. J. Welch, Three-co-ordinate scandium(III) and europium(III); crystal and molecular structures of their trishexamethyldisilylamides, *J. Chem. Soc., Chem. Commun*, 1973, 669-670.
25. D. H. Woen, G. P. Chen, J. W. Ziller, T. J. Boyle, F. Furche and W. J. Evans, Solution Synthesis, Structure, and CO2 Reduction Reactivity of a Scandium(II) Complex, $\{Sc[N(SiMe_3)_2]_3\}^-$, *Angew. Chem. Int. Ed.*, 2017, **56**, 2050-2053.
26. M. Westerhausen, M. Hartmann, A. Pfitzner and W. Schwarz, Bis(trimethylsilyl)amide und -methanide des Yttriums — Molekülstrukturen von Tris(diethylether-O)lithium-(μ-chloro)-tris[bis(trimethylsilyl) methyl]yttriat, solvensfreiem Yttrium-tris[bis(trimethylsilyl)amid] sowie dem Bis(benzonitril)-Komplex, *Z. Anorg. Allg. Chem.*, 1995, **621**, 837-850.
27. L. Wursthorn, K. Beckett, J. O. Rothbaum, R. M. Cywar, C. Lincoln, Y. Kratish and T. J. Marks, Selective Lanthanide-Organic Catalyzed Depolymerization of Nylon-6 to ϵ-Caprolactam, *Angew. Chem. Int. Ed.*, 2023, **62**, e202212543.
28. F. Neese, Software Update: The ORCA Program System—Version 6.0, *Wiley Interdiscip. Rev. Comput. Mol. Sci.*, 2025, **15**, e70019.
29. S. Grimme, J. Antony, S. Ehrlich and H. Krieg, A consistent and accurate ab initio parametrization of density functional dispersion correction (DFT-D) for the 94 elements H-Pu, *J. Chem. Phys.*, 2010, **132**, 154104.
30. A. D. Becke, Density-functional thermochemistry. III. The role of exact exchange, *J. Chem. Phys.*, 1993, **98**, 5648–5652.
31. A. D. Becke and K. E. Edgecombe, A simple measure of electron localization in atomic and molecular systems, *J. Chem. Phys.*, 1990, **92**, 5397–5403.
32. C. Lee, W. Yang and R. G. Parr, Development of the Colle-Salvetti correlation-energy formula into a functional of the electron density, *Phys. Rev. B*, 1988, **37**, 785.
33. F. Weigend and R. Ahlrichs, Balanced basis sets of split valence, triple zeta valence and quadruple zeta valence quality for H to Rn: Design and assessment of accuracy, *Phys. Chem. Chem. Phys.*, 2005, **7**, 3297-3305.
34. F. Weigend, Accurate Coulomb-fitting basis sets for H to Rn, *Phys. Chem. Chem. Phys.*, 2006, **8**, 1057-1065.

35. F. Neese, An improvement of the resolution of the identity approximation for the formation of the Coulomb matrix, *J. Comput. Chem.*, 2003, **24**, 1740-1747.
36. F. Neese, F. Wennmohs, A. Hansen and U. Becker, Efficient, approximate and parallel Hartree–Fock and hybrid DFT calculations. A 'chain-of-spheres' algorithm for the Hartree–Fock exchange, *Chem. Phys.*, 2009, **356**, 98-109.
37. S. Grimme, S. Ehrlich and L. Goerigk, Effect of the damping function in dispersion corrected density functional theory, *J. Comput. Chem.*, 2011, **32**, 1456-1465.
38. D. Bykov, T. Petrenko, R. Izsák, S. Kossmann, U. Becker, E. Valeev and F. Neese, Efficient implementation of the analytic second derivatives of Hartree–Fock and hybrid DFT energies: a detailed analysis of different approximations, *Mol. Phys.*, 2015, **113**, 1961-1977.
39. B. Helmich-Paris, B. de Souza, F. Neese and R. Izsák, An improved chain of spheres for exchange algorithm, *J. Chem. Phys.*, 2021, **155**, 104109.
40. F. Neese, The SHARK integral generation and digestion system, *J. Comput. Chem.*, 2023, **44**, 381-396.
41. T. Lu and F. Chen, Multiwfn: A multifunctional wavefunction analyzer, *J. Comput. Chem.*, 2012, **33**, 580-592.
42. R. F. W. Bader, A quantum theory of molecular structure and its applications, *Chem. Rev.*, 2002, **91**, 893–928.
43. L. Tian and C. Fei-Wu, Meaning and Functional Form of the Electron Localization Function, *Acta Phys. Chim. Sin.*, 2011, **27**, 2786-2792.
44. W. Humphrey, A. Dalke and K. Schulten, VMD: Visual molecular dynamics, *J. Mol. Graphics*, 1996, **14**, 33-38.
45. P. o. V. P. Ltd., Persistence of Vision Raytracer, 2004.
